# Multicentre, randomised controlled trial to investigate the effects of parental touch on relieving acute procedural pain in neonates (Petal)

**DOI:** 10.1136/bmjopen-2022-061841

**Published:** 2022-07-19

**Authors:** Maria M Cobo, Fiona Moultrie, Annalisa G V Hauck, Daniel Crankshaw, Vaneesha Monk, Caroline Hartley, Ria Evans Fry, Shellie Robinson, Marianne van der Vaart, Luke Baxter, Eleri Adams, Ravi Poorun, Aomesh Bhatt, Rebeccah Slater

**Affiliations:** 1 Department of Paediatrics, University of Oxford, Oxford, UK; 2 Colegio de Ciencias Biologicas y Ambientales, Universidad San Francisco de Quito USFQ, Quito, Ecuador; 3 Newborn Care Unit, John Radcliffe Hospital, Oxford University Hospitals NHS Foundation Trust, Oxford, UK; 4 Children’s Services, Royal Devon University Healthcare NHS Foundation Trust, Exeter, UK; 5 College of Medicine & Health, University of Exeter, Exeter, UK

**Keywords:** neonatology, neurophysiology, pain management

## Abstract

**Introduction:**

Newborn infants routinely undergo minor painful procedures as part of postnatal care, with infants born sick or premature requiring a greater number of procedures. As pain in early life can have long-term neurodevelopmental consequences and lead to parental anxiety and future avoidance of interventions, effective pain management is essential. Non-pharmacological comfort measures such as breastfeeding, swaddling and sweet solutions are inconsistently implemented and are not always practical or effective in reducing the transmission of noxious input to the brain. Stroking of the skin can activate C-tactile fibres and reduce pain, and therefore could provide a simple and safe parent-led intervention for the management of pain. The trial aim is to determine whether parental touch prior to a painful clinical procedure provides effective pain relief in neonates.

**Methods and analysis:**

This is a multicentre randomised controlled trial. A total of 112 neonates born at 35 weeks’ gestation or more requiring a blood test in the first week of life will be recruited and randomised to receive parental stroking either preprocedure or postprocedure. We will record brain activity (EEG), cardiac and respiratory dynamics, oxygen saturation and facial expression to provide proxy pain outcome measures. The primary outcome will be the reduction of noxious-evoked brain activity in response to a heel lance. Secondary outcomes will be a reduction in clinical pain scores (Premature Infant Pain Profile-Revised), postprocedural tachycardia and parental anxiety.

**Ethics and dissemination:**

The study has been approved by the London—South East Research Ethics Committee (ref: 21/LO/0523). The results will be widely disseminated through peer-reviewed publications, international conferences and via our partner neonatal charities Bliss and Supporting the Sick Newborn And their Parents (SSNAP). If the parental tactile intervention is effective, recommendations will be submitted via the National Health Service clinical guideline adoption process.

**Study status:**

Commenced September 2021.

**Trial registration number:**

NCT04901611; 14 135 962.

**Strengths and limitations of this study**

- Petal is a randomised controlled trial investigating whether noxious-evoked brain activity is reduced by preprocedural parental stroking.
- The trial is based on published evidence from two mechanistic studies which show a reduction in noxious-evoked brain activity during a heel lance or experimental stimuli in neonates whose skin was brushed by the experimenter prior to the procedure.
- This trial investigates stroking as a simple, free, low-risk, non-pharmacological pain-relieving intervention delivered by parents to their newborn infants in the first week of life.
- The trial employs multiple proxy measures to determine the impact of the stroking intervention on neonatal pain and investigates the impact of the intervention on parental anxiety and distress.
- While investigators cannot be blinded to the group allocation at the time of the study, this limitation is mitigated by ensuring that participants and investigators involved in all other aspects of the trial, including data analysis, are blinded.

## INTRODUCTION

### Background

Newborn infants undergo painful procedures as part of routine neonatal care. Sick or premature infants experience an average of 10 painful procedures per day as part of life-sustaining treatment.^1^ It is recognised that repetitive exposure to pain in early life can cause short-term physiological instability as well as long-term neurodevelopmental consequences such as reduced growth, altered structural and functional brain development and reduced school-age academic performance.^2^ Furthermore, repeatedly witnessing their infant in pain can have a significant negative impact on the emotional and psychological well-being of parents.^3–5^ Effective pain management is therefore essential in neonatal care. However, measuring pain in this non-verbal patient population is challenging, and few safe and effective analgesics have been tested and approved for use in infants. Non-pharmacological strategies have been introduced and promoted over the last few decades for the management of acute procedural pain. Sweet-taste solutions such as sucrose are effective in relieving behavioural responses following minor painful procedures,^6^ but do not reduce noxious input to the brain.^7^ This has caused concern that this intervention may not mitigate the long-term consequences of early life pain, and furthermore, it may have long-term neurodevelopmental effects with repeated use.^8–10^ Breastfeeding also reduces behavioural and physiological responses to pain in full-term infants undergoing heel lancing, intramuscular injection and venepuncture.^11^ However, this strategy can be challenging for new mothers and is not always practical to implement in premature and critically ill infants or in mothers with transmissible infections. Other comfort measures include swaddling and facilitated tucking of infants, which, although useful, are less effective in reducing pain.^12^ While many studies have reported the potential pain-relieving effects of tactile interventions such as skin-to-skin care^13^ and massage^14–21^ in the context of minor painful procedures, these non-pharmacological interventions are scarcely used in maternity and neonatal units^22 23^ ^22^ and the mechanisms underpinning their effectiveness are still being established. Despite guidelines recommending the use of non-pharmacological interventions for pain relief, uptake of these practices remains poor and inconsistent.^23 24^

### Measuring pain in infants

The assessment of pain and analgesia in infants primarily relies on measuring changes in infant behaviour. One of the most common validated clinical pain tools is the Premature Infant Pain Profile (original PIPP, revised PIPP-R).^25 26^ While subjective evaluations of behavioural responses are a gold standard for the clinical assessment of neonatal pain, electrophysiology-based methods have more recently been developed to identify a pattern of noxious-evoked brain activity.^27–29^ This objective and quantifiable neurophysiological measure has been previously used in pilot studies^30 31^ and as the primary outcome measure in randomised clinical trials published in *The Lancet,* assessing the analgesic efficacy of sucrose^7^ and morphine.^32^ Noxious-evoked brain activity has specifically been well characterised in response to heel lancing,^27–29 33^ a clinical procedure which is frequently performed in neonates for blood collection, and will be used as the primary outcome of the Petal trial to investigate the efficacy of preprocedural parental stroking.

### Rationale

Maternal touch behaviours are instinctive, evolutionarily conserved among mammals.^34^ Previous studies suggest that there may also be a potential relationship between enhanced maternal touch and infant growth and development.^35 36^ Stroking, by repeatedly applying gentle pressure to the skin, can activate C-tactile (CT) fibres, a subclass of slow-conducting unmyelinated sensory neurons, mostly found in hairy skin.^37–39^ These fibres project to brain regions associated with affective processing such as the insular cortex, prefrontal cortex, superior temporal sulcus and cingulate cortex^40–44^ and are thought to have evolved to promote affiliative behaviours and social touch.^45–48^ CT-fibres are optimally activated by stroking at a velocity of 3 cm/s (optimal range 1–10 cm/s),^49–51^ and studies in adults of gentle brushing or stroking paradigms at this optimal velocity have demonstrated a reduction in pain ratings^52 53^ and noxious-evoked brain activity.^53^ CT-optimal stimulation therefore could provide a natural and safe pain-relieving intervention.

We previously conducted a small prospective cohort study of preprocedural stroking for pain relief in neonates, in which we demonstrated that CT-optimal stroking (at 3 cm/s) prior to an experimental noxious stimulus or clinical heel lance significantly reduced noxious-evoked brain activity in term neonates compared with no touch intervention.^30^ We replicated this study in an independent sample of term neonates and showed consistent results and a similar effect size in the group receiving the stroking intervention.^31^ However, in both of these studies, stroking was delivered by the researcher using a soft experimental brush with a known force. Although the studies did not identify a significant effect of the intervention on a clinical pain score, they were notably not powered to investigate this. Considering CT-optimal stroking is a natural maternal behaviour^54 55^ and evidence suggesting that CT-fibres respond optimally to touch at human skin temperature,^56^ hands-on parental stroking has the potential to provide even greater benefit than CT-optimal brushstrokes. Pilot work further suggests that stroking a neonate has similar efficacy to researcher-led experimental brushing (unpublished).

### Aim and objectives

In the Petal trial, we aim to determine whether parental stroking prior to a common painful clinical procedure (heel lancing) provides effective analgesia in neonates. The primary outcome will be the reduction of noxious-evoked brain activity during a heel lance. Secondary outcomes will be a reduction in clinical pain scores, postprocedural tachycardia and parental anxiety (table 1). Exploratory outcomes will investigate changes in brain activity during the intervention, as well as effects on physiological recovery postprocedure (using heart rate and respiratory dynamics) and further explore parental anxiety, distress, and attitudes to research.

**Table 1:** Objectives and outcome measures

Objectives	Outcome measures
**Primary objective** To test whether parental touch prior to the clinical procedure reduces noxious-evoked brain activity following a heel lance.	**Primary outcome measure** Magnitude of noxious-evoked brain activity following a heel lance (EEG data recorded in the 1000 ms period following each heel lance).
**Secondary objectives** To test whether parental touch prior to the clinical procedure reduces clinical pain scores (PIPP-R) during the 30 s period after the heel lance. To test whether parental touch prior to the clinical procedure reduces incidence of postprocedural tachycardia following a heel lance. To test whether parental touch prior to the clinical procedure reduces parental anxiety, compared with postprocedural touch.	**Secondary outcome measures** PIPP-R score during the 30 s period after the heel lance. Percentage of neonates who develop tachycardia in the 30 s post heel lance. Difference in STAI-S scores preprocedure and postprocedure.
**Exploratory objectives** To explore how parental touch impacts background brain activity. To explore whether parental touch prior to the clinical procedure reduces the duration of time for heart rate to return to baseline after a heel lance. To explore how parental touch prior to the clinical procedure affects respiratory stability. To explore parental anxiety and distress, and their experience of the trial and infant research.	**Exploratory outcome measures** Changes in brain activity during the touch intervention. Time taken for heart rate to return to baseline post heel lance. Postprocedural respiratory dynamics and incidence of apnoea. Scores for individual parameters from the STAI-T and STAI-S; four-point distress questionnaire score; responses to survey about participation in Petal and infant research.

EEG, Electroencephalography; PIPP-R, Premature Infant Pain Profile-Revised; STAI-S, State-Trait Anxiety Inventory-State; STAI-T, State-Trait Anxiety Inventory-Trait.

## Methods and analysis

### Trial description

**Figure 1:**
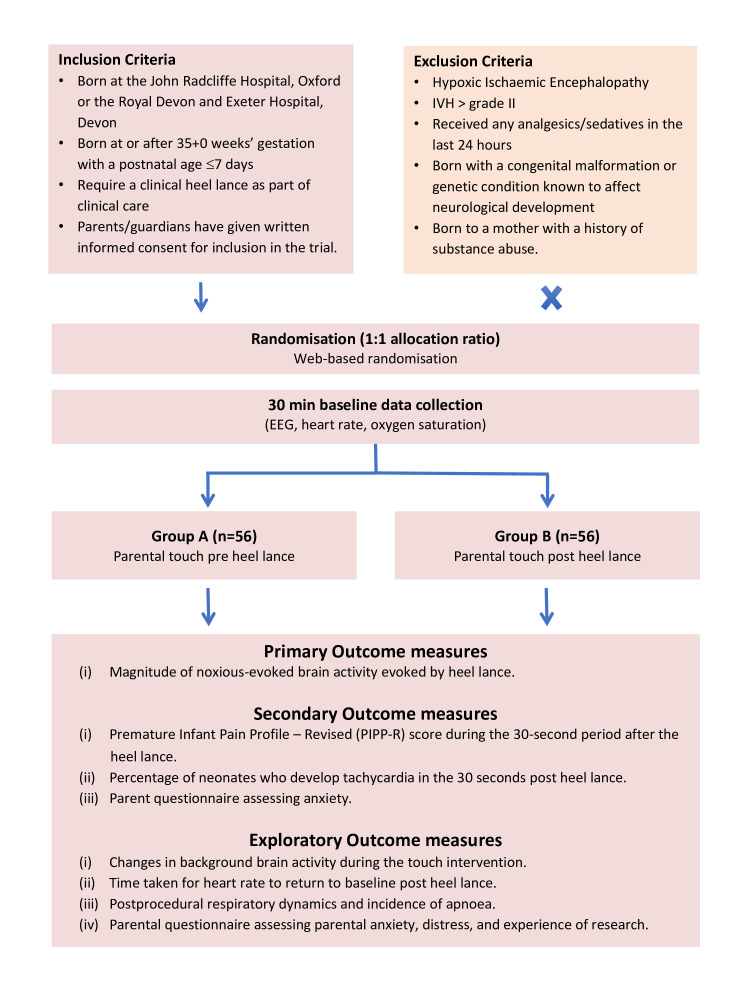
Trial flowchart. IVH, intraventricular haemorrhage.

This is a multicentre randomised controlled interventional trial, with two research sites (John Radcliffe Hospital, Oxford, and Royal Devon and Exeter Hospital, Devon, UK). The parents of eligible neonates satisfying inclusion criteria (figure 1) will be approached by a member of the research team. Parental written informed consent will be taken and neonates will be electronically randomised to receive parental stroking either prior to or after a clinically required heel lance. Patient information leaflets and consent forms are available as online supplemental file 1. A unique study ID will be assigned to each individual participant. The randomisation programme will use a minimisation algorithm to ensure approximate balance between the groups with respect to gestational age at birth, postnatal age at time of randomisation, sex, the indication for blood sampling and research site. The users of the system will be blind to the next allocation.

10.1136/bmjopen-2022-061841.supp1 Supplementary data

**Figure 2:**
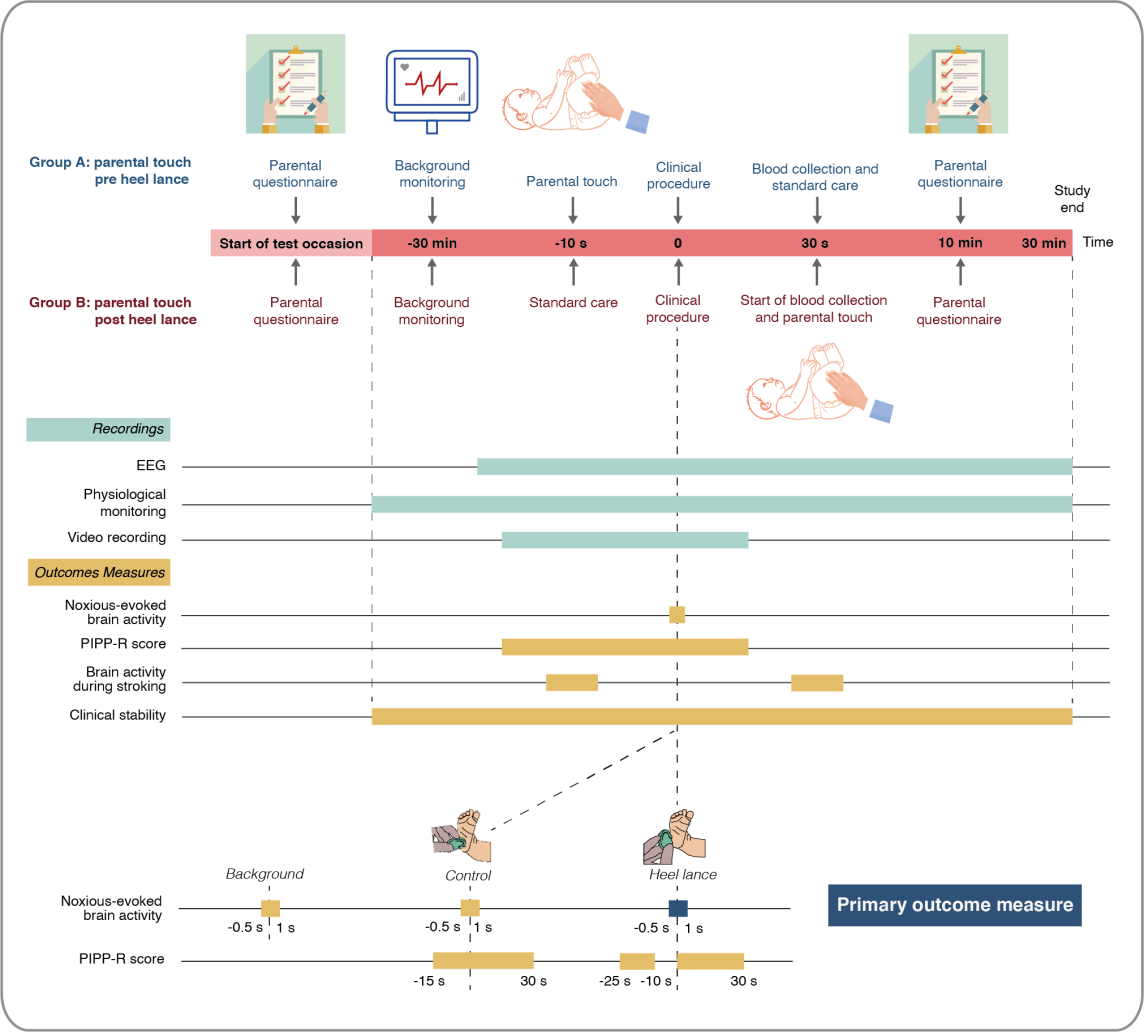
Trial procedures. EEG, electroencephalography; PIPP-R, Premature Infant Pain Profile-Revised.

Each neonate will be studied on a single test occasion lasting approximately 1 hour (figure 2) and will not require further follow-up. A parent will first complete the State-Trait Anxiety Inventory-Trait (STAI-T) and State-Trait Anxiety Inventory-State (STAI-S) questionnaires, which will be administered verbally by a researcher. This will allow assessment of both trait anxiety and state anxiety prior to the commencement of the stroking intervention or blood test. At least 30 min prior to the heel lance, the research team will set up physiological monitoring including ECG and pulse oximetry for continuous recording of baseline cardiorespiratory stability. Electroencephalography (EEG) electrodes will then be sited to allow continuous monitoring of baseline brain activity for at least 10 min prior to the clinical procedure. A control heel lance will then be performed followed by the clinical heel lance.

The control heel lance is a non-noxious sham procedure whereby the lancet is placed against the participant’s foot rotated at 90°, preventing release of the blade into the foot. This procedure simulates the tactile and auditory aspects of the blood sampling experience without the noxious input. Brain activity, physiology and facial expression (video) will be recorded for both the control heel lance and clinical heel lance to allow assessment of outcome measures including noxious-evoked brain activity, PIPP-R scores, tachycardia and respiratory dynamics (table 1). The heel lance and control stimulus will be linked electronically to the recording equipment as described in previous studies,^7 27 28^ providing precise timing of when the heel lance occurs. In the event of the neonate requiring multiple heel lances, data will only be included from the first heel lance (conditional on data quality). Video recording of the face will commence approximately 30 s prior to the control heel lance and end at least 30 s after the clinical heel lance to allow PIPP-R scoring. For neonates randomised to receive preprocedural stroking, the parent will be instructed to begin stroking down the infant’s leg immediately prior to the clinical heel lance, with the aid of an animated visual cue to help maintain a velocity of 3 cm/s and a duration of 10 s. After the heel lance, blood collection will be delayed for 30 s to allow PIPP-R scoring. For neonates randomised to receive postprocedural stroking, the parent will be instructed to begin stroking down the infant’s leg after the start of blood collection, when deemed appropriate by the clinician performing the heel lance in order to ensure that blood collection is not disrupted. Parents will be guided by an animated visual cue. A researcher will then verbally administer the STAI-S and four-point distress questionnaire after the procedure is completed. Physiological monitoring will continue for 30 min and EEG monitoring for at least 10 min to allow investigation of postprocedural cardiorespiratory dynamics and brain activity as exploratory outcomes of the trial. Finally, the parents will be invited to complete an anonymous survey of their experience and views on research after completion of the study. This study protocol follows the Standard Protocol Items: Recommendations for Interventional Trials guidelines (online supplemental file 2).^57^

10.1136/bmjopen-2022-061841.supp2 Supplementary data

### Intervention

The parental touch intervention will involve one parent stroking the infant’s leg for 10 s. The duration of the intervention is consistent with previous studies.^30 31 52 58^ A member of the research team will inform parents of their randomised allocation (either stroking pre heel lance or post heel lance) at the start of the test occasion. They will explain and demonstrate how to administer the intervention using their whole hand, stroking in one direction down towards the foot. The infant will lay in a cot during the intervention and procedure. During the demonstration and test occasion, PsychoPy software^59^ will be used to provide a visual cue on a computer screen to guide a consistent stroking speed of 3 cm/s for 10 s. During the study, all neonates will receive comfort care in accordance with the local practice guidelines. These measures include swaddling the infants and providing non-nutritive sucking.

### Recording techniques

#### Electroencephalography (EEG)

Electrophysiological activity will be acquired with the SynAmps RT 64-channel headbox and amplifiers (Compumedics Neuroscan) or with the Compumedics Grael V2 EEG system, with a bandwidth from DC: 400 Hz and a sampling rate of 2000 or 2048 Hz. Data recorded at 2048 Hz will be downsampled to 2000 Hz prior to further processing. CURRYscan7 or CURRYscan8 neuroimaging suite (Compumedics Neuroscan) will be used to record the activity. All equipment will conform to the electrical safety standard for medical devices, IEC 60601-1. Eight EEG recording electrodes will be positioned on the scalp at Cz, CPz, C3, C4, FCz, T3, T4 and Oz according to the modified international 10–20 System. Reference and ground electrodes will be placed at Fz and Fpz, respectively. EEG conductive paste will be used to optimise contact with the scalp. All impedances will be reduced to approximately 5 kΩ by rubbing the skin with EEG preparation gel prior to electrode placement. An ECG electrode will be placed on the left clavicle to record heart rate.

#### Physiological monitoring (ECG and pulse oximetry)

Heart rate, respiratory rate and oxygen saturation will be recorded continuously throughout the study period (approximately 1 hour) using ECG and pulse oximetry. Heart rate and oxygen saturation data will be used to calculate the clinical pain scores following the heel lance and control stimulus and to assess clinical stability across the test occasion.

#### Video recording

Video recording will be used to measure behavioural responses that is, changes in facial expression during the control stimulus and clinically required heel lance. A synchronised LED flash will be activated by the researcher simultaneously with each stimulation as a marker for the time of stimulation.

#### Parental questionnaire

The parent administering the intervention will be asked to complete a short series of validated electronic questionnaires assessing anxiety and distress at the start and end of the test occasion (table 2). The researcher will record the responses to the STAI-T, STAI-S and distress questionnaire in an electronic Case Report Form. The electronic device will then be presented to the parent to independently complete a short survey about trial participation and their research experience. The survey will be completed anonymously, and responses will be stored by trial arm with no link to study IDs.

**Table 2:** Trial parental questionnaires

Questionnaire section	Topic	Timing of administration	Questionnaire administrator
20-point State-Trait Anxiety Inventory (STAI)-T	Trait anxiety	Start of test occasion	Administered verbally by researcher
20-point State-Trait Anxiety Inventory (STAI)-S	State anxiety pre heel lance State anxiety post heel lance	Start of test occasion After the procedure and intervention are completed	Administered verbally by researcher Administered verbally by researcher
Four-point distress questionnaire	Emotional constructs experienced at time of the clinical heel lance	After the procedure and intervention are completed	Administered verbally by researcher
Anonymous survey	Views on the trial and infant research	End of test occasion	Completed by parent

#### State-Trait Anxiety Inventory (STAI)

The State-Trait Anxiety Inventory (STAI) is the gold standard assessment for state anxiety.^60^ It is well validated, publicly available and has a trait (STAI-T) version consisting of 20 statements exploring general feelings of anxiety, and a state version (STAI-S) consisting of 20 statements exploring anxiety levels at a particular point in time. Each question is rated on a four-point scale. The range of possible scores for the STAI varies from a minimum score of 20 to a maximum score of 80 on both the STAI-T and STAI-S subscales.

#### Four-point distress questionnaire

Parents will be asked four questions related to their emotions during the clinical heel lance procedure.^61 62^ Each of the four emotional constructs (worried, upset, anxious and sad) will be rated on an 11-point scale ranging from ‘not at all’ (0) to ‘extremely’ (10). A total score between 0 and 40 will be calculated, where higher scores are indicative of greater parental distress. This score is frequently used in research to evaluate parent/child interactions during painful procedures.^61–63^

### Outcome measures

#### Noxious-evoked brain activity

An EEG template that reflects the noxious-evoked brain activity in neonates has previously been defined using principal component analysis, validated in independent data sets^29^ and used in clinical studies and a clinical trial.^32^ This template will be projected onto the EEG data recorded in the 1000 ms period following each heel lance and control heel lance stimulus and the relative weight of the component calculated for each neonate. A greater weight indicates a stronger noxious-evoked response. While the brain activity characterised is directly related to noxious input, it does not reflect all noxious-evoked activity across the brain or all aspects of the pain experience. The response to the non-noxious control heel lance stimulus is being recorded to confirm that it significantly differs from the brain activity evoked by a noxious heel lance. This forms an important data quality control check.^27^

#### PIPP-R score

Clinical pain scores will be evaluated using the validated Premature Infant Pain Profile-Revised,^26^ which is a composite multimodal measure encompassing behavioural, physiological and contextual indicators of the pain response. It allows for different aspects of the infant pain experience to be captured and has been widely used as the primary outcome measure for infant pain in many clinical trials.^64–66^ The PIPP-R score will be calculated for the control heel lance and the clinical heel lance procedure. Heart rate, oxygen saturation and facial expression will be recorded in the 15 s period before and 30 s period after each of the procedures.^25 26^ The 15 s period before the heel lance will be recorded immediately prior to the stroking intervention. Videos of the infant’s facial expressions will be scored offline using the PIPP-R facial coding system. Changes in heart rate and oxygen saturation will be recorded with ECG and pulse oximeter and used to calculate the PIPP-R score. For each participant, PIPP-R scores will be assessed by investigators blinded to the study arm. A second investigator (blinded to the trial arm) will recalculate 20% of the PIPP-R scores to measure inter-rater reliability.

#### Clinical stability

Clinical stability will be assessed in the 30 min periods before and after the heel lance. The percentage of neonates who develop postprocedural tachycardia in the 30 s postheel lance will be a secondary outcome measure of the trial. Tachycardia will be defined as a heart rate >160 beats per minute as per Advanced Paediatric Life Support guidelines, reflecting heart rate values >90th centile for newborn infants in the first week of life.^67 68^ Exploratory outcome measures will also include the time taken for the heart rate to return to baseline values post heel lance and respiratory rate variability in the 30 min prior and post heel lance (including incidence of apnoea). An episode of apnoea will be defined as the cessation of breathing for at least 20 s.^69^

#### Parental experience

Parental anxiety will be quantified using the outcomes of the STAI-T and STAI-S questionnaires. Parental distress will be quantified using the four-point distress score. The anonymous parent survey will assess the parental experience of the trial and parental views on taking part in the trial.

### Statistics and analysis

#### Analysis of outcome measures

Data preprocessing and statistical analysis will be performed blind to treatment allocation. The analysis and presentation of results will follow the most up-to-date recommendations of the Consolidated Standards of Reporting Trials group (CONSORT).^70^ All comparative analyses will be performed using MatlabR2020a or an updated version. The primary results will be presented unadjusted. To perform sensitivity analysis, the minimisation variables will be used to make statistical adjustments to the primary analysis and the sensitivity analysis results will be presented as secondary results. A full statistical analysis plan will be finalised before any comparative analysis of outcome measures is performed.

#### Significance levels

For the analysis of the primary outcome measure, a p-value of 0.05 (two-sided 5% significance level) will be used to indicate statistical significance. Significance levels for secondary outcomes (excluding the sensitivity analysis) will be corrected for multiple comparisons and the method will be specified in the analysis plan. Two-sided statistical tests and corresponding p-values will be presented throughout; however, for the purposes of interpretation of results, CIs will dominate, rather than p-values.

#### Primary

##### Noxious-evoked brain activity

The magnitude of noxious-evoked brain activity will be compared between the two groups using a parametric two-sample t-test if the residuals are normally distributed. If the residuals are non-normally distributed, a Wilcoxon rank-sum test will be used. If appropriate, and depending on the distribution of residuals and the test used, the mean and SD or the median and IQR (or entire range, whichever is appropriate) will be presented for each group and the unadjusted mean or median difference between groups with a 95% CI.

#### Secondary

##### PIPP-R score

PIPP-R scores (during the 30 s period after heel lance) in the two groups will be compared using a two-sample t-test if the residuals are normally distributed. If the residuals are non-normally distributed, a Wilcoxon rank-sum test will be used. If appropriate, and depending on the distribution of residuals and the test used, the mean and SD or the median and IQR (or entire range, whichever is appropriate) will be presented for each group and the unadjusted mean or median difference between groups with a 95% CI.

##### Clinical stability (tachycardia)

The tachycardia outcome per infant will be dichotomous (i.e. 'yes/no' per infant). The percentage of infants experiencing tachycardia will be compared between the two groups using a logistic regression. We will report the proportion of tachycardia for each group as well as the difference in proportions between groups.

##### Parental anxiety

The difference in STAI-S scores before and after the heel lance will be compared between the two groups using a two-sample t-test if the residuals are normally distributed. If the residuals are non-normally distributed, a Wilcoxon rank-sum test will be used. If appropriate, and depending on the distribution of residuals and the test used, the mean and SD or the median and IQR (or entire range, whichever is appropriate) will be presented for each group and the unadjusted mean or median difference between groups with a 95% CI.

#### Exploratory

Exploratory analyses will be conducted to investigate (i) the effects of parental touch on background brain activity, (ii) whether preprocedural parental touch reduces the duration of time for heart rate to return to baseline, (iii) the effect of preprocedural parental touch on respiratory rate variability, respiratory dynamics and the incidence of apnoea and (iv) the parental experience of the procedure and involvement in research.

### Sample size determination

#### Power calculation

The assumptions for these calculations are based on data from mechanistic studies investigating the effect of (experimenter-led) soft brushing of the skin at CT-optimal rate on the response to an experimental noxious stimulus or clinical heel lance in term neonates.^30 31^ The mean (SD) brain activity evoked by heel lancing in the control group is estimated to be 1.07 (0.66). A 40% reduction in the intervention group is considered to be clinically significant and realistic from other studies.^30 31 53^ With 90% power and a two-sided 5% significance level, to observe a 40% reduction in brain activity with a two-sample t-test, a sample size of 102 would be required. Allowing for 10% loss, due to technical difficulties or other clinical issues, this increases to 112.

#### Missing data

Missing data may occur in our trial due to equipment failure, EEG artefacts or clinical issues resulting in withdrawal post randomisation. If missing data exists, we expect it will occur at random, and collected data will be representative of the population. To account for potential missing data, we have inflated our sample size by 10%. The analysis will be conducted using the available data.

### Ethics and dissemination

The trial has been approved by the London South East Research Ethics Committee (ref: 21/LO/0523) and will be conducted in accordance with Good Clinical Practice and the Declaration of Helsinki. EEG is a safe tool used routinely in clinical practice and research to measure brain activity. Surface electrodes are used and temporarily fixed without glue. All heel lances performed during the trial will have been requested by the clinical team responsible for the infant’s medical care. No extra blood tests or noxious procedures will be performed for the purpose of the study. Every effort will be made to minimise inconvenience and prevent disruption of clinical care. There are no expected serious adverse events (SAE) for this trial. Any SAEs identified will be reported to the CI within 24 hours and they will report any unexpected SAEs deemed related to the trial to the REC and Sponsor in accordance with REC/HRA guidance.

Parent(s) may withdraw their neonate from the trial at any time and they are not obliged to give a reason. If parents choose to withdraw their child after the study has begun, they will be asked whether data already collected may be retained and used for the purposes of the trial. Parents will be made aware that this decision has no impact on any aspects of their infant’s continuing care. The attending clinician may also withdraw the neonate from the trial if they consider this to be in their best interest. If any of the exclusion criteria manifest prior to data collection, the participant will be withdrawn.

The results of the study will be disseminated to the scientific and wider community through peer-reviewed publications and national and international meetings and conferences, via the charities Supporting the Sick Newborn And their Parents (SSNAP) and Bliss, and through the National Health S clinical guideline adoption process.

### Patient and public involvement (PPI)

A PPI representative will be included in the extended PMG group and invited to join specific PMG meetings to discuss trial progress and developments. Bliss: for babies born premature or sick is a national UK neonatal charity, which is partly funding the trial. They will receive regular trial progress reports and promote the trial across their various channels, and disseminate the results. The research team will also work closely with the onsite local Oxford charity SSNAP during the design, conduct and dissemination of the trial. SSNAP have reviewed all parent-facing materials, will review manuscripts reporting results and will be involved in disseminating results to the public.

## Discussion

All newborn infants are exposed to clinically necessary painful procedures. Even healthy neonates on postnatal wards can require repeated painful procedures beyond routine Newborn Screening, such as blood tests for glucose monitoring or jaundice, which can be distressing for both neonates and their parents. In the UK, more than 100 000 newborn infants receive neonatal care every year as a result of prematurity or illness,^71^ which, for some, can entail weeks to months of hospitalisation and procedures. As such, improving the management of pain is recognised as a top neonatal UK research priority^72^ and a major concern among parents and neonatal nurses.^73^

Poor management of neonatal pain can have a significant negative impact on parents. Mothers of hospitalised infants report feeling emotionally and psychologically traumatised due to having to allow their infants to undergo clinically necessary painful procedures, and due to feelings of helplessness from being unable to protect or comfort their child.^3–5^ Actively involving parents in care relieves parental distress^74^ and increases the likelihood that infants receive treatment for pain.^1 5 75^ Infant massage, a tactile comfort measure which involves patterns of stroking, has been shown to improve mother–infant bonding and improve postnatal depression,^76^ a condition afflicting at least one in ten UK mothers in the first-year postpartum.^77^ Furthermore, maternal stroking of infants in general has been shown to moderate the behavioural and physiological effects of maternal depression on infants.^78^ Promoting the natural tactile behaviour of stroking to provide evidence-based pain-relief would therefore be beneficial to both mothers and infants.

Anxiety about pain is increasingly recognised as a key factor in parental refusal for procedures such as vitamin-K intramuscular injections at birth^79^ and immunisations.^80–82^ Avoidance of key interventions in early life could have drastic consequences for child health and this issue must be addressed. Indeed, parental anxiety and attitudes during painful procedures can also impact neonatal distress and subsequent pain experience during clinical procedures in later life.^83^ Parental anxiety regarding pain could be alleviated by empowering parents to provide safe and effective pain relief for their child. Unlike other non-pharmacological interventions, this strategy could be broadly implemented regardless of feeding status of the infant or availability of a product like sucrose, in hospital as well as the community, and across high and low resource clinical settings.

CT-fibres likely provide the neurobiological mechanism underlying the benefits of tactile stimulation in early life. Studies have revealed that mothers instinctively stroke their infants at a CT-optimal rate^54 55^ and that this tactile stimulation is beneficial. CT-optimal touch significantly decreases resting heart rates in infants aged 1–4 months^84^ and 9 months,^58^ as well as in premature infants (28–36 weeks’ gestation).^85^ Recent studies have also investigated the neurological correlates of CT-optimal touch in early life. In 2-month-old infants, CT-optimal touch produces greater activation of the insular cortex compared with CT non-optimal touch.^84^ Similarly, in term infants CT-optimal stroking with a soft brush produces activation of the primary somatosensory and posterior insular cortices,^86^ suggesting that the neonatal brain is sensitive to the somatosensory and socio-affective effects of CT-optimal stroking.

The Petal trial is based on clear mechanistic evidence from preliminary cohort studies and is, as such, adequately powered to address the clinical question. It employs a range of multimodal outcomes, including electrophysiological, behavioural and cardiorespiratory measures, to cover the many aspects of pain experience, and seeks to investigate the benefits of the intervention to both neonates and their parents. Blinding of outcome assessment is being performed to ensure the integrity of the trial as it is not possible to blind the researchers at the time of study due to the nature of the intervention. Although parents instinctively stroke at the optimal velocity to stimulate CT-fibres,^54 55^ consistency of the intervention is standardised across the trial by providing an animated visual aid for parents to follow. In the event of a positive trial outcome, the intervention could next be translated to more premature infants and other minor painful skin-breaking procedures performed frequently in infants such as immunisation and cannulation and could be performed by parents or healthcare workers in the absence of parents. The Petal trial investigates a simple, free, low-risk, non-pharmacological pain-relieving intervention, which could be rapidly incorporated into routine clinical practice, benefiting infants, their parents and the wider community.

### Trial status

Participant recruitment is currently ongoing. Protocol version no. 3.0 (date of submission: 3 February 2022).

## Supplementary Material

Reviewer comments

Author's manuscript

## Data Availability

Data sharing not applicable as no data sets generated and/or analysed for this study. Not applicable.

## References

[1] Carbajal R, Rousset A, Danan C, et al. Epidemiology and treatment of painful procedures in neonates in intensive care units. JAMA 2008;300:60–70. 10.1001/jama.300.1.60 18594041

[2] Walker SM. Long-Term effects of neonatal pain. Semin Fetal Neonatal Med 2019;24:101005. 10.1016/j.siny.2019.04.005 30987942

[3] Franck LS, Allen A, Cox S, et al. Parents' views about infant pain in neonatal intensive care. Clin J Pain 2005;21:133–9. 10.1097/00002508-200503000-00004 15722806

[4] Gale G, Franck LS, Kools S, et al. Parents' perceptions of their infant's pain experience in the NICU. Int J Nurs Stud 2004;41:51–8. 10.1016/s0020-7489(03)00096-8 14670394

[5] Kyololo O'Brien M, Stevens BJ, Songok J. Mothers' perceptions about pain in hospitalized newborn infants in Kenya. J Pediatr Nurs 2019;47:51–7. 10.1016/j.pedn.2019.04.015 31039509

[6] Stevens B, Yamada J, Lee GY, et al. Sucrose for analgesia in newborn infants undergoing painful procedures. Cochrane Database Syst Rev 2013:CD001069. 10.1002/14651858.CD001069.pub4 23440783

[7] Slater R, Cornelissen L, Fabrizi L, et al. Oral sucrose as an analgesic drug for procedural pain in newborn infants: a randomised controlled trial. Lancet 2010;376:1225–32. 10.1016/S0140-6736(10)61303-7 20817247 PMC2958259

[8] Tremblay S, Ranger M, Chau CMY, et al. Repeated exposure to sucrose for procedural pain in mouse pups leads to long-term widespread brain alterations. Pain 2017;158:1586–98. 10.1097/j.pain.0000000000000961 28715355 PMC5539923

[9] Schneider J, Duerden EG, Guo T, et al. Procedural pain and oral glucose in preterm neonates: brain development and sex-specific effects. Pain 2018;159:515–25. 10.1097/j.pain.0000000000001123 29200180

[10] Ranger M, Tremblay S, Chau CMY, et al. Adverse behavioral changes in adult mice following neonatal repeated exposure to pain and sucrose. Front Psychol 2018;9:2394. 10.3389/fpsyg.2018.02394 30719013 PMC6348336

[11] Benoit B, Martin-Misener R, Latimer M, et al. Breast-Feeding analgesia in infants: an update on the current state of evidence. J Perinat Neonatal Nurs 2017;31:145–59. 10.1097/JPN.0000000000000253 28437305

[12] Meek J, Huertas A. Cochrane review: non-nutritive sucking, kangaroo care and swaddling/facilitated tucking are observed to reduce procedural pain in infants and young children. Evid Based Nurs 2012;15:84–5. 10.1136/ebnurs-2011-100453 22411160

[13] Johnston C, Campbell-Yeo M, Disher T, et al. Skin-to-skin care for procedural pain in neonates. Cochrane Database Syst Rev 2017;2:CD008435. 10.1002/14651858.CD008435.pub3 28205208 PMC6464258

[14] Jain S, Kumar P, McMillan DD. Prior leg massage decreases pain responses to heel stick in preterm babies. J Paediatr Child Health 2006;42:505–8. 10.1111/j.1440-1754.2006.00912.x 16925535

[15] Bellieni CV, Cordelli DM, Marchi S, et al. Sensorial saturation for neonatal analgesia. Clin J Pain 2007;23:219–21. 10.1097/AJP.0b013e31802e3bd7 17314580

[16] Diego MA, Field T, Hernandez-Reif M. Procedural pain heart rate responses in massaged preterm infants. Infant Behav Dev 2009;32:226–9. 10.1016/j.infbeh.2008.12.001 19185352 PMC2694508

[17] Abdallah B, Badr LK, Hawwari M. The efficacy of massage on short and long term outcomes in preterm infants. Infant Behav Dev 2013;36:662–9. 10.1016/j.infbeh.2013.06.009 23932956

[18] Hathaway EE, Luberto CM, Bogenschutz LH, et al. Integrative care therapies and physiological and pain-related outcomes in hospitalized infants. Glob Adv Health Med 2015;4:32–7. 10.7453/gahmj.2015.029 PMC4533649 26331102

[19] Chik Y-M, Ip W-Y, Choi K-C. The Effect of Upper Limb Massage on Infants’ Venipuncture Pain. Pain Management Nursing 2017;18:50–7. 10.1016/j.pmn.2016.10.001 27964912

[20] Zargham-Boroujeni A, Elsagh A, Mohammadizadeh M. The effects of massage and breastfeeding on response to venipuncture pain among hospitalized neonates. Iran J Nurs Midwifery Res 2017;22:308–12. 10.4103/ijnmr.IJNMR_119_13 28904545 PMC5590362

[21] Fatollahzade M, Parvizi S, Kashaki M, et al. The effect of gentle human touch during endotracheal suctioning on procedural pain response in preterm infant admitted to neonatal intensive care units: a randomized controlled crossover study. J Matern Fetal Neonatal Med 2022;35:1370–6. 10.1080/14767058.2020.1755649 32316790

[22] Field T, Hernandez-Reif M, Feijo L, et al. Prenatal, perinatal and neonatal stimulation: a survey of neonatal nurseries. Infant Behav Dev 2006;29:24–31. 10.1016/j.infbeh.2005.07.006 17138258

[23] Losacco V, Cuttini M, Greisen G, et al. Heel blood sampling in European neonatal intensive care units: compliance with pain management guidelines. Arch Dis Child Fetal Neonatal Ed 2011;96:F65–8. 10.1136/adc.2010.186429 21177753

[24] Courtois E, Droutman S, Magny J-F, et al. Epidemiology and neonatal pain management of heelsticks in intensive care units: EPIPPAIN 2, a prospective observational study. Int J Nurs Stud 2016;59:79–88. 10.1016/j.ijnurstu.2016.03.014 27222453

[25] Stevens B, Johnston C, Petryshen P, et al. Premature infant pain profile: development and initial validation. Clin J Pain 1996;12:13–22. 10.1097/00002508-199603000-00004 8722730

[26] Stevens BJ, Gibbins S, Yamada J, et al. The premature infant pain profile-revised (PIPP-R): initial validation and feasibility. Clin J Pain 2014;30:238–43. 10.1097/AJP.0b013e3182906aed 24503979

[27] Slater R, Worley A, Fabrizi L, et al. Evoked potentials generated by noxious stimulation in the human infant brain. Eur J Pain 2010;14:321–6. 10.1016/j.ejpain.2009.05.005 19481484

[28] Worley A, Fabrizi L, Boyd S, et al. Multi-Modal pain measurements in infants. J Neurosci Methods 2012;205:252–7. 10.1016/j.jneumeth.2012.01.009 22285660 PMC3465552

[29] Hartley C, Duff EP, Green G, et al. Nociceptive brain activity as a measure of analgesic efficacy in infants. Sci Transl Med 2017;9. 10.1126/scitranslmed.aah6122. [Epub ahead of print: 03 05 2017]. PMC5884430 28469039

[30] Gursul D, Goksan S, Hartley C, et al. Stroking modulates noxious-evoked brain activity in human infants. Curr Biol 2018;28:R1380–1. 10.1016/j.cub.2018.11.014 30562526 PMC6303187

[31] Cobo MM, Hartley C, Gursul D. Quantifying individual noxious-evoked baseline sensitivity to optimise analgesic trials in neonates. Elife 2021;10. 10.7554/eLife.65266 PMC8087440 33847561

[32] Hartley C, Moultrie F, Hoskin A, et al. Analgesic efficacy and safety of morphine in the procedural pain in premature infants (Poppi) study: randomised placebo-controlled trial. Lancet 2018;392:2595–605. 10.1016/S0140-6736(18)31813-0 30509743 PMC6294828

[33] Slater R, Fabrizi L, Worley A, et al. Premature infants display increased noxious-evoked neuronal activity in the brain compared to healthy age-matched term-born infants. Neuroimage 2010;52:583–9. 10.1016/j.neuroimage.2010.04.253 20438855

[34] Hertenstein MJ, Verkamp JM, Kerestes AM, et al. The communicative functions of touch in humans, nonhuman primates, and rats: a review and synthesis of the empirical research. Genet Soc Gen Psychol Monogr 2006;132:5–94. 10.3200/mono.132.1.5-94 17345871

[35] Fitri SYR, Nasution SK, Nurhidayah I, et al. Massage therapy as a non-pharmacological analgesia for procedural pain in neonates: a scoping review. Complement Ther Med 2021;59:102735. 10.1016/j.ctim.2021.102735 33974990

[36] Mrljak R, Arnsteg Danielsson A, Hedov G, et al. Effects of infant massage: a systematic review. Int J Environ Res Public Health 2022;19. 10.3390/ijerph19116378. [Epub ahead of print: 24 05 2022]. PMC9179989 35681968

[37] Vallbo A, Olausson H, Wessberg J, et al. A system of unmyelinated afferents for innocuous mechanoreception in the human skin. Brain Res 1993;628:301–4. 10.1016/0006-8993(93)90968-s 8313159

[38] Vallbo AB, Olausson H, Wessberg J, et al. Receptive field characteristics of tactile units with myelinated afferents in hairy skin of human subjects. J Physiol 1995;483:783–95. 10.1113/jphysiol.1995.sp020622 7776258 PMC1157818

[39] Liu Q, Vrontou S, Rice FL, et al. Molecular genetic visualization of a rare subset of unmyelinated sensory neurons that may detect gentle touch. Nat Neurosci 2007;10:946–8. 10.1038/nn1937 17618277

[40] Olausson H, Lamarre Y, Backlund H, et al. Unmyelinated tactile afferents signal touch and project to insular cortex. Nat Neurosci 2002;5:900–4. 10.1038/nn896 12145636

[41] Morrison I, Björnsdotter M, Olausson H. Vicarious responses to social touch in posterior insular cortex are tuned to pleasant caressing speeds. J Neurosci 2011;31:9554–62. 10.1523/JNEUROSCI.0397-11.2011 21715620 PMC6623148

[42] McGlone F, Olausson H, Boyle JA, et al. Touching and feeling: differences in pleasant touch processing between glabrous and hairy skin in humans. Eur J Neurosci 2012;35:1782–8. 10.1111/j.1460-9568.2012.08092.x 22594914

[43] Bennett RH, Bolling DZ, Anderson LC, et al. fNIRS detects temporal lobe response to affective touch. Soc Cogn Affect Neurosci 2014;9:470–6. 10.1093/scan/nst008 23327935 PMC3989128

[44] Gordon I, Voos AC, Bennett RH, et al. Brain mechanisms for processing affective touch. Hum Brain Mapp 2013;34:914–22. 10.1002/hbm.21480 22125232 PMC6869848

[45] McGlone F, Wessberg J, Olausson H. Discriminative and affective touch: sensing and feeling. Neuron 2014;82:737–55. 10.1016/j.neuron.2014.05.001 24853935

[46] von Mohr M, Kirsch LP, Fotopoulou A. The soothing function of touch: affective touch reduces feelings of social exclusion. Sci Rep 2017;7:13516. 10.1038/s41598-017-13355-7 29044137 PMC5647341

[47] Olausson H, Wessberg J, Morrison I, et al. The neurophysiology of unmyelinated tactile afferents. Neurosci Biobehav Rev 2010;34:185–91. 10.1016/j.neubiorev.2008.09.011 18952123

[48] Morrison I, Löken LS, Minde J, et al. Reduced C-afferent fibre density affects perceived pleasantness and empathy for touch. Brain 2011;134:1116–26. 10.1093/brain/awr011 21378097

[49] Löken LS, Wessberg J, Morrison I, et al. Coding of pleasant touch by unmyelinated afferents in humans. Nat Neurosci 2009;12:547–8. 10.1038/nn.2312 19363489

[50] Essick GK, McGlone F, Dancer C, et al. Quantitative assessment of pleasant touch. Neurosci Biobehav Rev 2010;34:192–203. 10.1016/j.neubiorev.2009.02.003 19896001

[51] Triscoli C, Ackerley R, Sailer U. Touch satiety: differential effects of stroking velocity on liking and wanting touch over repetitions. PLoS One 2014;9:e113425. 10.1371/journal.pone.0113425 25405620 PMC4236195

[52] Liljencrantz J, Strigo I, Ellingsen DM, et al. Slow brushing reduces heat pain in humans. Eur J Pain 2017;21:1173–85. 10.1002/ejp.1018 28263013

[53] von Mohr M, Krahé C, Beck B, et al. The social buffering of pain by affective touch: a laser-evoked potential study in romantic couples. Soc Cogn Affect Neurosci 2018;13:1121–30. 10.1093/scan/nsy085 30247679 PMC6234321

[54] Croy I, Luong A, Triscoli C, et al. Interpersonal stroking touch is targeted to C tactile afferent activation. Behav Brain Res 2016;297:37–40. 10.1016/j.bbr.2015.09.038 26433145

[55] Van Puyvelde M, Gorissen A-S, Pattyn N, et al. Does touch matter? The impact of stroking versus non-stroking maternal touch on cardio-respiratory processes in mothers and infants. Physiol Behav 2019;207:55–63. 10.1016/j.physbeh.2019.04.024 31047950

[56] Ackerley R, Backlund Wasling H, Liljencrantz J, et al. Human C-tactile afferents are tuned to the temperature of a skin-stroking caress. J Neurosci 2014;34:2879–83. 10.1523/JNEUROSCI.2847-13.2014 24553929 PMC3931502

[57] Chan A-W, Tetzlaff JM, Altman DG, et al. SPIRIT 2013 statement: defining standard protocol items for clinical trials. Ann Intern Med 2013;158:200–7. 10.7326/0003-4819-158-3-201302050-00583 23295957 PMC5114123

[58] Fairhurst MT, Löken L, Grossmann T. Physiological and behavioral responses reveal 9-month-old infants' sensitivity to pleasant touch. Psychol Sci 2014;25:1124–31. 10.1177/0956797614527114 24681587 PMC4017181

[59] Peirce J, Gray JR, Simpson S, et al. PsychoPy2: experiments in behavior made easy. Behav Res Methods 2019;51:195–203. 10.3758/s13428-018-01193-y 30734206 PMC6420413

[60] Spiegelberger CD, Gorsuch RL, Lushene R. Manual for the State-Trait anxiety inventory. Palo Alto, CA: Consulting Psychologists Press, 1983.

[61] Caes L, Vervoort T, Eccleston C, et al. Parental catastrophizing about child's pain and its relationship with activity restriction: the mediating role of parental distress. Pain 2011;152:212–22. 10.1016/j.pain.2010.10.037 21126822

[62] Vervoort T, Karos K, Johnson D, et al. Parental emotion and pain control behaviour when faced with child's pain: the emotion regulatory role of parental pain-related attention-set shifting and heart rate variability. Pain 2019;160:322–33. 10.1097/j.pain.0000000000001402 30247182

[63] Vervoort T, Trost Z, Sütterlin S, et al. Emotion regulatory function of parent attention to child pain and associated implications for parental pain control behaviour. Pain 2014;155:1453-1463. 10.1016/j.pain.2014.04.015 24769189

[64] Simonse E, Mulder PGH, van Beek RHT. Analgesic effect of breast milk versus sucrose for analgesia during heel lance in late preterm infants. Pediatrics 2012;129:657–63. 10.1542/peds.2011-2173 22392168

[65] Campbell-Yeo ML, Johnston CC, Joseph KS, et al. Cobedding and recovery time after heel lance in preterm twins: results of a randomized trial. Pediatrics 2012;130:500–6. 10.1542/peds.2012-0010 22926182

[66] Taddio A, Shah V, Hancock R, et al. Effectiveness of sucrose analgesia in newborns undergoing painful medical procedures. CMAJ 2008;179:37–43. 10.1503/cmaj.071734 18591525 PMC2464480

[67] Schwartz PJ, Garson A, Paul T, et al. Guidelines for the interpretation of the neonatal electrocardiogram. A task force of the European Society of cardiology. Eur Heart J 2002;23:1329–44. 10.1053/euhj.2002.3274 12269267

[68] Fleming S, Thompson M, Stevens R, et al. Normal ranges of heart rate and respiratory rate in children from birth to 18 years of age: a systematic review of observational studies. Lancet 2011;377:1011–8. 10.1016/S0140-6736(10)62226-X 21411136 PMC3789232

[69] Adjei T, Purdy R, Jorge J. A new method to measure inter-breath intervals in infants for the assessment of apnoea and respiratory dynamics. medRxiv 2021. 10.1136/bmjresp-2021-001042 PMC8666899 34893521

[70] Schulz KF, Altman DG, Moher D, et al. Consort 2010 statement: updated guidelines for reporting parallel group randomised trials. BMJ 2010;340:c332. 10.1136/bmj.c332 20332509 PMC2844940

[71] The National Institute for Health and Care Excellence (NICE). Impact maternity and neonatal care 2019.

[72] Duley L, Uhm S, Oliver S, et al. Top 15 UK research priorities for preterm birth. Lancet 2014;383:2041–2. 10.1016/S0140-6736(14)60989-2 24931684

[73] Wielenga JM, Tume LN, Latour JM, et al. European neonatal intensive care nursing research priorities: an e-Delphi study. Arch Dis Child Fetal Neonatal Ed 2015;100:F66–71. 10.1136/archdischild-2014-306858 25260359

[74] Franck LS, Oulton K, Bruce E. Parental involvement in neonatal pain management: an empirical and conceptual update. J Nurs Scholarsh 2012;44:45–54. 10.1111/j.1547-5069.2011.01434.x 22339845

[75] Johnston C, Barrington KJ, Taddio A, et al. Pain in Canadian NICUs: have we improved over the past 12 years? Clin J Pain 2011;27:225–32. 10.1097/AJP.0b013e3181fe14cf 21178602

[76] Onozawa K, Glover V, Adams D, et al. Infant massage improves mother-infant interaction for mothers with postnatal depression. J Affect Disord 2001;63:201–7. 10.1016/s0165-0327(00)00198-1 11246096

[77] Petersen I, Peltola T, Kaski S, et al. Depression, depressive symptoms and treatments in women who have recently given birth: UK cohort study. BMJ Open 2018;8:e022152. 10.1136/bmjopen-2018-022152 PMC6224756 30361401

[78] Sharp H, Pickles A, Meaney M, et al. Frequency of infant stroking reported by mothers moderates the effect of prenatal depression on infant behavioural and physiological outcomes. PLoS One 2012;7:e45446. 10.1371/journal.pone.0045446 23091594 PMC3473033

[79] Nicholls SG, Southern KW. Parental decision-making and acceptance of newborn bloodspot screening: an exploratory study. PLoS One 2013;8:e79441. 10.1371/journal.pone.0079441 24265771 PMC3827133

[80] Harrington PM, Woodman C, Shannon WF. Low immunisation uptake: is the process the problem? J Epidemiol Community Health 2000;54:394–400. 10.1136/jech.54.5.394 10814662 PMC1731666

[81] Mills E, Jadad AR, Ross C, et al. Systematic review of qualitative studies exploring parental beliefs and attitudes toward childhood vaccination identifies common barriers to vaccination. J Clin Epidemiol 2005;58:1081–8. 10.1016/j.jclinepi.2005.09.002 16223649

[82] Tickner S, Leman PJ, Woodcock A. Parents' views about pre-school immunization: an interview study in southern England. Child Care Health Dev 2010;36:190–7. 10.1111/j.1365-2214.2009.01020.x 19961504

[83] Racine NM, Pillai Riddell RR, Flora DB, et al. Predicting preschool pain-related anticipatory distress: the relative contribution of longitudinal and concurrent factors. Pain 2016;157:1918–32. 10.1097/j.pain.0000000000000590 27276117

[84] Jönsson EH, Kotilahti K, Heiskala J, et al. Affective and non-affective touch evoke differential brain responses in 2-month-old infants. Neuroimage 2018;169:162–71. 10.1016/j.neuroimage.2017.12.024 29242105

[85] Manzotti A, Cerritelli F, Esteves JE, et al. Dynamic touch reduces physiological arousal in preterm infants: a role for c-tactile afferents? Dev Cogn Neurosci 2019;39:100703. 10.1016/j.dcn.2019.100703 31487608 PMC6969366

[86] Tuulari JJ, Scheinin NM, Lehtola S, et al. Neural correlates of gentle skin stroking in early infancy. Dev Cogn Neurosci 2019;35:36–41. 10.1016/j.dcn.2017.10.004 29241822 PMC6968958

