# Supplementary material for: Multicentre, randomised controlled trial to investigate the effects of parental touch on relieving acute procedural pain in neonates (Petal)
Source: BMJ Open. 2022 Jul 19;12(7):e061841. doi: 10.1136/bmjopen-2022-061841 (PMC9301810; doi:10.1136/bmjopen-2022-061841)
Supplement: Supplementary data [file bmjopen-2022-061841supp001.pdf]

Petal

Parental touch trial

Parent Information Leaflet

John Radcliffe Hospital

University of Oxford
Department of Paediatrics, Medical Sciences Division
Oxford University Hospitals NHS Foundation Trust

Cobo MM, *et al. BMJ Open* 2022; 12:e061841. doi: 10.1136/bmjopen-2022-061841

You and your child are eligible to take part in a research study. Before you decide, it is important that you understand why the research is being done and what it involves. Please read the following information and ask us if anything is unclear or if you would like more information.

Study title: **P**ar**e**n**tal** touch trial (*Petal*)
A randomised controlled trial to investigate the effects of parental touch on relieving acute procedural pain in neonates

1. What is the purpose of the study?

Babies in hospital often require clinical procedures as part of their routine medical treatment. As babies cannot tell us how much these procedures hurt, it is difficult to make sure that they are receiving the right pain-relief treatments. We know babies can experience discomfort and pain, and we have developed a method to measure changes in brain activity that occur when a baby undergoes a clinical procedure. We also know that babies display specific facial expressions when they are in pain and that their heart rate and breathing rate can increase.

Touch is important for parent and child bonding, and research in adults has shown that stroking the skin at the right speed can reduce pain experienced during some procedures. Stroking and parental touch activates special fibres in the skin that we think can make procedures feel less painful. Some studies have shown that close skin-to-skin contact between babies and their parents can reduce pain during procedures (such as blood tests).

The aim of this research is to understand if parental touch can reduce how much pain their babies experience during a blood test. We also want to know how parents feel when they stroke their baby's leg during a blood test. We would like to see if there are any differences in how a baby responds if a parent strokes their child's leg before or after a blood test.

2. Why have I been invited?

You and your child have been invited to take part in this study because your child requires a blood test for clinical purposes. We are recruiting parents and their children who were born at least 35 weeks gestation, who need to have a clinical blood test. We hope to recruit in total 112 babies.

3. Do we have to take part?

No, it is your decision whether or not you and your child take part. If you decide to take part, you will be asked to sign a consent form. If you decide you do not want to take part, this will not affect your child's care.

If you decide you would like to take part, you can change your mind at any time and withdraw you and your child from the study by telling the research team. You do not have to give a reason. You will be asked if we can use the data/images that have already been collected for analysis and if we can publish the anonymised results.

4. What is involved in the study?

We would like to understand how parental touch (in the form of stroking) may affect how babies respond to a clinically-required blood test. **No blood tests will be carried out solely for research purposes.** We will only study your child during a blood test that is needed for clinical purposes. Blood tests will be scheduled according to clinical need and thus you may have less than 24 hours to consider your participation in this trial. Blood tests will be completed in the routine way. On some occasions more than one heel lance is required to collect a sufficient blood sample. If this is the case, the research monitoring equipment will not be removed from your baby between heel lances in order to ensure that we do not interfere with the clinical procedures. As part of the study, we will also administer a 'sham' heel lance: this is not a real blood test and will not pierce your baby's skin or cause any pain.

This is a control stimulus to simulate the blood test without the 'painful' part. The heel lance is placed against your baby's foot but angled away so that the sharp fires into the air rather than the foot. The study will not interfere with your child's clinical care, nor will there be any delay if an emergency procedure is required.

As part of the study, we will ask you to stroke your child's leg for 10 seconds before, or 10 seconds after, their blood test. Half of the babies in the study will be stroked by a parent before the blood test, and half will be stroked afterwards. Before the study, your baby's details will be entered into a computer programme that will randomly select whether you should stroke your baby before or after the blood test. Where required, we will demonstrate on a doll the speed, location and duration of the stroking. Before we start the study we will make sure you know how to complete the stroking in the correct way. As part of the study, we will also ask you to complete a short questionnaire; the first part will be before the study, the second part at the end. The questionnaire overall should take approximately 15 minutes.

We will assess your child's responses to the blood test by measuring their brain activity. We will also video your child's face, and measure other responses such heart rate, breathing rate and oxygen saturations. We will monitor your child before, during and after the blood test for approximately one hour.

We will use the following recording measures for your child:

Measuring brain activity
Electroencephalography (EEG)**:** EEG is a portable imaging system to measure brain activity. It involves gently placing electrodes (small discs) on the head using a paste that can be washed off with soap and water. EEG is routinely used on the neonatal unit, children's wards and clinics.

Measuring other responses
Vital sign monitoring: Small adhesive discs will be placed on your child's chest to measure changes in breathing rate and heart rate (this is called an ECG).

Videoing your child: We will also video your child during the study. This is so that we can assess changes in facial expression and body movements, and record the exact timing of the blood test.

We may ask if you are happy for us to use these images for teaching, publicity and/or in scientific journals. If you agree, we will take separate consent for this as your child's face would be present in the video footage. This is an optional part of the study and is not essential. If we do not use the images, this will not affect your child's care or stop your child participating in this research.

5. Are there any additional risks or benefits for my child?
Recording a video of your child is non-invasive and does not present any risk. EEG and ECG have been used clinically for over 20 years without any adverse effects. All studies have a dedicated team of healthcare professionals and researchers that will ensure the safety of your child at all times. We are not aware of any risks for your child taking part in this study.

The research data collected will not be routinely reviewed by a doctor. If any clinically significant findings are identified at the time of the study then the research team will report these to the clinical care team for further review.

There are no direct benefits of taking part in the study. This study is designed to gather information, to help improve the care we provide for babies in the future. If your child becomes distressed, the research study will be paused or stopped. Any clinically required procedures will still go ahead if the clinician looking after your child feels that this is appropriate.

6. What information will be collected about me and my child?

We will collect relevant medical (e.g. age), environmental (e.g. medical ward where study was conducted), demographic (e.g. post code) and social (e.g. ethnicity) information about your child from their medical notes. This information helps us to determine which factors may influence the way a baby copes with pain. We will also collect vital sign data (such as heart rate and breathing rate), recordings of their facial expressions and body movements and changes in brain activity caused by the blood test. We will ask you to complete a questionnaire.

All information and videos that are collected during this research study will be stored confidentially. Each baby will be allocated a study number which will be used to label the data. This study forms part of an educational programme.

7. What will happen to the results?

Results will be analysed and published in a journal. All publications will be made available on our website **https://neuroimaging.paediatrics.ox.ac.uk**. The findings may also be used for teaching or academic research presentations. No identifying information will be presented about you or your child, unless you have provided specific consent for us to use videos or images of your child in this way.

8. What will happen to my data and my child's data?

We will use the information about you and your child in order to conduct this study. Research is a task that we perform in the public interest. The University of Oxford, as Sponsor, is the data controller. This means that we, as University of Oxford researchers and collaborators, are responsible for looking after the information collected and using it properly. We will use the minimum personally-identifiable information possible. We will keep identifiable information about you and your child for up to 12 months after the study has finished. This excludes any research documents with personal information, such as consent forms and facial expression recordings, which will be held securely at the University of Oxford for 21 years after the end of the study.

Data protection regulation provides you with control over your personal data and how it is used. When you agree to your information being used in research, however, some of those rights may be limited in order for the research to be reliable and accurate. Further information about your rights with respect to your personal data is available at http://www.admin.ox.ac.uk/councilsec/compliance/gdpr/individualrights/. You can find out more about how we use your information from the contacts in section 12.

Research data may be shared with other researchers, both here and abroad. Responsible members of the University of Oxford and Oxford University Hospitals NHS Trust may be given access to data for monitoring and/or audit of the study to ensure we are complying with regulations.

9. Who is organising and funding this research?

This study is sponsored by the University of Oxford and has been funded by the Wellcome Trust and the charity BLISS. Your doctor will not be paid for including you in this study.

10. Who has reviewed the study?

All research in the NHS is looked at by an independent group of people, called a Research Ethics Committee, to protect participants' interests. This study has been reviewed and given favourable opinion by the London - South East Research Ethics Committee.

11. Comments or concerns during the study

The University has arrangements in place to provide for harm arising from participation in the study for which the University is the Research Sponsor. NHS indemnity operates in respect of the clinical treatment with which your child is provided. If you wish to complain about any aspect of the way in

which you have been approached or treated during the course of this study, you should contact Prof Rebeccah Slater (details below) or the University of Oxford Clinical Trials and Research Governance (CTRG) office (tel: 01865 616480, email: ctrg@admin.ox.ac.uk).

The Patient Advisory Liaison Service (PALS) is a confidential NHS service that can provide you with support for any complaints or queries you may have regarding the care you receive as an NHS patient. PALS is unable to provide information about this research study. The John Radcliffe Hospital PALS team can be contacted on: Tel: 01865 221473, Email: PALS@ouh.nhs.uk, http://www.ouh.nhs.uk/patient-guide/pals.aspx.

12. Participation in future research

As we are interested in how your child's response to pain changes as they grow, we may ask if we can contact you in the future, to ask if you would be happy for your child to take part in other similar research studies run by our research team. If you agree that we can contact you about other research studies we will ask you to complete an optional additional consent item on the Consent Form used when you agree for your child to participate in the study. We will record your contact details, and these will be kept in a separate electronic database from the rest of the research data. This database is password-protected and can only be accessed by members of the research team.

Your contact details will not be passed onto anyone outside of the research team. All contact will come from the research team in the first instance. You can opt-out of this at any point by contacting Prof Rebeccah Slater (details below). Your agreement for us to contact you does not form any obligation to participate in future research.

What will happen to my data?

If you have provided optional additional consent to be contacted about future studies, we will store your contact details indefinitely unless you choose to opt-out at any point.

13. Contact for further information

Chief Investigator: Dr Eleri Adams eleri.adams@ouh.nhs.uk 01865 221356
Principal Investigator: Prof Rebeccah Slater rebeccah.slater@paediatrics.ox.ac.uk 01865 234229

You can also access further information about research or parent support from the following groups:

- BLISS: UK based charity https://www.bliss.org.uk/
- SSNAP (Support for the Sick Newborn And their Parents): Oxford based charity https://www.ssnap.org.uk

Representative image removed for publication

Picture shows example of an EEG study.

Thank you for reading this leaflet.

Cobo MM, *et al. BMJ Open* 2022; 12:e061841. doi: 10.1136/bmjopen-2022-061841

Petal

Parental touch trial

Parent Information Leaflet

Royal Devon and Exeter Hospital

University of Oxford

Department of Paediatrics
Medical Sciences Division

NHS
Royal Devon University Healthcare
NHS Foundation Trust

Cobo MM, *et al. BMJ Open* 2022; 12:e061841. doi: 10.1136/bmjopen-2022-061841

You and your child are eligible to take part in a research study. Before you decide, it is important that you understand why the research is being done and what it involves. Please read the following information and ask us if anything is unclear or if you would like more information.

Study title: **P**ar**e**n**tal** touch trial (*Petal*)
A randomised controlled trial to investigate the effects of parental touch on relieving acute procedural pain in neonates

1. What is the purpose of the study?

Babies in hospital often require clinical procedures as part of their routine medical treatment. As babies cannot tell us how much these procedures hurt, it is difficult to make sure that they are receiving the right pain-relief treatments. We know babies can experience discomfort and pain, and we have developed a method to measure changes in brain activity that occur when a baby undergoes a clinical procedure. We also know that babies display specific facial expressions when they are in pain and that their heart rate and breathing rate can increase.

Touch is important for parent and child bonding, and research in adults has shown that stroking the skin at the right speed can reduce pain experienced during some procedures. Stroking and parental touch activates special fibres in the skin that we think can make procedures feel less painful. Some studies have shown that close skin-to-skin contact between babies and their parents can reduce pain during procedures (such as blood tests).

The aim of this research is to understand if parental touch can reduce how much pain their babies experience during a blood test. We also want to know how parents feel when they stroke their baby's leg during a blood test. We would like to see if there are any differences in how a baby responds if a parent strokes their child's leg before or after a blood test.

2. Why have I been invited?

You and your child have been invited to take part in this study because your child requires a blood test for clinical purposes. We are recruiting parents and their children who were born at least 35 weeks gestation, who need to have a clinical blood test. We hope to recruit in total 112 babies.

3. Do we have to take part?

No, it is your decision whether or not you and your child take part. If you decide to take part, you will be asked to sign a consent form. If you decide you do not want to take part, this will not affect your child's care.

If you decide you would like to take part, you can change your mind at any time and withdraw you and your child from the study by telling the research team. You do not have to give a reason. You will be asked if we can use the data/images that have already been collected for analysis and if we can publish the anonymised results.

4. What is involved in the study?

We would like to understand how parental touch (in the form of stroking) may affect how babies respond to a clinically-required blood test. **No blood tests will be carried out solely for research purposes.** We will only study your child during a blood test that is needed for clinical purposes. Blood tests will be scheduled according to clinical need and thus you may have less than 24 hours to consider your participation in this trial. Blood tests will be completed in the routine way. On some occasions more than one heel lance is required to collect a sufficient blood sample. If this is the case, the research monitoring equipment will not be removed from your baby between heel lances in order to ensure that we do not interfere with the clinical procedures. As part of the study, we will also administer a 'sham' heel lance: this is not a real blood test and will not pierce your baby's skin

Cobo MM, *et al. BMJ Open* 2022; 12:e061841. doi: 10.1136/bmjopen-2022-061841

or cause any pain. This is a control stimulus to simulate the blood test without the 'painful' part. The heel lance is placed against your baby's foot but angled away so that the sharp fires into the air rather than the foot. The study will not interfere with your child's clinical care, nor will there be any delay if an emergency procedure is required.

As part of the study, we will ask you to stroke your child's leg for 10 seconds before, or 10 seconds after, their blood test. Half of the babies in the study will be stroked by a parent before the blood test, and half will be stroked afterwards. Before the study, your baby's details will be entered into a computer programme that will randomly select whether you should stroke your baby before or after the blood test. Where required we will demonstrate on a doll the speed, location and duration of the stroking. Before we start the study we will make sure you know how to complete the stroking in the correct way. As part of the study, we will also ask you to complete a short questionnaire; the first part will be before the study, the second part at the end. The questionnaire overall should take approximately 15 minutes.

We will assess your child's responses to the blood test by measuring their brain activity. We will also video your child's face, and measure other responses such heart rate, breathing rate and oxygen saturations. We will monitor your child before, during and after the blood test for approximately one hour.

We will use the following recording measures for your child:

Measuring brain activity
Electroencephalography (EEG)**:** EEG is a portable imaging system to measure brain activity. It involves gently placing electrodes (small discs) on the head using a paste that can be washed off with soap and water. EEG is routinely used on the neonatal unit, children's wards and clinics.

Measuring other responses
Vital sign monitoring: Small adhesive discs will be placed on your child's chest to measure changes in breathing rate and heart rate (this is called an ECG).

Videoing your child: We will also video your child during the study. This is so that we can assess changes in facial expression and body movements, and record the exact timing of the blood test.

We may ask if you are happy for us to use these images for teaching, publicity and/or in scientific journals. If you agree, we will take separate consent for this as your child's face would be present in the video footage. This is an optional part of the study and is not essential. If we do not use the images, this will not affect your child's care or stop your child participating in this research.

5. Are there any additional risks or benefits for my child?
Recording a video of your child is non-invasive and does not present any risk. EEG and ECG have been used clinically for over 20 years without any adverse effects. All studies have a dedicated team of healthcare professionals and researchers that will ensure the safety of your child at all times. We are not aware of any risks for your child taking part in this study.

The research data collected will not be routinely reviewed by a doctor. If any clinically significant findings are identified at the time of the study then the research team will report these to the clinical care team for further review.

There are no direct benefits of taking part in the study. This study is designed to gather information, to help improve the care we provide for babies in the future. If your child becomes distressed, the research study will be paused or stopped. Any clinically required procedures will still go ahead if the clinician looking after your child feels that this is appropriate.

Cobo MM, *et al. BMJ Open* 2022; 12:e061841. doi: 10.1136/bmjopen-2022-061841

6. What information will be collected about me and my child?

We will collect relevant medical (e.g. age), environmental (e.g. medical ward where study was conducted), demographic (e.g. post code) and social (e.g. ethnicity) information about your child from their medical notes. This information helps us to determine which factors may influence the way a baby copes with pain. We will also collect vital sign data (such as heart rate and breathing rate), recordings of their facial expressions and body movements and changes in brain activity caused by the blood test. We will ask you to complete a questionnaire.

All information and videos that are collected during this research study will be stored confidentially. Each baby will be allocated a study number which will be used to label the data. This study forms part of an educational programme.

7. What will happen to the results?

Results will be analysed and published in a journal. All publications will be made available on our website **https://neuroimaging.paediatrics.ox.ac.uk**. The findings may also be used for teaching or academic research presentations. No identifying information will be presented about you or your child, unless you have provided specific consent for us to use videos or images of your child in this way.

8. What will happen to my data and my child's data?

We will use the information about you and your child in order to conduct this study. Research is a task that we perform in the public interest. The University of Oxford, as Sponsor, is the data controller. This means that we, as University of Oxford researchers and collaborators, are responsible for looking after the information collected and using it properly. We will use the minimum personally-identifiable information possible. We will keep identifiable information about you and your child for up to 12 months after the study has finished. This excludes any research documents with personal information, such as consent forms and facial expression recordings, which will be held securely at the University of Oxford for 21 years after the end of the study.

Data protection regulation provides you with control over your personal data and how it is used. When you agree to your information being used in research, however, some of those rights may be limited in order for the research to be reliable and accurate. Further information about your rights with respect to your personal data is available at http://www.admin.ox.ac.uk/councilsec/compliance/gdpr/individualrights/. You can find out more about how we use your information from the contacts in section 12.

Research data may be shared with other researchers, both here and abroad. Responsible members of the University of Oxford and Oxford University Hospitals NHS Trust may be given access to data for monitoring and/or audit of the study to ensure we are complying with regulations.

9. Who is organising and funding this research?

This study is sponsored by the University of Oxford and has been funded by the Wellcome Trust and the charity BLISS. Your doctor will not be paid for including you in this study.

10. Who has reviewed the study?

All research in the NHS is looked at by an independent group of people, called a Research Ethics Committee, to protect participants' interests. This study has been reviewed and given favourable opinion by the London - South East Research Ethics Committee.

11. Comments or concerns during the study

The University has arrangements in place to provide for harm arising from participation in the study for which the University is the Research Sponsor. NHS indemnity operates in respect of the clinical

treatment with which your child is provided. If you wish to complain about any aspect of the way in which you have been approached or treated during the course of this study, you should contact Dr Ravi Poorun (details below) or the University of Oxford Clinical Trials and Research Governance (CTRG) office (tel: 01865 616480, email: ctrg@admin.ox.ac.uk).

The Patient Advisory Liaison Service (PALS) is a confidential NHS service that can provide you with support for any complaints or queries you may have regarding the care you receive as an NHS patient. PALS is unable to provide information about this research study. The Royal Devon and Exeter Hospital PALS team can be contacted on: Tel: 01392 402093, Email: rde-tr.PALS@nhs.net, https://www.rdehospital.nhs.uk/patients-visitors/patient-advice-liaison-service-pals/#

12. Participation in future research
As we are interested in how your child's response to pain changes as they grow, we may ask if we can contact you in the future, to ask if you would be happy for your child to take part in other similar research studies run by our research team. If you agree that we can contact you about other research studies we will ask you to complete an optional additional consent item on the Consent Form used when you agree for your child to participate in the study. We will record your contact details, and these will be kept in a separate electronic database from the rest of the research data. This database is password-protected and can only be accessed by members of the research team.

Your contact details will not be passed onto anyone outside of the research team. All contact will come from the research team in the first instance. You can opt-out of this at any point by contacting Prof Rebeccah Slater (details below). Your agreement for us to contact you does not form any obligation to participate in future research.

What will happen to my data?
If you have provided optional additional consent to be contacted about future studies, we will store your contact indefinitely unless you choose to opt-out at any point.

13. Contact for further information
Chief Investigator: Dr Eleri Adams eleri.adams@ouh.nhs.uk 01865 221356
Principal Investigator: Dr Ravi Poorun r.poorun@exeter.ac.uk 01392 406980

You can also access further information about research or parent support from the following groups:

- BLISS: UK based charity https://www.bliss.org.uk/
- SSNAP (Support for the Sick Newborn And their Parents): Oxford based charity https://www.ssnap.org.uk

Bliss

SSNAP
Support for the sick newborn and their parents

Representative image
removed for publication

Picture shows example of an EEG study.

Thank you for reading this leaflet.

Cobo MM, *et al. BMJ Open* 2022; 12:e061841. doi: 10.1136/bmjopen-2022-061841

Consent Form (Oxford)

Petal
Parental touch trial

Study ID: | | | | | | | | | |

Infant's name:

Study Title: **Parental** touch trial (Petal)

Chief Investigator: Dr Eleri Adams **Principal Investigator:** Prof Rebeccah Slater

Please initial each box

Please complete in black ballpoint pen.

1	I confirm that I have read and understood the information sheet (**v. , dated / /)**, for the above study. I have had the opportunity to ask questions and have had these answered satisfactorily.	☐
2	I understand that my participation and my child's participation is voluntary and that me and my child are free to withdraw at any time, without giving any reason, without our medical care or legal rights being affected.	☐
3	I understand that relevant sections of my child's medical notes and data collected during the study may be looked at by individuals from the University of Oxford or Oxford University Hospitals NHS Trust, where it is relevant to my child's taking part in this research. I give permission for these individuals to access to my child's records.	☐
4	I agree to my child being videoed during the study. I understand that recorded images will not be used for public use, only analysis. No identifiable information, including video recordings or imaging, will be used in any publications/presentations. Only anonymised data will be published or presented at meetings.	☐
5	I agree for the collected data to be used for teaching or academic research presentations.	☐
6	I agree to me and my child taking part in the above study.	☐
7	I agree to complete a parental questionnaire related to my child's study.	☐

OPTIONAL

8	I consent to being approached in the future about other similar research studies, by the research team, that my child may be eligible for. I understand that agreeing to be contacted does not oblige me or my child to participate in any further studies.	☐
9	I agree to the images/videos of my child recorded during this study being used for publications and presentations.	☐

Name of parent: ________________

Relationship to baby: ________________

Signature: ________________

Date: ________________

Name of investigator taking consent: ________________

Signature: ________________

Date: ________________

1 to be kept as part of the study documentation (original)
1 copy for parent
1 copy with hospital notes

Parental Touch Trial (Petal) Petal Consent Form (Oxford) v2.0 03-08-2021 CI: Dr Eleri Adams IRAS Ref: 291213 *REC Ref: 21/LO/0523 PI: Prof Rebeccah Slater (Professor of Paediatric Neuroscience) rebeccah.slater@paediatrics.ox.ac.uk 01865 234537*

Consent Form (Exeter)

Petal Parental touch trial

Study ID: | | | | | | | | | |

Infant's name:

Study Title: **Parental** touch trial (Petal)

Chief Investigator: Dr Eleri Adams **Principal Investigator:** Dr Ravi Poorun

Please initial each box

Please complete in black ballpoint pen.

1	I confirm that I have read and understood the information sheet (**v. , dated / / **), for the above study. I have had the opportunity to ask questions and have had these answered satisfactorily.	☐
2	I understand that my participation and my child's participation is voluntary and that me and my child are free to withdraw at any time, without giving any reason, without our medical care or legal rights being affected.	☐
3	I understand that relevant sections of my child's medical notes and data collected during the study may be looked at by individuals from the University of Oxford, Oxford University Hospitals NHS Trust or Royal Devon University Healthcare NHS Foundation Trust, where it is relevant to my child's taking part in this research. I give permission for these individuals to access to my child's records.	☐
4	I agree to my child being videoed during the study. I understand that recorded images will not be used for public use, only analysis. No identifiable information, including video recordings or imaging, will be used in any publications/presentations. Only anonymised data will be published or presented at meetings.	☐
5	I agree for the collected data to be used for teaching or academic research presentations.	☐
6	I agree to me and my child taking part in the above study.	☐
7	I agree to complete a parental questionnaire related to my child's study.	☐

OPTIONAL

8	I consent to being approached in the future about other similar research studies, by the research team, that my child may be eligible for. I understand that agreeing to be contacted does not oblige me or my child to participate in any further studies.	☐
9	I agree to the images/videos of my child recorded during this study being used for publications and presentations.	☐

Name of parent: ____________________

Relationship to baby: ____________________

Signature: ____________________

Date: ____________________

Name of investigator taking consent: ____________________

Signature: ____________________

Date: ____________________

1 to be kept as part of the study documentation (original)
1 copy for parent
1 copy with hospital notes
1 copy with PI

Parental Touch Trial (Petal) Petal Consent Form (Exeter) v2.1 07-06-2022 CI: Dr Eleri Adams IRAS Ref: 291213 REC Ref: 21/LO/0523 PI: Dr Ravi Poorun (NIHR Academic Clinical Fellow (Paediatrics)) r.poorun@exeter.ac.uk 01392 406980
