# Supplementary material for: Multicentre, randomised controlled trial to investigate the effects of parental touch on relieving acute procedural pain in neonates (Petal)
Source: BMJ Open. 2022 Jul 19;12(7):e061841. doi: 10.1136/bmjopen-2022-061841 (PMC9301810; doi:10.1136/bmjopen-2022-061841)
Supplement: Author's manuscript [file bmjopen-2022-061841.draft_revisions.pdf]

BMJ Open is committed to open peer review. As part of this commitment we make the peer review history of every article we publish publicly available.

When an article is published we post the peer reviewers' comments and the authors' responses online. We also post the versions of the paper that were used during peer review. These are the versions that the peer review comments apply to.

The versions of the paper that follow are the versions that were submitted during the peer review process. They are not the versions of record or the final published versions. They should not be cited or distributed as the published version of this manuscript.

BMJ Open is an open access journal and the full, final, typeset and author-corrected version of record of the manuscript is available on our site with no access controls, subscription charges or pay-per-view fees (http://bmjopen.bmj.com).

If you have any questions on BMJ Open's open peer review process please email info.bmjopen@bmj.com

BMJ Open

Study protocol: a multicentre, randomised controlled trial to investigate the effects of parental touch on relieving acute procedural pain in neonates (Petal)

Journal:	*BMJ Open*
Manuscript ID	bmjopen-2022-061841
Article Type:	Protocol
Date Submitted by the Author:	09-Feb-2022

Complete List of Authors:	Cobo, Maria; University of Oxford, Department of Paediatrics; Universidad San Francisco de Quito, Colegio de Ciencias Biologicas y Ambientales<br>Moultrie, Fiona; University of Oxford, Department of Paediatrics<br>Hauck, Annalisa; University of Oxford, Department of Paediatrics<br>Crankshaw, Daniel; University of Oxford, Department of Paediatrics<br>Monk, Vaneesha; University of Oxford, Department of Paediatrics<br>Hartley, Caroline; University of Oxford, Department of Paediatrics<br>Evans Fry, Ria; University of Oxford, Department of Paediatrics<br>Robinson, Shellie; University of Oxford, Department of Paediatrics<br>van der Vaart, Marianne; University of Oxford, Department of Paediatrics<br>Baxter, Luke; University of Oxford, Department of Paediatrics<br>Adams, Eleri; Oxford University Hospitals NHS Foundation Trust, Newborn Care Unit, John Radcliffe Hospital<br>Poorun, Ravi; Royal Devon and Exeter NHS Foundation Trust, Children's Services; University of Exeter, College of Medicine & Health<br>Bhatt , Aomesh ; University of Oxford, Department of Paediatrics<br>Slater, Rebeccah; University of Oxford, Department of Paediatrics

Keywords:	NEONATOLOGY, NEUROPHYSIOLOGY, PAIN MANAGEMENT

SCHOLARONE™
Manuscripts

Study protocol: a multicentre, randomised controlled trial to investigate the effects of parental touch on relieving acute procedural pain in neonates (Petal)

Maria M Cobo[1,2], Fiona Moultrie[1], Annalisa Hauck[1], Daniel Crankshaw[1], Vaneesha Monk[1], Caroline Hartley[1], Ria Evans Fry[1], Shellie Robinson[1], Marianne van der Vaart[1], Luke Baxter[1], Eleri Adams[3], Ravi Poorun[4,5], Aomesh Bhatt[1], Rebeccah Slater[1]*

[1] Department of Paediatrics, University of Oxford, Oxford, UK
[2] Universidad San Francisco de Quito USFQ, Colegio de Ciencias Biologicas y Ambientales, Quito, Ecuador
[3] Newborn Care Unit, John Radcliffe Hospital, Oxford University Hospitals NHS Foundation Trust, Oxford, UK
[4] Children's Services, Royal Devon & Exeter NHS Foundation Trust
[5] College of Medicine & Health, University of Exeter

Corresponding author: Rebeccah Slater; rebeccah.slater@paediatrics.ox.ac.uk

For peer review only

ABSTRACT

Introduction

Newborn infants routinely undergo minor painful procedures as part of postnatal care, with infants born sick or premature requiring a greater number of procedures. As pain in early life can have long-term neurodevelopmental consequences and lead to parental anxiety and future avoidance of interventions, effective pain management is essential. Non-pharmacological comfort measures such as breastfeeding, swaddling, and sweet solutions are inconsistently implemented and are not always practical or effective in reducing the transmission of noxious input to the brain. Stroking of the skin can activate C-tactile fibres and reduce pain, and therefore could provide a simple and safe parent-led intervention for the management of pain. The trial aim is to determine whether parental touch prior to a painful clinical procedure provides effective pain relief in neonates.

Methods and analysis

This is a multicentre blinded randomised controlled trial. A total of 112 neonates born at 35 weeks' gestation or more requiring a blood test in the first week of life will be recruited and randomised to receive parental stroking either pre- or post-procedure. We will monitor brain activity (EEG), cardiac and respiratory dynamics, oxygen saturation and facial expression to provide proxy pain outcome measures. The primary outcome will be the reduction of noxious-evoked brain activity in response to a heel lance. Secondary outcomes will be a reduction in clinical pain scores (Premature Infant Pain Profile-Revised), post-procedural tachycardia and parental anxiety.

Ethics and dissemination

The study has been approved by the London – South East Research Ethics Committee (ref:21/LO/0523). The results will be widely disseminated through peer-reviewed publications, international conferences and via our partner neonatal charities Bliss and SSNAP. If the parental tactile intervention is effective, recommendations will be submitted via the NHS clinical guideline adoption process.

Study Status: Commenced September 2021.

Trial Registration: ClinicalTrials.gov NCT04901611; **ISRCTN**: 14135962.

Strengths and limitations of this study
• Petal will be the first randomised controlled trial to investigate whether noxious-evoked brain activity is reduced by pre-procedural parental stroking.
• The trial is based upon published evidence from two mechanistic studies which show a reduction in noxious-evoked brain activity during a heel lance or experimental stimuli in neonates whose skin was brushed by the experimenter prior to the procedure.
• This trial investigates a simple, free, low-risk, non-pharmacological pain-relieving intervention, which could be delivered by parents in partnership with healthcare professionals globally.
• The trial employs multiple proxy measures of pain to determine the impact of the stroking intervention. In addition, it investigates the impact of the intervention on parental anxiety and distress.
• The nature of the intervention prevents the clinical investigators from being blinded at the time of study, which is a limitation of the trial. To mitigate this concern all investigators involved in all other aspects of the trial, including data analysis, are blinded.

For peer review only - http://bmjopen.bmj.com/site/about/guidelines.xhtml

INTRODUCTION

Background

Newborn infants undergo painful procedures as part of routine neonatal care. Sick or premature infants experience an average of ten painful procedures per day as part of life-sustaining treatment.[1] It is recognised that repetitive exposure to pain in early life can cause short-term physiological instability as well as long-term neurodevelopmental consequences such as reduced growth, altered structural and functional brain development, and reduced school-age academic performance.[2] Furthermore, repeatedly witnessing their infant in pain can have a significant negative impact on the emotional and psychological wellbeing of parents.[3–5] Effective pain management is therefore essential in neonatal care. However, measuring pain in this non-verbal patient population is challenging, and few safe and effective analgesics have been tested and approved for use in infants. Non-pharmacological strategies have been introduced and promoted over the last few decades for the management of acute procedural pain. Sweet-taste solutions such as sucrose are effective in relieving behavioural responses following minor painful procedures [6] but do not reduce noxious input to the brain.[7] This has caused concern that this intervention may not mitigate the long-term consequences of early life pain, and furthermore, it may have long-term neurodevelopmental effects with repeated use.[8–10] Breastfeeding also reduces behavioural and physiological responses to pain in full-term infants undergoing heel lancing, intramuscular injection, and venepuncture.[11] However, this strategy can be challenging for new mothers, is not always practical to implement in premature and critically ill infants or in mothers with transmissible infections, or due to maternal concerns of developing a negative association with breastfeeding.[12] Other comfort measures include swaddling and facilitated tucking of infants, which, although useful, are less effective in reducing pain.[13] While many studies have reported the potential pain-relieving effects of tactile interventions such a skin-to-skin care [14] and massage [15–21] in the context of minor painful procedures, these non-pharmacological interventions are scarcely used in maternity and neonatal units [22,23] and the mechanisms underpinning their effectiveness are still being established. Despite guidelines recommending the use of non-pharmacological interventions for pain relief, uptake of these practices remains poor and inconsistent.[24,25]

Measuring pain in infants

The assessment of pain and analgesia in infants primarily relies upon measuring changes in infant behaviour. One of the most common validated clinical pain tools is the Premature Infant Pain Profile (original PIPP, revised PIPP-R).[26,27] While subjective evaluations of behavioural responses are a gold standard for the clinical assessment of neonatal pain, electrophysiology based methods have more recently been developed to identify a pattern of noxious-evoked brain activity.[28–30] This objective and quantifiable neurophysiological measure has been previously used in pilot studies [31,32] and as the primary outcome measure in randomised clinical trials published in *The Lancet,* assessing the analgesic efficacy of sucrose [7] and morphine [33]. Noxious-evoked brain activity has specifically been well-characterised in response to heel lancing,[28–30,34] a clinical procedure which is frequently performed in neonates for blood collection, and will be used as the primary outcome of the Petal trial to investigate the efficacy of pre-procedural parental stroking.

Rationale

Maternal touch behaviours are instinctive, evolutionarily conserved amongst mammals [35] and enhance infant growth [36] and development [37]. Stroking, by repeatedly applying gentle pressure to the skin, can activate C-tactile (CT) fibres, a subclass of slow-conducting unmyelinated sensory neurons, mostly found in hairy skin [38–40]. These fibres project to brain regions associated with affective processing such as the insular cortex, prefrontal cortex, superior temporal sulcus, and cingulate cortex [41–45] and are thought to have evolved to promote affiliative behaviours and social touch [46–49]. CT fibres are optimally activated by stroking at a velocity of 3cm/s (optimal range 1–10cm/s) [50–52], and studies in adults of gentle brushing or stroking paradigms at this optimal velocity have demonstrated a reduction in pain ratings [53,54] and noxious-evoked brain activity [54]. CT-optimal stimulation therefore could provide a natural and safe pain-relieving intervention.

We previously conducted a small prospective cohort study of pre-procedural stroking for pain relief in neonates, in which we demonstrated that CT-optimal stroking (at 3cm/s) prior to an experimental noxious stimulus or clinical

For peer review only - http://bmjopen.bmj.com/site/about/guidelines.xhtml

heel lance significantly reduced noxious-evoked brain activity in term neonates compared to no touch intervention.[31] We replicated this study in an independent sample of term neonates and showed consistent results and a similar effect size in the group receiving the stroking intervention.[32] However, in both of these studies stroking was delivered by the researcher using a soft experimental brush with a known force. Although the studies did not identify a significant effect of the intervention on a clinical pain score, it was notably not powered to investigate this. Considering CT-optimal stroking is a natural maternal behaviour [55,56] and evidence suggesting that CT fibres respond optimally to touch at human skin temperature,[57] hands-on parental stroking has the potential to provide even greater benefit than CT-optimal brushstrokes. Pilot work further suggests that stroking a neonate has similar efficacy to researcher-led experimental brushing (unpublished).

Aim and Objectives

In the Petal trial, we aim to determine whether parental stroking prior to a common painful clinical procedure (heel lancing) provides effective analgesia in neonates. The primary outcome will be the reduction of noxious-evoked brain activity during a heel lance. Secondary outcomes will be a reduction in clinical pain scores, post-procedural tachycardia and parental anxiety (Table 1). Exploratory outcomes will investigate changes in brain activity during the intervention, as well as effects on physiological recovery post-procedure (using heart rate and respiratory dynamics) and further explore parental anxiety, distress, and attitudes to research.

For peer review only - http://bmjopen.bmj.com/site/about/guidelines.xhtml

Objectives	Outcome Measures
**Primary Objective**<br>(i) To test whether parental touch prior to the clinical procedure reduces noxious-evoked brain activity following a heel lance.	**Primary Outcome Measure**<br>(i) Magnitude of noxious-evoked brain activity following a heel lance (EEG data recorded in the 1000ms period following each heel lance).
**Secondary Objectives**<br>(i) To test whether parental touch prior to the clinical procedure reduces clinical pain scores (PIPP-R) during the 30-second period after the heel lance.	**Secondary Outcome Measure**<br>(i) PIPP-R score during the 30-second period after the heel lance.
(ii) To test whether parental touch prior to the clinical procedure reduces incidence of post-procedural tachycardia activity following a heel lance.	(ii) Percentage of neonates who develop tachycardia in the 30-second post-heel lance.
(iii) To test whether parental touch prior to the clinical procedure reduces parental anxiety, compared with post-procedural touch.	(iii) Difference in STAI-S scores pre- and post-procedure.
**Exploratory Objectives**<br>(i) To explore how parental touch impacts background brain activity.	**Exploratory Outcome Measures**<br>(i) Changes in brain activity during touch intervention.
(ii) To explore whether parental touch prior to the clinical procedure reduces the duration of time for heart rate to return to baseline after a heel lance.	(ii) Time taken for heart rate to return to baseline post-heel lance.
(iii) To explore how parental touch prior to the clinical procedure affects respiratory stability.	(iii) Post-procedural respiratory dynamics and incidence of apnoea.
(iv) To explore parental anxiety and distress, and their experience of the trial and infant research.	(iv) Scores for individual parameters from the STAI-T and STAI-S; 4-point distress questionnaire score; responses to survey about participation in Petal and infant research.

Table 1. Objectives and Outcome Measures. STAI-T: State-Trait Anxiety Inventory-Trait, STAI-S: State-Trait Anxiety Inventory-State, PIPP-R: Premature Infant Pain Profile – Revised.

For peer review only

For peer review only - http://bmjopen.bmj.com/site/about/guidelines.xhtml

METHODS AND ANALYSIS

Trial Description

This is a multicentre randomised-controlled interventional trial, with two research sites (John Radcliffe Hospital, Oxford, and Royal Devon and Exeter Hospital, Devon, UK). The parents of eligible neonates satisfying inclusion criteria (Figure 1) will be approached by a member of the research team. Parental written informed consent will be taken and neonates will be electronically randomised to receive parental stroking either prior to or after a clinically-required heel lance. A unique study ID will be assigned to each individual participant. The randomisation program will use a minimisation algorithm to ensure approximate balance between the groups with respect to gestational age at birth, postnatal age at time of randomisation, sex, the indication for blood sampling, and research site. The users of the system will be blind to the next allocation.

Each neonate will be studied on a single test occasion lasting approximately one hour (Figure 2) and will not require further follow-up. A parent will first complete the State-Trait Anxiety Inventory-Trait (STAI-T) and State-Trait Anxiety Inventory-State (STAI-S) questionnaires, which will be administered verbally by a researcher. This will allow assessment of both trait anxiety and state anxiety prior to the commencement of the stroking intervention or blood test. At least 30 minutes prior to the heel lance, the research team will set up physiological monitoring including electrocardiography (ECG) and pulse oximetry for continuous recording of baseline cardiorespiratory stability. Electroencephalography (EEG) electrodes will then be sited to allow continuous monitoring of baseline brain activity for at least 10 minutes prior to the clinical procedure. A control heel lance will then be performed followed by the clinical heel lance.

The control heel lance is a non-noxious sham procedure whereby the lancet is placed against the participant's foot rotated at 90 degrees, preventing release of the blade into the foot. This procedure simulates the tactile and auditory aspects of the blood sampling experience without the noxious input. Brain activity, physiology and facial expression (video) will be recorded for both the control heel lance and clinical heel lance to allow assessment of outcome measures including noxious-evoked brain activity, PIPP-R scores, tachycardia, and respiratory dynamics (Table 1). The heel lance and control stimulus will be linked electronically to the recording equipment as described in previous studies,[7,28,29] providing precise timing of when the heel lance occurs. In the event of the neonate requiring multiple heel lances, data will only be included from the first heel lance (conditional on data quality). Video monitoring of the face will commence approximately 30 seconds prior to the control lance and end at least 30 seconds after the clinical heel lance to allow PIPP-R scoring. For neonates randomised to receive pre-procedural stroking, the parent will be instructed to begin stroking down the infant's leg immediately prior to the clinical heel lance, with the aid of an animated visual cue to help maintain a velocity of 3cm/s and a duration of 10 seconds. After the heel lance, blood collection will be delayed for 30 seconds to allow PIPP-R scoring. For neonates randomised to receive post-procedural stroking, the parent will be instructed to begin stroking down the infant's leg immediately after blood collection following the animated visual cue. A researcher will then verbally administer the STAI-S and 4-point distress questionnaire after the procedure is completed. Physiological monitoring will continue for 30 minutes and EEG monitoring for at least 10 minutes to allow investigation of post-procedural cardiorespiratory dynamics and brain activity as exploratory outcomes of the trial. Finally, the parents will be invited to complete an anonymous survey of their experience and views on research after completion of the study.

Intervention

The parental touch intervention will involve one parent stroking the infant's leg for 10 seconds. The duration of the intervention is consistent with previous studies.[31,32,53,58] A member of the research team will inform parents of their randomised allocation (either stroking pre-heel lance or post-heel lance) at the start of the test occasion. They will explain and demonstrate how to administer the intervention using their whole hand, stroking in one direction down towards the foot. The infant will lay in a cot during the intervention and procedure. During the demonstration and test occasion, PsychoPy software [59] will be used to provide a visual cue on a computer screen to guide a consistent stroking speed of 3cm/s for 10 seconds.

For peer review only - http://bmjopen.bmj.com/site/about/guidelines.xhtml

Recording Techniques

Electroencephalography (EEG)

Electrophysiological activity will be acquired with the SynAmps RT 64-channel headbox and amplifiers (Compumedics Neuroscan) or with the Compumedics Grael V2 EEG system, with a bandwidth from DC - 400 Hz and a sampling rate of 2000 or 2048Hz. Data recorded at 2048Hz will be downsampled to 2000Hz prior to further processing. CURRYscan7 or CURRYscan8 neuroimaging suite (Compumedics Neuroscan) will be used to record the activity. All equipment will conform to the electrical safety standard for medical devices, IEC 60601-1. Eight EEG recording electrodes will be positioned on the scalp at Cz, CPz, C3, C4, FCz, T3, T4 and Oz according to the modified international 10-20 System. Reference and ground electrodes will be placed at Fz and the forehead respectively. EEG conductive paste will be used to optimise contact with the scalp. All impedances will be reduced to approximately 5kΩ by rubbing the skin with EEG preparation gel prior to electrode placement. An ECG electrode will be placed on the left clavicle to record heart rate.

Physiological Monitoring (ECG and Pulse Oximetry)

Heart rate, respiratory rate and oxygen saturation will be recorded continuously throughout the study period (approximately one hour) using ECG and pulse oximetry. Heart rate and oxygen saturation data will be used to calculate the clinical pain scores following the heel lance and control stimulus and to assess clinical stability across the test occasion.

Video Recording

Video recording will be used to measure behavioural responses i.e. changes in facial expression during the control stimulus and clinically-required heel lance. A synchronised LED flash will be activated by the researcher simultaneously with each stimulation as a marker for the time of stimulation.

Parental Questionnaire

The parent administering the intervention will be asked to complete a short series of validated electronic questionnaires assessing anxiety and distress at the start and end of the test occasion (Table 2). The researcher will record the responses to the STAI-T, STAI-S and distress questionnaire in an electronic Case Report Form. The electronic device will then be presented to the parent to independently complete a short survey about trial participation and their research experience. The survey will be completed anonymously, and responses will be stored by trial arm with no link to study IDs.

Questionnaire section	Topic	Timing of administration	Questionnaire administrator
20-point State-Trait Anxiety Inventory (STAI)-T	Trait anxiety	Start of test occasion	Administered verbally by researcher
20-point State-Trait Anxiety Inventory (STAI)-S	State anxiety pre-heel lance<br><br>State anxiety post-heel lance	Start of test occasion<br><br>After the procedure and intervention are completed	Administered verbally by researcher<br><br>Administered verbally by researcher
4-point distress questionnaire	Emotional constructs experienced at time of the clinical heel lance	After the procedure and intervention are completed	Administered verbally by researcher
Anonymous survey	Views on the trial and infant research	End of test occasion	Completed by parent

Table 2. Trial parental questionnaires.

For peer review only - http://bmjopen.bmj.com/site/about/guidelines.xhtml

State-Trait Anxiety Inventory (STAI)
The State-Trait Anxiety Inventory (STAI) is the gold standard assessment for state anxiety.[60] It is well validated, publicly available and has a trait (STAI-T) version consisting of 20 statements exploring general feelings of anxiety, and a state version (STAI-S) consisting of 20 statements exploring anxiety levels at a particular point in time. Each question is rated on a 4-point scale. The range of possible scores for the STAI varies from a minimum score of 20 to a maximum score of 80 on both the STAI-T and STAI-S subscales. Scores are classified as “no or low anxiety” (20-37), “moderate anxiety” (38-44), and “high anxiety” (45-80).

4-point distress questionnaire
Parents will be asked four questions related to their emotions during the clinical heel lance procedure.[61,62] Each of the four emotional constructs (worried, upset, anxious and sad) will be rated on an 11-point scale ranging from “not at all” (0) to “extremely” (10). A total score between 0 and 40 will be calculated, where higher scores are indicative of greater parental distress. This score is frequently used in research to evaluate parent/child interactions during painful procedures.[61–63]

Outcome Measures
Noxious-evoked Brain Activity
An EEG template that reflects the noxious-evoked brain activity in neonates has previously been defined using principal component analysis, validated in independent data sets [30] and used in clinical studies and a clinical trial.[33] This template will be projected onto the EEG data recorded in the 1000ms period following each heel lance and control heel lance stimulus and the relative weight of the component calculated for each neonate. A greater weight indicates a stronger noxious-evoked response. While the brain activity characterised is directly related to noxious input, it does not reflect all noxious-evoked activity across the brain or all aspects of the pain experience. The response to the non-noxious control heel lance stimulus is being recorded to confirm that it significantly differs from the brain activity evoked by a noxious heel lance. This forms an important data quality control check.[28]

PIPP-R Score
Clinical pain scores will be evaluated using the validated Premature Infant Pain Profile - Revised,[27] which is a composite multimodal measure encompassing behavioural, physiological and contextual indicators of the pain response. It allows for different aspects of the infant pain experience to be captured and has been widely used as the primary outcome measure for infant pain in many clinical trials.[64–66] The PIPP-R score will be calculated for the control heel lance and the clinical heel lance procedure. Heart rate, oxygen saturation and facial expression will be recorded in the 15-second period before and 30-second period after each of the procedures.[26,27] The 15-second period before the heel lance will be recorded immediately prior to the stroking intervention. Videos of the infant’s facial expressions will be scored offline using the PIPP-R facial coding system. Changes in heart rate and oxygen saturation will be recorded with ECG and pulse oximeter and used to calculate the PIPP-R score. A second investigator (blinded to the trial arm) will re-calculate 20% of the PIPP-R scores to measure inter-rater reliability.

Clinical Stability
Clinical stability will be assessed in the 30-minute periods before and after the heel lance. The percentage of neonates who develop post-procedural tachycardia in the 30 seconds post-heel lance will be a secondary outcome measure of the trial. Tachycardia will be defined as a heart rate >160 beats per minute as per Advanced Paediatric Life Support guidelines, reflecting heart rate values >90th centile for newborn infants in the first week of life.[67,68] Exploratory outcome measures will also include the time taken for the heart rate to return to baseline values post-heel lance and respiratory rate variability in the 30 minutes prior and post heel lance (including incidence of apnoea). An episode of apnoea will be defined as the cessation of breathing for at least 20 seconds.[69]

Parental Experience
Parental anxiety will be quantified using the outcomes of the STAI-T and STAI-S questionnaires. Parental distress will be quantified using the 4-point distress score. The anonymous parent survey will assess the parental experience of the trial and parental views on taking part in the trial.

For peer review only - http://bmjopen.bmj.com/site/about/guidelines.xhtml

Statistics and Analysis

Analysis of outcome measures

Data pre-processing and statistical analysis will be performed blind to treatment allocation. The analysis and presentation of results will follow the most up-to-date recommendations of the CONSORT group. All comparative analyses will be performed using MatlabR2020a or an updated version, adjusting for important prognostic factors where possible: these are likely to be a subset of the minimisation factors at randomisation. A full statistical analysis plan will be finalised before any comparative analysis of outcome measures is performed.

Significance Levels

For the analysis of the primary outcome measure, a p-value of 0.05 (two-sided 5% significance level) will be used to indicate statistical significance. Significance levels for secondary outcomes will be corrected for multiple comparisons and the method will be specified in the analysis plan. Two-sided statistical tests and corresponding p-values will be presented throughout; however, for the purposes of interpretation of results, confidence intervals will dominate, rather than p-values.

Primary

Noxious-evoked brain activity

The projected weight of the template will be compared between the two groups using linear regression, dependent on the mean and variance of the data. If appropriate, we will present the mean and standard deviation for each group and estimate the adjusted mean difference with a 95% confidence interval. If the outcome data are skewed, we will present the median and interquartile range (or entire range, whichever is appropriate) for each group, and estimate the median difference between groups with a corresponding 95% confidence interval.

Secondary

PIPP-R score

PIPP-R scores (during the 30-second period after heel lance) in the two groups will be compared using linear regression to estimate the adjusted mean difference. If the PIPP-R scores are skewed, we will present the median and interquartile range (or entire range, whichever is appropriate) for each group, and estimate the median difference between groups.

Clinical stability (tachycardia)

The percentage of infants experiencing tachycardia will be compared between the two groups. Depending on the distribution of these counts, either Poisson or linear regression will be used to calculate the adjusted effect estimates. If appropriate, adjustments for the 30-minute baseline period will also be made. Alternatively, we will present the medians and interquartile ranges (or entire range, whichever is appropriate) for each group and estimate the median differences between groups. In the event of occurrences being very infrequent, logistic regression may be used as an alternative.

Parental anxiety

STAI scores in the two groups will be compared to estimate the adjusted mean difference in pre-procedural and post-procedural STAI-S scores. If the scores are skewed, we will present the median and interquartile range (or entire range, whichever is appropriate) for each group, and estimate the median difference between groups.

Exploratory

Exploratory analyses will be conducted to investigate i) the effects of parental touch on background brain activity, ii) whether pre-procedural parental touch reduces the duration of time for heart rate to return to baseline, iii) the effect of pre-procedural parental touch on respiratory rate variability, respiratory dynamics and the incidence of apnoea and iv) the parental experience of the procedure and involvement in research.

Sample Size Determination

For peer review only - http://bmjopen.bmj.com/site/about/guidelines.xhtml

Power Calculation

The assumptions for these calculations are based on data from mechanistic studies investigating the effect of (experimenter-led) soft brushing of the skin at CT-optimal rate on the response to an experimental noxious stimulus or clinical heel lance in term neonates.[31,32] The mean (SD) brain activity evoked by heel lancing in the control group is estimated to be 1.07 (0.66). A 40% reduction in the intervention group is considered to be clinically significant and realistic from other studies.[31,54] With 90% power and a two-sided 5% significance level, to observe a 40% reduction in brain activity, a sample size of 102 would be required. Allowing for 10% loss, due to technical difficulties or other clinical issues, this increases to 112.

Missing Data

Missing data may occur in our trial due to equipment failure, EEG artefacts, or clinical issues resulting in withdrawal post-randomisation. If missing data exists, we expect it will occur at random, and collected data will be representative of the population. To account for potential missing data, we have inflated our sample size by 10%. The analysis will be conducted using the available data.

Ethics and dissemination

The trial has been approved by the London South East Research Ethics Committee (ref:21/LO/0523) and will be conducted in accordance with Good Clinical Practice and the Declaration of Helsinki. EEG is a safe tool used routinely in clinical practice and research to measure brain activity. Surface electrodes are used and temporarily fixed without glue. All heel lances performed during the trial will have been requested by the clinical team responsible for the infant's medical care. No extra blood tests or noxious procedures will be performed for the purpose of the study. Every effort will be made to minimise inconvenience and prevent disruption of clinical care. There are no expected serious adverse events (SAE) for this trial. Any SAEs identified will be reported to the CI within 24 hours and they will report any unexpected SAEs deemed related to the trial to the REC and Sponsor in accordance with REC/HRA guidance.

Parent(s) may withdraw their neonate from the trial at any time and they are not obliged to give a reason. If parents choose to withdraw their child after the study has begun, they will be asked whether data already collected may be retained and used for the purposes of the trial. Parents will be made aware that this decision has no impact on any aspects of their infant's continuing care. The attending clinician may also withdraw the neonate from the trial if they consider this to be in their best interest. If any of the exclusion criteria manifest prior to data collection, the participant will be withdrawn.

The results of the study will be disseminated to the scientific and wider community through peer-reviewed publications and national and international meetings and conferences, via the charities SSNAP and Bliss, and through the NHS clinical guideline adoption process.

Patient and Public Involvement (PPI)

A PPI representative will be included in the extended PMG group and invited to join specific PMG meetings to discuss trial progress and developments. Bliss: for babies born premature or sick is a national UK neonatal charity, which is partly funding the trial. They will receive regular trial progress reports and promote the trial across their various channels, and disseminate the results. The research team will also work closely with the on-site local Oxford charity Supporting the Sick Newborn And their Parents (SSNAP) during the design, conduct and dissemination of the trial. SSNAP have reviewed all parent-facing materials, will review manuscripts reporting results, and will be involved in disseminating results to the public.

DISCUSSION

All newborn infants are exposed to clinically-necessary painful procedures. Even healthy neonates on postnatal wards can require repeated painful procedures beyond routine Newborn Screening, such as blood tests for glucose

For peer review only - http://bmjopen.bmj.com/site/about/guidelines.xhtml

monitoring or jaundice, which can be distressing for both neonates and their parents. In the UK, more than 100,000 newborn infants receive neonatal care every year as a result of prematurity or illness,[70] which, for some, can entail weeks to months of hospitalisation and procedures. As such, improving the management of pain is recognised as a top neonatal UK research priority [71] and a major concern amongst parents and neonatal nurses.[72]

Poor management of neonatal pain can have a significant negative impact on parents. Mothers of hospitalised infants report feeling emotionally and psychologically traumatised due to having to allow their infants to undergo clinically-necessary painful procedures, and due to feelings of helplessness from being unable to protect or comfort their child.[3–5] Actively involving parents in care relieves parental distress[73] and increases the likelihood that infants receive treatment for pain.[1,5,74] Infant massage, a tactile comfort measure which involves patterns of stroking, has been shown to improve mother-infant bonding and improve postnatal depression,[75] a condition afflicting at least one in ten UK mothers in the first-year post-partum[76]. Furthermore, maternal stroking of infants in general has been shown to moderate the behavioural and physiological effects of maternal depression on infants.[77] Promoting the natural tactile behaviour of stroking to provide evidence-based pain-relief would therefore be beneficial to both mothers and infants.

Anxiety about pain is increasingly recognised as a key factor in parental refusal for procedures such as vitamin-K intramuscular injections at birth[78] and immunisations[79–81]. Avoidance of key interventions in early life could have drastic consequences for child health and this issue must be addressed. Indeed, parental anxiety and attitudes during painful procedures can also impact neonatal distress and subsequent pain experience during clinical procedures in later life[82]. Parental anxiety regarding pain could be alleviated by empowering parents to provide safe and effective pain relief for their child. Unlike other non-pharmacological interventions, this strategy could be broadly implemented regardless of feeding status of the infant or availability of a product like sucrose, in hospital as well as the community, and across high and low resource clinical settings.

CT-fibres likely provide the neurobiological mechanism underlying the benefits of tactile stimulation in early life. Studies have revealed that mothers instinctively stroke their infants at a CT-optimal rate[55,56] and that this tactile stimulation is beneficial. CT-optimal touch significantly decreases resting heart rates in infants aged 1-4 months[58] and 9-months,[83] as well as in premature infants (28–36 weeks' gestation)[84]. Recent studies have also investigated the neurological correlates of CT-optimal touch in early life. In 2-month-old infants, CT-optimal touch produces greater activation of the insular cortex compared to CT non-optimal touch.[84] Similarly, in term infants CT-optimal stroking with a soft brush produces activation of the primary somatosensory and posterior insular cortices,[85] suggesting that the neonatal brain is sensitive to the somatosensory and socio-affective effects of CT-optimal stroking.

The Petal trial is based upon clear mechanistic evidence from preliminary cohort studies and is, as such, adequately powered to address the clinical question. It employs a range of multimodal outcomes, including electrophysiological, behavioural, and cardiorespiratory measures, to cover the many aspects of pain experience, and seeks to investigate the benefits of the intervention to both neonates and their parents. Blinding of outcome assessment is being performed to ensure the integrity of the trial as it is not possible to blind the researchers at the time of study due to the nature of the intervention. Although parents instinctively stroke at the optimal velocity to stimulate CT-fibres,[55,56] consistency of the intervention is standardised across the trial by providing an animated visual aid for parents to follow. In the event of a positive trial outcome, the intervention could next be translated to more premature infants and other minor painful skin-breaking procedures performed frequently in infants such as immunisation and cannulation and could be performed by parents or healthcare workers in the absence of parents. The Petal trial investigates a simple, free, low-risk, non-pharmacological pain-relieving intervention, which could be rapidly incorporated into routine clinical practice, benefiting infants, their parents, and the wider community.

Trial status

Participant recruitment is currently ongoing. Protocol version no. 3.0 (date of submission: 3 February 2022).

For peer review only - http://bmjopen.bmj.com/site/about/guidelines.xhtml

Acknowledgements
The Authors thank the Wellcome trust and Bliss Charity for helping to fund the study, as well as the neonatal team, parents and babies who will help throughout the study.

FOOTNOTES

Author contributions
MC: Conceptualization, Methodology, Software, Investigation, Data Curation, Writing - Original Draft, Writing - Review & Editing, Visualization, Project Administration. **FM:** Conceptualization, Methodology, Investigation, Writing - Original Draft, Writing - Review & Editing, Funding Acquisition. **AH:** Methodology, Investigation, Data Curation, Writing - Review & Editing, Project Administration. **DC:** Methodology, Software, Investigation, Data Curation, Writing - Review & Editing. **VM:** Conceptualization, Methodology, Writing - Original Draft, Project Administration, Funding Acquisition. **CH:** Software, Methodology, Data Curation, Funding Acquisition. **REF:** Conceptualization, Methodology, Investigation, Writing - Original Draft. **SR:** Investigation. **MvdV:** Software, Validation, Data Curation, Writing - Review & Editing. **LB:** Methodology, Data Curation, Writing - Review & Editing. **EA:** Conceptualization, Methodology, Writing - Review & Editing, Supervision. **RP:** Conceptualization, Methodology, Investigation, Data Curation, Supervision, Project Administration. **AB:** Conceptualization, Methodology, Supervision, Writing - Original Draft, Project administration, Funding Acquisition. **RS:** Conceptualization, Methodology, Resources, Writing - Original Draft, Writing - Review & Editing, Supervision, Project Administration, Funding Acquisition.

Funding
This work is supported by The Wellcome Trust via a senior fellowship awarded to Rebeccah Slater (grant number 207457/Z/17/Z) and by Bliss (a UK charity) via a research grant (grant number N/A).

Competing interests
None declared.

Ethics approval
London South East Research Ethics Committee (ref:21/LO/0523).

Figure legend
Figure 1. Trial flowchart. IVH: Intraventricular haemorrhage.
Figure 2. Trial procedures

REFERENCES

1 Carbajal R, Rousset A, Danan C, *et al.* Epidemiology and treatment of painful procedures in neonates in intensive care units. *JAMA* 2008;300:60–70.
2 Walker SM. Long-term effects of neonatal pain. *Semin Fetal Neonatal Med* 2019;24:101005.
3 Franck LS, Allen A, Cox S, *et al.* Parents' views about infant pain in neonatal intensive care. *Clin J Pain* 2005;21:133–9.
4 Gale G, Franck LS, Kools S, *et al.* Parents' perceptions of their infant's pain experience in the NICU. *Int J Nurs Stud* 2004;41:51–8.
5 Kyololo OM, Stevens BJ, Songok J. Mothers' Perceptions about Pain in Hospitalized Newborn Infants in Kenya. *J Pediatr Nurs* 2019;47:51–7.
6 Stevens B, Yamada J, Lee GY, *et al.* Sucrose for analgesia in newborn infants undergoing painful procedures. *Cochrane database Syst Rev* 2013;:CD001069.

For peer review only - http://bmjopen.bmj.com/site/about/guidelines.xhtml

For peer review only

7 Slater R, Cornelissen L, Fabrizi L, *et al.* Oral sucrose as an analgesic drug for procedural pain in newborn infants: a randomised controlled trial. *Lancet* 2010;376:1225–32.

8 Tremblay S, Ranger M, Chau CMY, *et al.* Repeated exposure to sucrose for procedural pain in mouse pups leads to long-term widespread brain alterations. *Pain* 2017;158:1586–98.

9 Schneider J, Duerden EG, Guo T, *et al.* Procedural pain and oral glucose in preterm neonates: brain development and sex-specific effects. *Pain* 2018;159:515–25.

10 Ranger M, Tremblay S, Chau CMY, *et al.* Adverse behavioral changes in adult mice following neonatal repeated exposure to pain and sucrose. *Front Psychol* 2019;9:2394.

11 Benoit B, Martin-Misener R, Latimer M, *et al.* Breast-Feeding Analgesia in Infants: An Update on the Current State of Evidence. *J Perinat Neonatal Nurs* 2017;31:145–59.

12 Tansky C, Lindberg CE. Breastfeeding as a Pain Intervention When Immunizing Infants. *J Nurse Pract* 2010;6:287–95.

13 Meek J, Huertas A. Cochrane review: Non-nutritive sucking, kangaroo care and swaddling/facilitated tucking are observed to reduce procedural pain in infants and young children. *Evid Based Nurs* 2012;15:84–5.

14 Johnston C, Campbell-Yeo M, Disher T, *et al.* Skin-to-skin care for procedural pain in neonates. *Cochrane database Syst Rev* 2017;2:CD008435.

15 Jain S, Kumar P, McMillan DD. Prior leg massage decreases pain responses to heel stick in preterm babies. *J Paediatr Child Health* 2006;42:505–8.

16 Bellieni C V, Cordelli DM, Marchi S, *et al.* Sensorial saturation for neonatal analgesia. *Clin J Pain* 2007;23:219–21.

17 Diego MA, Field T, Hernandez-Reif M. Procedural pain heart rate responses in massaged preterm infants. *Infant Behav Dev* 2009;32:226–9.

18 Abdallah B, Badr LK, Hawwari M. The efficacy of massage on short and long term outcomes in preterm infants. *Infant Behav Dev* 2013;36:662–9.

19 Hathaway EE, Luberto CM, Bogenschutz LH, *et al.* Integrative Care Therapies and Physiological and Pain-related Outcomes in Hospitalized Infants. *Glob Adv Heal Med* 2015;4:32–7.

20 Chik Y-M, Ip W-Y, Choi K-C. The Effect of Upper Limb Massage on Infants' Venipuncture Pain. *Pain Manag Nurs Off J Am Soc Pain Manag Nurses* 2017;18:50–7.

21 Zargham-Boroujeni A, Elsagh A, Mohammadizadeh M. The Effects of Massage and Breastfeeding on Response to Venipuncture Pain among Hospitalized Neonates. *Iran J Nurs Midwifery Res* 2017;22:308–12.

22 Field T, Hernandez-Reif M, Feijo L, *et al.* Prenatal, perinatal and neonatal stimulation: a survey of neonatal nurseries. *Infant Behav Dev* 2006;29:24–31.

23 De Clifford-Faugère G, Aita M, Le May S. Nurses' practices regarding procedural pain management of preterm infants. *Appl Nurs Res* 2019;45:52–4.

24 Losacco V, Cuttini M, Greisen G, *et al.* Heel blood sampling in European neonatal intensive care units: compliance with pain management guidelines. *Arch Dis Child Fetal Neonatal Ed* 2011;96:F65-8.

25 Courtois E, Droutman S, Magny J-F, *et al.* Epidemiology and neonatal pain management of heelsticks in intensive care units: EPIPPAIN 2, a prospective observational study. *Int J Nurs Stud* 2016;59:79–88.

26 Stevens B, Johnston C, Petryshen P, *et al.* Premature Infant Pain Profile: development and initial validation. *Clin J Pain* 1996;12:13–22.

27 Stevens BJ, Gibbins S, Yamada J, *et al.* The premature infant pain profile-revised (PIPP-R): initial validation and feasibility. *Clin J Pain* 2014;30:238–43.

28 Slater R, Worley A, Fabrizi L, *et al.* Evoked potentials generated by noxious stimulation in the human infant brain. *Eur J Pain* 2010;14:321–6.

29 Worley A, Fabrizi L, Boyd S, *et al.* Multi-modal pain measurements in infants. *J Neurosci Methods* 2012;205:252–7.

30 Hartley C, Duff EP, Green G, *et al.* Nociceptive brain activity as a measure of analgesic efficacy in infants. *Sci Transl Med* 2017;9.

31 Gursul D, Goksan S, Hartley C, *et al.* Stroking modulates noxious-evoked brain activity in human infants. Curr. Biol. 2018;28:R1380–1.

32 Cobo MM, Hartley C, Gursul D, *et al.* Quantifying individual noxious-evoked baseline sensitivity to optimise analgesic trials in neonates. *Elife* 2021;10.

For peer review only - http://bmjopen.bmj.com/site/about/guidelines.xhtml

33 Hartley C, Moultrie F, Hoskin A, *et al.* Analgesic efficacy and safety of morphine in the Procedural Pain in Premature Infants (Poppi) study: randomised placebo-controlled trial. *Lancet* 2018;392:2595–605.
34 Slater R, Fabrizi L, Worley A, *et al.* Premature infants display increased noxious-evoked neuronal activity in the brain compared to healthy age-matched term-born infants. *Neuroimage* 2010;52:583–9.
35 Hertenstein MJ, Verkamp JM, Kerestes AM, *et al.* The communicative functions of touch in humans, nonhuman primates, and rats: a review and synthesis of the empirical research. *Genet Soc Gen Psychol Monogr* 2006;132:5–94.
36 Field T, Diego M, Hernandez-Reif M. Potential underlying mechanisms for greater weight gain in massaged preterm infants. *Infant Behav Dev* 2011;34:383–9.
37 Vickers A, Ohlsson A, Lacy JB, *et al.* Massage for promoting growth and development of preterm and/or low birth-weight infants. *Cochrane database Syst Rev* 2004;2004:CD000390.
38 Vallbo A, Olausson H, Wessberg J, *et al.* A system of unmyelinated afferents for innocuous mechanoreception in the human skin. *Brain Res* 1993;628:301–4.
39 Vallbo AB, Olausson H, Wessberg J, *et al.* Receptive field characteristics of tactile units with myelinated afferents in hairy skin of human subjects. *J Physiol* 1995;483 (Pt 3:783–95.
40 Liu Q, Vrontou S, Rice FL, *et al.* Molecular genetic visualization of a rare subset of unmyelinated sensory neurons that may detect gentle touch. *Nat Neurosci* 2007;10:946–8.
41 Olausson H, Lamarre Y, Backlund H, *et al.* Unmyelinated tactile afferents signal touch and project to insular cortex. *Nat Neurosci* 2002;5:900–4.
42 Morrison I, Björnsdotter M, Olausson H. Vicarious responses to social touch in posterior insular cortex are tuned to pleasant caressing speeds. *J Neurosci* 2011;31:9554–62.
43 McGlone F, Olausson H, Boyle JA, *et al.* Touching and feeling: differences in pleasant touch processing between glabrous and hairy skin in humans. *Eur J Neurosci* 2012;35:1782–8.
44 Bennett RH, Bolling DZ, Anderson LC, *et al.* fNIRS detects temporal lobe response to affective touch. *Soc Cogn Affect Neurosci* 2014;9:470–6.
45 Gordon I, Voos AC, Bennett RH, *et al.* Brain mechanisms for processing affective touch. *Hum Brain Mapp* 2013;34:914–22.
46 McGlone F, Wessberg J, Olausson H. Discriminative and affective touch: sensing and feeling. *Neuron* 2014;82:737–55.
47 von Mohr M, Kirsch LP, Fotopoulou A. The soothing function of touch: affective touch reduces feelings of social exclusion. *Sci Rep* 2017;7:13516.
48 Olausson H, Wessberg J, Morrison I, *et al.* The neurophysiology of unmyelinated tactile afferents. *Neurosci Biobehav Rev* 2010;34:185–91.
49 Morrison I, Löken LS, Minde J, *et al.* Reduced C-afferent fibre density affects perceived pleasantness and empathy for touch. *Brain* 2011;134:1116–26.
50 Löken LS, Wessberg J, Morrison I, *et al.* Coding of pleasant touch by unmyelinated afferents in humans. *Nat Neurosci* 2009;12:547–8.
51 Essick GK, McGlone F, Dancer C, *et al.* Quantitative assessment of pleasant touch. *Neurosci Biobehav Rev* 2010;34:192–203.
52 Triscoli C, Ackerley R, Sailer U. Touch satiety: differential effects of stroking velocity on liking and wanting touch over repetitions. *PLoS One* 2014;9:e113425.
53 Liljencrantz J, Strigo I, Ellingsen DM, *et al.* Slow brushing reduces heat pain in humans. *Eur J Pain* 2017;21:1173–85.
54 von Mohr M, Krahé C, Beck B, *et al.* The social buffering of pain by affective touch: a laser-evoked potential study in romantic couples. *Soc Cogn Affect Neurosci* 2018;13:1121–30.
55 Croy I, Luong A, Triscoli C, *et al.* Interpersonal stroking touch is targeted to C tactile afferent activation. *Behav Brain Res* 2016;297:37–40.
56 Van Puyvelde M, Gorissen A-S, Pattyn N, *et al.* Does touch matter? The impact of stroking versus non-stroking maternal touch on cardio-respiratory processes in mothers and infants. *Physiol Behav* 2019;207:55–63.
57 Ackerley R, Backlund Wasling H, Liljencrantz J, *et al.* Human C-tactile afferents are tuned to the temperature of a skin-stroking caress. *J Neurosci* 2014;34:2879–83.
58 Fairhurst MT, Löken L, Grossmann T. Physiological and behavioral responses reveal 9-month-old infants'

For peer review only - http://bmjopen.bmj.com/site/about/guidelines.xhtml

sensitivity to pleasant touch. *Psychol Sci* 2014;25:1124–31.

59 Peirce J, Gray JR, Simpson S, *et al.* PsychoPy2: Experiments in behavior made easy. *Behav Res Methods* 2019;51:195–203.

60 Spiegelberger CD, Gorsuch RL, Lushene R, *et al. Manual for the State-Trait Anxiety Inventory*. Palo Alto, CA: : Consulting Psychologists Press 1983.

61 Caes L, Vervoort T, Eccleston C, *et al.* Parental catastrophizing about child’s pain and its relationship with activity restriction: the mediating role of parental distress. *Pain* 2011;152:212–22.

62 Vervoort T, Karos K, Johnson D, *et al.* Parental emotion and pain control behaviour when faced with child’s pain: the emotion regulatory role of parental pain-related attention-set shifting and heart rate variability. *Pain* 2019;160:322–33.

63 Vervoort T, Trost Z, Sütterlin S, *et al.* Emotion regulatory function of parent attention to child pain and associated implications for parental pain control behaviour. *Pain* 2014;155.

64 Simonse E, Mulder PGH, van Beek RHT. Analgesic effect of breast milk versus sucrose for analgesia during heel lance in late preterm infants. *Pediatrics* 2012;129:657–63.

65 Campbell-Yeo ML, Johnston CC, Joseph KS, *et al.* Cobedding and recovery time after heel lance in preterm twins: results of a randomized trial. *Pediatrics* 2012;130:500–6.

66 Taddio A, Shah V, Hancock R, *et al.* Effectiveness of sucrose analgesia in newborns undergoing painful medical procedures. *C Can Med Assoc J = J l’Association medicale Can* 2008;179:37–43.

67 Schwartz PJ, Garson AJ, Paul T, *et al.* Guidelines for the interpretation of the neonatal electrocardiogram. A task force of the European Society of Cardiology. *Eur Heart J* 2002;23:1329–44.

68 Fleming S, Thompson M, Stevens R, *et al.* Normal ranges of heart rate and respiratory rate in children from birth to 18 years of age: a systematic review of observational studies. *Lancet (London, England)* 2011;377:1011–8.

69 Adjei T, Purdy R, Jorge J, *et al.* A new method to measure inter-breath intervals in infants for the assessment of apnoea and respiratory dynamics. *medRxiv* 2021;:2021.05.29.21258043.

70 NICE. NICEimpact maternity and neonatal care. 2019.

71 Duley L, Uhm S, Oliver S. Top 15 UK research priorities for preterm birth. Lancet (London, England). 2014;383:2041–2.

72 Wielenga JM, Tume LN, Latour JM, *et al.* European neonatal intensive care nursing research priorities: an e-Delphi study. *Arch Dis Child Fetal Neonatal Ed* 2015;100:F66-71.

73 Franck LS, Oulton K, Bruce E. Parental involvement in neonatal pain management: an empirical and conceptual update. *J Nurs Scholarsh an Off Publ Sigma Theta Tau Int Honor Soc Nurs* 2012;44:45–54.

74 Johnston C, Barrington KJ, Taddio A, *et al.* Pain in Canadian NICUs: have we improved over the past 12 years? *Clin J Pain* 2011;27:225–32.

75 Onozawa K, Glover V, Adams D, *et al.* Infant massage improves mother-infant interaction for mothers with postnatal depression. *J Affect Disord* 2001;63:201–7.

76 Petersen I, Peltola T, Kaski S, *et al.* Depression, depressive symptoms and treatments in women who have recently given birth: UK cohort study. *BMJ Open* 2018;8:e022152.

77 Sharp H, Pickles A, Meaney M, *et al.* Frequency of infant stroking reported by mothers moderates the effect of prenatal depression on infant behavioural and physiological outcomes. *PLoS One* 2012;7:e45446.

78 Nicholls SG, Southern KW. Parental decision-making and acceptance of newborn bloodspot screening: an exploratory study. *PLoS One* 2013;8:e79441.

79 Harrington PM, Woodman C, Shannon WF. Low immunisation uptake: is the process the problem? *J Epidemiol Community Health* 2000;54:394–400.

80 Mills E, Jadad AR, Ross C, *et al.* Systematic review of qualitative studies exploring parental beliefs and attitudes toward childhood vaccination identifies common barriers to vaccination. *J Clin Epidemiol* 2005;58:1081–8.

81 Tickner S, Leman PJ, Woodcock A. Parents’ views about pre-school immunization: an interview study in southern England. *Child Care Health Dev* 2010;36:190–7.

82 Racine NM, Pillai Riddell RR, Flora DB, *et al.* Predicting preschool pain-related anticipatory distress: the relative contribution of longitudinal and concurrent factors. *Pain* 2016;157:1918–32.

83 Manzotti A, Cerritelli F, Esteves JE, *et al.* Dynamic touch reduces physiological arousal in preterm infants: A role for c-tactile afferents? *Dev Cogn Neurosci* 2019;39:100703.

For peer review only - http://bmjopen.bmj.com/site/about/guidelines.xhtml

84 Jönsson EH, Kotilahti K, Heiskala J, *et al.* Affective and non-affective touch evoke differential brain responses in 2-month-old infants. *Neuroimage* 2018;169:162–71.

85 Tuulari JJ, Scheinin NM, Lehtola S, *et al.* Neural correlates of gentle skin stroking in early infancy. *Dev Cogn Neurosci* 2019;35:36–41.

For peer review only

For peer review only - http://bmjopen.bmj.com/site/about/guidelines.xhtml

Inclusion Criteria

- Born at the John Radcliffe Hospital, Oxford or the Royal Devon and Exeter Hospital, Devon
- Born at or after 35+0 weeks' gestation with a postnatal age ≤7 days
- Require a clinical heel lance as part of clinical care
- Parents/guardians have given written informed consent for inclusion in the trial.

Exclusion Criteria

- Hypoxic Ischaemic Encephalopathy
- IVH > grade II
- Received any analgesics/sedatives in the last 24 hours
- Born with a congenital malformation or genetic condition known to affect neurological development
- Born to a mother with a history of substance abuse.

Randomisation (1:1 allocation ratio)
Web-based randomisation

30 min baseline data collection
(EEG, heart rate, oxygen saturation)

Group A (n=56)
Parental touch pre-heel lance

Group B (n=56)
Parental touch post-heel lance

Primary Outcome measures

(i) Magnitude of noxious-evoked brain activity evoked by heel lance.

Secondary Outcome measures

(i) Premature Infant Pain Profile – Revised (PIPP-R) score during the 30-second period after the heel lance.
(ii) Percentage of neonates who develop tachycardia in the 30 seconds post-heel lance.
(iii) Parent questionnaire assessing anxiety.

Exploratory Outcome measures

(i) Changes in background brain activity during touch intervention.
(ii) Time taken for heart rate to return to baseline post-heel lance.
(iii) Post procedural respiratory dynamics and incidence of apnoea.
(iv) Parental questionnaire assessing parental anxiety, distress, and experience of research.

For peer review only - http://bmjopen.bmj.com/site/about/guidelines.xhtml

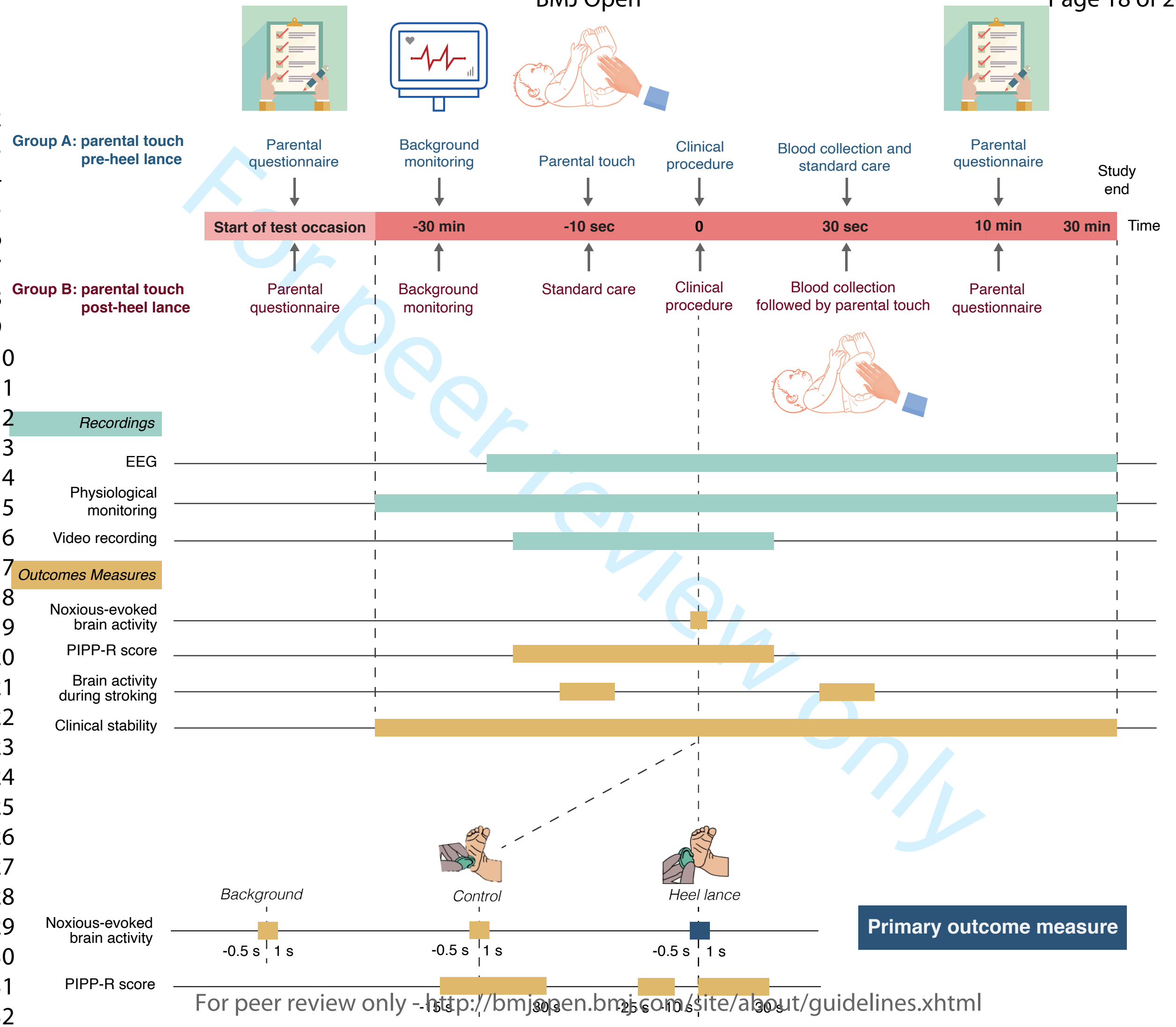

For peer review only

For peer review only - http://bmjopen.bmj.com/site/about/guidelines.xhtml

Study protocol: a multicentre, randomised controlled trial to investigate the effects of parental touch on relieving acute procedural pain in neonates (Petal)

SPIRIT
STANDARD PROTOCOL ITEMS: RECOMMENDATIONS FOR INTERVENTIONAL TRIALS

SPIRIT 2013 Checklist: Recommended items to address in a clinical trial protocol and related documents*

Included in manuscript	Section/item	ItemNo	Description	Comment
	**Administrative information**			
X	Title	1	Descriptive title identifying the study design, population, interventions, and, if applicable, trial acronym	
X	Trial registration	2a	Trial identifier and registry name. If not yet registered, name of intended registry	
		2b	All items from the World Health Organization Trial Registration Data Set	
X	Protocol version	3	Date and version identifier	
X	Funding	4	Sources and types of financial, material, and other support	
X	Roles and responsibilities	5a	Names, affiliations, and roles of protocol contributors	
		5b	Name and contact information for the trial sponsor	
		5c	Role of study sponsor and funders, if any, in study design; collection, management, analysis, and interpretation of data; writing of the report; and the decision to submit the report for publication, including whether they will have ultimate authority over any of these activities	

For peer review only

Study protocol: a multicentre, randomised controlled trial to investigate the effects of parental touch on relieving acute procedural pain in neonates (Petal)

		5d	Composition, roles, and responsibilities of the coordinating centre, steering committee, endpoint adjudication committee, data management team, and other individuals or groups overseeing the trial, if applicable (see Item 21a for data monitoring committee)
	**Introduction**		
X	Background and rationale	6a	Description of research question and justification for undertaking the trial, including summary of relevant studies (published and unpublished) examining benefits and harms for each intervention
		6b	Explanation for choice of comparators
X	Objectives	7	Specific objectives or hypotheses
X	Trial design	8	Description of trial design including type of trial (eg, parallel group, crossover, factorial, single group), allocation ratio, and framework (eg, superiority, equivalence, noninferiority, exploratory)
	**Methods: Participants, interventions, and outcomes**		
X	Study setting	9	Description of study settings (eg, community clinic, academic hospital) and list of countries where data will be collected. Reference to where list of study sites can be obtained
X	Eligibility criteria	10	Inclusion and exclusion criteria for participants. If applicable, eligibility criteria for study centres and individuals who will perform the interventions (eg, surgeons, psychotherapists)
X	Interventions	11a	Interventions for each group with sufficient detail to allow replication, including how and when they will be administered

For peer review only - http://bmjopen.bmj.com/site/about/guidelines.xhtml

Study protocol: a multicentre, randomised controlled trial to investigate the effects of parental touch on relieving acute procedural pain in neonates (Petal)

		11b	Criteria for discontinuing or modifying allocated interventions for a given trial participant (eg, drug dose change in response to harms, participant request, or improving/worsening disease)
		11c	Strategies to improve adherence to intervention protocols, and any procedures for monitoring adherence (eg, drug tablet return, laboratory tests)
		11d	Relevant concomitant care and interventions that are permitted or prohibited during the trial
X	Outcomes	12	Primary, secondary, and other outcomes, including the specific measurement variable (eg, systolic blood pressure), analysis metric (eg, change from baseline, final value, time to event), method of aggregation (eg, median, proportion), and time point for each outcome. Explanation of the clinical relevance of chosen efficacy and harm outcomes is strongly recommended
X	Participant timeline	13	Time schedule of enrolment, interventions (including any run-ins and washouts), assessments, and visits for participants. A schematic diagram is highly recommended (see Figure)
X	Sample size	14	Estimated number of participants needed to achieve study objectives and how it was determined, including clinical and statistical assumptions supporting any sample size calculations
X	Recruitment	15	Strategies for achieving adequate participant enrolment to reach target sample size

Methods: Assignment of interventions (for controlled trials)

Allocation:

Study protocol: a multicentre, randomised controlled trial to investigate the effects of parental touch on relieving acute procedural pain in neonates (Petal)

X	Sequence generation	16a	Method of generating the allocation sequence (eg, computer-generated random numbers), and list of any factors for stratification. To reduce predictability of a random sequence, details of any planned restriction (eg, blocking) should be provided in a separate document that is unavailable to those who enrol participants or assign interventions
	Allocation concealment mechanism	16b	Mechanism of implementing the allocation sequence (eg, central telephone; sequentially numbered, opaque, sealed envelopes), describing any steps to conceal the sequence until interventions are assigned
	Implementation	16c	Who will generate the allocation sequence, who will enrol participants, and who will assign participants to interventions
X	Blinding (masking)	17a	Who will be blinded after assignment to interventions (eg, trial participants, care providers, outcome assessors, data analysts), and how
		17b	If blinded, circumstances under which unblinding is permissible, and procedure for revealing a participant's allocated intervention during the trial
	**Methods: Data collection, management, and analysis**		
X	Data collection methods	18a	Plans for assessment and collection of outcome, baseline, and other trial data, including any related processes to promote data quality (eg, duplicate measurements, training of assessors) and a description of study instruments (eg, questionnaires, laboratory tests) along with their reliability and validity, if known. Reference to where data collection forms can be found, if not in the protocol

Study protocol: a multicentre, randomised controlled trial to investigate the effects of parental touch on relieving acute procedural pain in neonates (Petal)

		18b	Plans to promote participant retention and complete follow-up, including list of any outcome data to be collected for participants who discontinue or deviate from intervention protocols
X	Data management	19	Plans for data entry, coding, security, and storage, including any related processes to promote data quality (eg, double data entry; range checks for data values). Reference to where details of data management procedures can be found, if not in the protocol
X	Statistical methods	20a	Statistical methods for analysing primary and secondary outcomes. Reference to where other details of the statistical analysis plan can be found, if not in the protocol
		20b	Methods for any additional analyses (eg, subgroup and adjusted analyses)
		20c	Definition of analysis population relating to protocol non-adherence (eg, as randomised analysis), and any statistical methods to handle missing data (eg, multiple imputation)
	**Methods: Monitoring**		
X	Data monitoring	21a	Composition of data monitoring committee (DMC); summary of its role and reporting structure; statement of whether it is independent from the sponsor and competing interests; and reference to where further details about its charter can be found, if not in the protocol. Alternatively, an explanation of why a DMC is not needed
		21b	Description of any interim analyses and stopping guidelines, including who will have access to these interim results and make the final decision to terminate the trial

For peer review only - http://bmjopen.bmj.com/site/about/guidelines.xhtml

Study protocol: a multicentre, randomised controlled trial to investigate the effects of parental touch on relieving acute procedural pain in neonates (Petal)

X	Harms	22	Plans for collecting, assessing, reporting, and managing solicited and spontaneously reported adverse events and other unintended effects of trial interventions or trial conduct	
	Auditing	23	Frequency and procedures for auditing trial conduct, if any, and whether the process will be independent from investigators and the sponsor	The project management group (RS, RP, AB, AH, DC, MC, FM) meets monthly throughout the trial to oversee trial conduct and recruitment.
	**Ethics and dissemination**			
X	Research ethics approval	24	Plans for seeking research ethics committee/institutional review board (REC/IRB) approval	
	Protocol amendments	25	Plans for communicating important protocol modifications (eg, changes to eligibility criteria, outcomes, analyses) to relevant parties (eg, investigators, REC/IRBs, trial participants, trial registries, journals, regulators)	The Investigator will submit and, where necessary, obtain approval from the relevant parties for all substantial amendments to the original approved documents.
X	Consent or assent	26a	Who will obtain informed consent or assent from potential trial participants or authorised surrogates, and how (see Item 32)	
		26b	Additional consent provisions for collection and use of participant data and biological specimens in ancillary studies, if applicable	
X	Confidentiality	27	How personal information about potential and enrolled participants will be collected, shared, and maintained in order to protect confidentiality before, during, and after the trial	

For peer review only - http://bmjopen.bmj.com/site/about/guidelines.xhtml

Study protocol: a multicentre, randomised controlled trial to investigate the effects of parental touch on relieving acute procedural pain in neonates (Petal)

X	Declaration of interests	28	Financial and other competing interests for principal investigators for the overall trial and each study site	
	Access to data	29	Statement of who will have access to the final trial dataset, and disclosure of contractual agreements that limit such access for investigators	Only the project team will have access to the final trial dataset. After the publication of results, anonymised data can be made available to other researchers upon reasonable request.
	Ancillary and post-trial care	30	Provisions, if any, for ancillary and post-trial care, and for compensation to those who suffer harm from trial participation	The University of Oxford has a specialist insurance policy in place which would operate in the event of any participant suffering harm as a result of their involvement in the research (Newline Underwriting Management Ltd, at Lloyd’s of London). NHS indemnity operates in respect of the clinical treatment which is provided.
X	Dissemination policy	31a	Plans for investigators and sponsor to communicate trial results to participants, healthcare professionals, the public, and other relevant groups (eg, via publication, reporting in results databases, or other data sharing arrangements), including any publication restrictions	

Study protocol: a multicentre, randomised controlled trial to investigate the effects of parental touch on relieving acute procedural pain in neonates (Petal)

		31b	Authorship eligibility guidelines and any intended use of professional writers	
		31c	Plans, if any, for granting public access to the full protocol, participant-level dataset, and statistical code	
	**Appendices**			
X	Informed consent materials	32	Model consent form and other related documentation given to participants and authorised surrogates	
	Biological specimens	33	Plans for collection, laboratory evaluation, and storage of biological specimens for genetic or molecular analysis in the current trial and for future use in ancillary studies, if applicable	No biological specimens are collected.

*It is strongly recommended that this checklist be read in conjunction with the SPIRIT 2013 Explanation & Elaboration for important clarification on the items. Amendments to the protocol should be tracked and dated. The SPIRIT checklist is copyrighted by the SPIRIT Group under the Creative Commons "Attribution-NonCommercial-NoDerivs 3.0 Unported" license.

For peer review only

BMJ Open

Study protocol: a multicentre, randomised controlled trial to investigate the effects of parental touch on relieving acute procedural pain in neonates (Petal)

Journal:	*BMJ Open*
Manuscript ID	bmjopen-2022-061841.R1
Article Type:	Protocol
Date Submitted by the Author:	15-Jun-2022
Complete List of Authors:	Cobo, Maria; University of Oxford, Department of Paediatrics; Universidad San Francisco de Quito, Colegio de Ciencias Biologicas y Ambientales<br>Moultrie, Fiona; University of Oxford, Department of Paediatrics<br>Hauck, Annalisa; University of Oxford, Department of Paediatrics<br>Crankshaw, Daniel; University of Oxford, Department of Paediatrics<br>Monk, Vaneesha; University of Oxford, Department of Paediatrics<br>Hartley, Caroline; University of Oxford, Department of Paediatrics<br>Evans Fry, Ria; University of Oxford, Department of Paediatrics<br>Robinson, Shellie; University of Oxford, Department of Paediatrics<br>van der Vaart, Marianne; University of Oxford, Department of Paediatrics<br>Baxter, Luke; University of Oxford, Department of Paediatrics<br>Adams, Eleri; Oxford University Hospitals NHS Foundation Trust, Newborn Care Unit, John Radcliffe Hospital<br>Poorun, Ravi; Royal Devon University Healthcare NHS Foundation Trust, Children's Services; University of Exeter, College of Medicine & Health<br>Bhatt , Aomesh ; University of Oxford, Department of Paediatrics<br>Slater, Rebeccah; University of Oxford, Department of Paediatrics
**Primary Subject Heading**:	Paediatrics
Secondary Subject Heading:	Neurology, Paediatrics, Radiology and imaging
Keywords:	NEONATOLOGY, NEUROPHYSIOLOGY, PAIN MANAGEMENT

SCHOLARONE™
Manuscripts

Study protocol: a multicentre, randomised controlled trial to investigate the effects of parental touch on relieving acute procedural pain in neonates (Petal)

Maria M Cobo[1,2], Fiona Moultrie[1], Annalisa Hauck[1], Daniel Crankshaw[1], Vaneesha Monk[1], Caroline Hartley[1], Ria Evans Fry[1], Shellie Robinson[1], Marianne van der Vaart[1], Luke Baxter[1], Eleri Adams[3], Ravi Poorun[4,5], Aomesh Bhatt[1], Rebeccah Slater[1]*

[1] Department of Paediatrics, University of Oxford, Oxford, UK
[2] Universidad San Francisco de Quito USFQ, Colegio de Ciencias Biologicas y Ambientales, Quito, Ecuador
[3] Newborn Care Unit, John Radcliffe Hospital, Oxford University Hospitals NHS Foundation Trust, Oxford, UK
[4] Children's Services, Royal Devon University Healthcare NHS Foundation Trust
[5] College of Medicine & Health, University of Exeter

Corresponding author: Rebeccah Slater; rebeccah.slater@paediatrics.ox.ac.uk

For peer review only

For peer review only - http://bmjopen.bmj.com/site/about/guidelines.xhtml

ABSTRACT

Introduction

Newborn infants routinely undergo minor painful procedures as part of postnatal care, with infants born sick or premature requiring a greater number of procedures. As pain in early life can have long-term neurodevelopmental consequences and lead to parental anxiety and future avoidance of interventions, effective pain management is essential. Non-pharmacological comfort measures such as breastfeeding, swaddling, and sweet solutions are inconsistently implemented and are not always practical or effective in reducing the transmission of noxious input to the brain. Stroking of the skin can activate C-tactile fibres and reduce pain, and therefore could provide a simple and safe parent-led intervention for the management of pain. The trial aim is to determine whether parental touch prior to a painful clinical procedure provides effective pain relief in neonates.

Methods and analysis

This is a multicentre blinded randomised controlled trial. A total of 112 neonates born at 35 weeks' gestation or more requiring a blood test in the first week of life will be recruited and randomised to receive parental stroking either pre- or post-procedure. We will monitor brain activity (EEG), cardiac and respiratory dynamics, oxygen saturation and facial expression to provide proxy pain outcome measures. The primary outcome will be the reduction of noxious-evoked brain activity in response to a heel lance. Secondary outcomes will be a reduction in clinical pain scores (Premature Infant Pain Profile-Revised), post-procedural tachycardia and parental anxiety.

Ethics and dissemination

The study has been approved by the London – South East Research Ethics Committee (ref:21/LO/0523). The results will be widely disseminated through peer-reviewed publications, international conferences and via our partner neonatal charities Bliss and SSNAP. If the parental tactile intervention is effective, recommendations will be submitted via the NHS clinical guideline adoption process.

Study Status: Commenced September 2021.

Trial Registration: ClinicalTrials.gov NCT04901611; **ISRCTN**: 14135962.

Strengths and limitations of this study
• Petal is a randomised controlled trial investigating whether noxious-evoked brain activity is reduced by pre-procedural parental stroking.
• The trial is based upon published evidence from two mechanistic studies which show a reduction in noxious-evoked brain activity during a heel lance or experimental stimuli in neonates whose skin was brushed by the experimenter prior to the procedure.
• This trial investigates stroking as a simple, free, low-risk, non-pharmacological pain-relieving intervention delivered by parents to their newborn infants in the first week of life.
• The trial employs multiple proxy measures to determine the impact of the stroking intervention on neonatal pain and investigates the impact of the intervention on parental anxiety and distress.
• While investigators cannot be blinded to the group allocation at the time of the study, this limitation is mitigated by ensuring that participants and investigators involved in all other aspects of the trial, including data analysis, are blinded.

For peer review only - http://bmjopen.bmj.com/site/about/guidelines.xhtml

INTRODUCTION

Background

Newborn infants undergo painful procedures as part of routine neonatal care. Sick or premature infants experience an average of ten painful procedures per day as part of life-sustaining treatment.[1] It is recognised that repetitive exposure to pain in early life can cause short-term physiological instability as well as long-term neurodevelopmental consequences such as reduced growth, altered structural and functional brain development, and reduced school-age academic performance.[2] Furthermore, repeatedly witnessing their infant in pain can have a significant negative impact on the emotional and psychological wellbeing of parents.[3–5] Effective pain management is therefore essential in neonatal care. However, measuring pain in this non-verbal patient population is challenging, and few safe and effective analgesics have been tested and approved for use in infants. Non-pharmacological strategies have been introduced and promoted over the last few decades for the management of acute procedural pain. Sweet-taste solutions such as sucrose are effective in relieving behavioural responses following minor painful procedures[6] but do not reduce noxious input to the brain.[7] This has caused concern that this intervention may not mitigate the long-term consequences of early life pain, and furthermore, it may have long-term neurodevelopmental effects with repeated use.[8–10] Breastfeeding also reduces behavioural and physiological responses to pain in full-term infants undergoing heel lancing, intramuscular injection, and venepuncture.[11] However, this strategy can be challenging for new mothers, is not always practical to implement in premature and critically ill infants or in mothers with transmissible infections, or due to maternal concerns of developing a negative association with breastfeeding.[12] Other comfort measures include swaddling and facilitated tucking of infants, which, although useful, are less effective in reducing pain.[13] While many studies have reported the potential pain-relieving effects of tactile interventions such a skin-to-skin care[14] and massage[15–21] in the context of minor painful procedures, these non-pharmacological interventions are scarcely used in maternity and neonatal units[22,23] and the mechanisms underpinning their effectiveness are still being established. Despite guidelines recommending the use of non-pharmacological interventions for pain relief, uptake of these practices remains poor and inconsistent.[24,25]

Measuring pain in infants

The assessment of pain and analgesia in infants primarily relies upon measuring changes in infant behaviour. One of the most common validated clinical pain tools is the Premature Infant Pain Profile (original PIPP, revised PIPP-R).[26,27] While subjective evaluations of behavioural responses are a gold standard for the clinical assessment of neonatal pain, electrophysiology based methods have more recently been developed to identify a pattern of noxious-evoked brain activity.[28–30] This objective and quantifiable neurophysiological measure has been previously used in pilot studies[31,32] and as the primary outcome measure in randomised clinical trials published in *The Lancet,* assessing the analgesic efficacy of sucrose[7] and morphine.[33] Noxious-evoked brain activity has specifically been well-characterised in response to heel lancing,[28–30,34] a clinical procedure which is frequently performed in neonates for blood collection, and will be used as the primary outcome of the Petal trial to investigate the efficacy of pre-procedural parental stroking.

Rationale

Maternal touch behaviours are instinctive, evolutionarily conserved amongst mammals.[35] Previous studies suggest there may also be a potential relationship between enhanced maternal touch and infant growth and development.[36,37] Stroking, by repeatedly applying gentle pressure to the skin, can activate C-tactile (CT) fibres, a subclass of slow-conducting unmyelinated sensory neurons, mostly found in hairy skin.[38–40] These fibres project to brain regions associated with affective processing such as the insular cortex, prefrontal cortex, superior temporal sulcus, and cingulate cortex[41–45] and are thought to have evolved to promote affiliative behaviours and social touch.[46–49] CT fibres are optimally activated by stroking at a velocity of 3cm/s (optimal range 1–10cm/s)[50–52], and studies in adults of gentle brushing or stroking paradigms at this optimal velocity have demonstrated a reduction in pain ratings[53,54] and noxious-evoked brain activity.[54] CT-optimal stimulation therefore could provide a natural and safe pain-relieving intervention.

For peer review only - http://bmjopen.bmj.com/site/about/guidelines.xhtml

We previously conducted a small prospective cohort study of pre-procedural stroking for pain relief in neonates, in which we demonstrated that CT-optimal stroking (at 3cm/s) prior to an experimental noxious stimulus or clinical heel lance significantly reduced noxious-evoked brain activity in term neonates compared to no touch intervention.[31] We replicated this study in an independent sample of term neonates and showed consistent results and a similar effect size in the group receiving the stroking intervention.[32] However, in both of these studies stroking was delivered by the researcher using a soft experimental brush with a known force. Although the studies did not identify a significant effect of the intervention on a clinical pain score, it was notably not powered to investigate this. Considering CT-optimal stroking is a natural maternal behaviour[55,56] and evidence suggesting that CT fibres respond optimally to touch at human skin temperature,[57] hands-on parental stroking has the potential to provide even greater benefit than CT-optimal brushstrokes. Pilot work further suggests that stroking a neonate has similar efficacy to researcher-led experimental brushing (unpublished).

Aim and Objectives

In the Petal trial, we aim to determine whether parental stroking prior to a common painful clinical procedure (heel lancing) provides effective analgesia in neonates. The primary outcome will be the reduction of noxious-evoked brain activity during a heel lance. Secondary outcomes will be a reduction in clinical pain scores, post-procedural tachycardia and parental anxiety (Table 1). Exploratory outcomes will investigate changes in brain activity during the intervention, as well as effects on physiological recovery post-procedure (using heart rate and respiratory dynamics) and further explore parental anxiety, distress, and attitudes to research.

For peer review only

For peer review only - http://bmjopen.bmj.com/site/about/guidelines.xhtml

Objectives	Outcome Measures
**Primary Objective**	**Primary Outcome Measure**
(i) To test whether parental touch prior to the clinical procedure reduces noxious-evoked brain activity following a heel lance.	(i) Magnitude of noxious-evoked brain activity following a heel lance (EEG data recorded in the 1000ms period following each heel lance).
**Secondary Objectives**	**Secondary Outcome Measure**
(i) To test whether parental touch prior to the clinical procedure reduces clinical pain scores (PIPP-R) during the 30-second period after the heel lance.	(i) PIPP-R score during the 30-second period after the heel lance.
(ii) To test whether parental touch prior to the clinical procedure reduces incidence of post-procedural tachycardia activity following a heel lance.	(ii) Percentage of neonates who develop tachycardia in the 30-second post-heel lance.
(iii) To test whether parental touch prior to the clinical procedure reduces parental anxiety, compared with post-procedural touch.	(iii) Difference in STAI-S scores pre- and post-procedure.
**Exploratory Objectives**	**Exploratory Outcome Measures**
(i) To explore how parental touch impacts background brain activity.	(i) Changes in brain activity during touch intervention.
(ii) To explore whether parental touch prior to the clinical procedure reduces the duration of time for heart rate to return to baseline after a heel lance.	(ii) Time taken for heart rate to return to baseline post-heel lance.
(iii) To explore how parental touch prior to the clinical procedure affects respiratory stability.	(iii) Post-procedural respiratory dynamics and incidence of apnoea.
(iv) To explore parental anxiety and distress, and their experience of the trial and infant research.	(iv) Scores for individual parameters from the STAI-T and STAI-S; 4-point distress questionnaire score; responses to survey about participation in Petal and infant research.

Table 1. Objectives and Outcome Measures. STAI-T: State-Trait Anxiety Inventory-Trait, STAI-S: State-Trait Anxiety Inventory-State, PIPP-R: Premature Infant Pain Profile – Revised.

For peer review only - http://bmjopen.bmj.com/site/about/guidelines.xhtml

For peer review only

METHODS AND ANALYSIS

Trial Description

This is a multicentre randomised-controlled interventional trial, with two research sites (John Radcliffe Hospital, Oxford, and Royal Devon and Exeter Hospital, Devon, UK). The parents of eligible neonates satisfying inclusion criteria (Figure 1) will be approached by a member of the research team. Parental written informed consent will be taken and neonates will be electronically randomised to receive parental stroking either prior to or after a clinically-required heel lance. Patient information leaflets and consent forms are available as supplementary file 1. A unique study ID will be assigned to each individual participant. The randomisation program will use a minimisation algorithm to ensure approximate balance between the groups with respect to gestational age at birth, postnatal age at time of randomisation, sex, the indication for blood sampling, and research site. The users of the system will be blind to the next allocation.

Each neonate will be studied on a single test occasion lasting approximately one hour (Figure 2) and will not require further follow-up. A parent will first complete the State-Trait Anxiety Inventory-Trait (STAI-T) and State-Trait Anxiety Inventory-State (STAI-S) questionnaires, which will be administered verbally by a researcher. This will allow assessment of both trait anxiety and state anxiety prior to the commencement of the stroking intervention or blood test. At least 30 minutes prior to the heel lance, the research team will set up physiological monitoring including electrocardiography (ECG) and pulse oximetry for continuous recording of baseline cardiorespiratory stability. Electroencephalography (EEG) electrodes will then be sited to allow continuous monitoring of baseline brain activity for at least 10 minutes prior to the clinical procedure. A control heel lance will then be performed followed by the clinical heel lance.

The control heel lance is a non-noxious sham procedure whereby the lancet is placed against the participant's foot rotated at 90 degrees, preventing release of the blade into the foot. This procedure simulates the tactile and auditory aspects of the blood sampling experience without the noxious input. Brain activity, physiology and facial expression (video) will be recorded for both the control heel lance and clinical heel lance to allow assessment of outcome measures including noxious-evoked brain activity, PIPP-R scores, tachycardia, and respiratory dynamics (Table 1). The heel lance and control stimulus will be linked electronically to the recording equipment as described in previous studies,[7,28,29] providing precise timing of when the heel lance occurs. In the event of the neonate requiring multiple heel lances, data will only be included from the first heel lance (conditional on data quality). Video monitoring of the face will commence approximately 30 seconds prior to the control lance and end at least 30 seconds after the clinical heel lance to allow PIPP-R scoring. For neonates randomised to receive pre-procedural stroking, the parent will be instructed to begin stroking down the infant's leg immediately prior to the clinical heel lance, with the aid of an animated visual cue to help maintain a velocity of 3cm/s and a duration of 10 seconds. After the heel lance, blood collection will be delayed for 30 seconds to allow PIPP-R scoring. For neonates randomised to receive post-procedural stroking, the parent will be instructed to begin stroking down the infant's leg after the start of blood collection, when deemed appropriate by the clinician performing the heel lance in order to ensure that blood collection is not disrupted. Parents will be guided by an animated visual cue. A researcher will then verbally administer the STAI-S and 4-point distress questionnaire after the procedure is completed. Physiological monitoring will continue for 30 minutes and EEG monitoring for at least 10 minutes to allow investigation of post-procedural cardiorespiratory dynamics and brain activity as exploratory outcomes of the trial. Finally, the parents will be invited to complete an anonymous survey of their experience and views on research after completion of the study. This study protocol follows the Standard Protocol Items: Recommendations for Interventional Trials (SPIRIT) guidelines (supplementary file 2).[58]

Intervention

The parental touch intervention will involve one parent stroking the infant's leg for 10 seconds. The duration of the intervention is consistent with previous studies.[31,32,53,59] A member of the research team will inform parents of their randomised allocation (either stroking pre-heel lance or post-heel lance) at the start of the test occasion. They will explain and demonstrate how to administer the intervention using their whole hand, stroking in one direction down towards the foot. The infant will lay in a cot during the intervention and procedure. During the demonstration

For peer review only - http://bmjopen.bmj.com/site/about/guidelines.xhtml

and test occasion, PsychoPy software[60] will be used to provide a visual cue on a computer screen to guide a consistent stroking speed of 3cm/s for 10 seconds. During the study all neonates received comfort care in accordance with the local practice guidelines. These measures included swaddling the infants and providing non-nutritive sucking.

Recording Techniques

Electroencephalography (EEG)

Electrophysiological activity will be acquired with the SynAmps RT 64-channel headbox and amplifiers (Compumedics Neuroscan) or with the Compumedics Grael V2 EEG system, with a bandwidth from DC - 400 Hz and a sampling rate of 2000 or 2048Hz. Data recorded at 2048Hz will be downsampled to 2000Hz prior to further processing. CURRYscan7 or CURRYscan8 neuroimaging suite (Compumedics Neuroscan) will be used to record the activity. All equipment will conform to the electrical safety standard for medical devices, IEC 60601-1. Eight EEG recording electrodes will be positioned on the scalp at Cz, CPz, C3, C4, FCz, T3, T4 and Oz according to the modified international 10-20 System. Reference and ground electrodes will be placed at Fz and the forehead respectively. EEG conductive paste will be used to optimise contact with the scalp. All impedances will be reduced to approximately 5kΩ by rubbing the skin with EEG preparation gel prior to electrode placement. An ECG electrode will be placed on the left clavicle to record heart rate.

Physiological Monitoring (ECG and Pulse Oximetry)

Heart rate, respiratory rate and oxygen saturation will be recorded continuously throughout the study period (approximately one hour) using ECG and pulse oximetry. Heart rate and oxygen saturation data will be used to calculate the clinical pain scores following the heel lance and control stimulus and to assess clinical stability across the test occasion.

Video Recording

Video recording will be used to measure behavioural responses i.e. changes in facial expression during the control stimulus and clinically-required heel lance. A synchronised LED flash will be activated by the researcher simultaneously with each stimulation as a marker for the time of stimulation.

Parental Questionnaire

The parent administering the intervention will be asked to complete a short series of validated electronic questionnaires assessing anxiety and distress at the start and end of the test occasion (Table 2). The researcher will record the responses to the STAI-T, STAI-S and distress questionnaire in an electronic Case Report Form. The electronic device will then be presented to the parent to independently complete a short survey about trial participation and their research experience. The survey will be completed anonymously, and responses will be stored by trial arm with no link to study IDs.

Questionnaire section	Topic	Timing of administration	Questionnaire administrator
20-point State-Trait Anxiety Inventory (STAI)-T	Trait anxiety	Start of test occasion	Administered verbally by researcher
20-point State-Trait Anxiety Inventory (STAI)-S	State anxiety pre-heel lance<br><br>State anxiety post-heel lance	Start of test occasion<br><br>After the procedure and intervention are completed	Administered verbally by researcher<br><br>Administered verbally by researcher
4-point distress questionnaire	Emotional constructs experienced at time of the clinical heel lance	After the procedure and intervention are completed	Administered verbally by researcher

For peer review only - http://bmjopen.bmj.com/site/about/guidelines.xhtml

Anonymous survey	Views on the trial and infant research	End of test occasion	Completed by parent

Table 2. Trial parental questionnaires.

State-Trait Anxiety Inventory (STAI)

The State-Trait Anxiety Inventory (STAI) is the gold standard assessment for state anxiety.[61] It is well validated, publicly available and has a trait (STAI-T) version consisting of 20 statements exploring general feelings of anxiety, and a state version (STAI-S) consisting of 20 statements exploring anxiety levels at a particular point in time. Each question is rated on a 4-point scale. The range of possible scores for the STAI varies from a minimum score of 20 to a maximum score of 80 on both the STAI-T and STAI-S subscales. Scores are classified as "no or low anxiety" (20-37), "moderate anxiety" (38-44), and "high anxiety" (45-80).

4-point distress questionnaire

Parents will be asked four questions related to their emotions during the clinical heel lance procedure.[62,63] Each of the four emotional constructs (worried, upset, anxious and sad) will be rated on an 11-point scale ranging from "not at all" (0) to "extremely" (10). A total score between 0 and 40 will be calculated, where higher scores are indicative of greater parental distress. This score is frequently used in research to evaluate parent/child interactions during painful procedures.[62–64]

Outcome Measures

Noxious-evoked Brain Activity

An EEG template that reflects the noxious-evoked brain activity in neonates has previously been defined using principal component analysis, validated in independent data sets[30] and used in clinical studies and a clinical trial.[33] This template will be projected onto the EEG data recorded in the 1000ms period following each heel lance and control heel lance stimulus and the relative weight of the component calculated for each neonate. A greater weight indicates a stronger noxious-evoked response. While the brain activity characterised is directly related to noxious input, it does not reflect all noxious-evoked activity across the brain or all aspects of the pain experience. The response to the non-noxious control heel lance stimulus is being recorded to confirm that it significantly differs from the brain activity evoked by a noxious heel lance. This forms an important data quality control check.[28]

PIPP-R Score

Clinical pain scores will be evaluated using the validated Premature Infant Pain Profile - Revised,[27] which is a composite multimodal measure encompassing behavioural, physiological and contextual indicators of the pain response. It allows for different aspects of the infant pain experience to be captured and has been widely used as the primary outcome measure for infant pain in many clinical trials.[65–67] The PIPP-R score will be calculated for the control heel lance and the clinical heel lance procedure. Heart rate, oxygen saturation and facial expression will be recorded in the 15-second period before and 30-second period after each of the procedures.[26,27] The 15-second period before the heel lance will be recorded immediately prior to the stroking intervention. Videos of the infant's facial expressions will be scored offline using the PIPP-R facial coding system. Changes in heart rate and oxygen saturation will be recorded with ECG and pulse oximeter and used to calculate the PIPP-R score. For each participant, PIPP-R scores will be assessed by investigators blinded to the study arm. A second investigator (blinded to the trial arm) will re-calculate 20% of the PIPP-R scores to measure inter-rater reliability.

Clinical Stability

Clinical stability will be assessed in the 30-minute periods before and after the heel lance. The percentage of neonates who develop post-procedural tachycardia in the 30 seconds post-heel lance will be a secondary outcome measure of the trial. Tachycardia will be defined as a heart rate >160 beats per minute as per Advanced Paediatric Life Support guidelines, reflecting heart rate values >90th centile for newborn infants in the first week of life.[68,69] Exploratory outcome measures will also include the time taken for the heart rate to return to baseline values post-heel lance and respiratory rate variability in the 30 minutes prior and post heel lance (including incidence of apnoea). An episode of apnoea will be defined as the cessation of breathing for at least 20 seconds.[70]

Parental Experience

For peer review only - http://bmjopen.bmj.com/site/about/guidelines.xhtml

Parental anxiety will be quantified using the outcomes of the STAI-T and STAI-S questionnaires. Parental distress will be quantified using the 4-point distress score. The anonymous parent survey will assess the parental experience of the trial and parental views on taking part in the trial.

Statistics and Analysis

Analysis of outcome measures

Data pre-processing and statistical analysis will be performed blind to treatment allocation. The analysis and presentation of results will follow the most up-to-date recommendations of the CONSORT group. All comparative analyses will be performed using MatlabR2020a or an updated version, adjusting for important prognostic factors where possible: these are likely to be a subset of the minimisation factors at randomisation. A full statistical analysis plan will be finalised before any comparative analysis of outcome measures is performed.

Significance Levels

For the analysis of the primary outcome measure, a p-value of 0.05 (two-sided 5% significance level) will be used to indicate statistical significance. Significance levels for secondary outcomes will be corrected for multiple comparisons and the method will be specified in the analysis plan. Two-sided statistical tests and corresponding p-values will be presented throughout; however, for the purposes of interpretation of results, confidence intervals will dominate, rather than p-values.

Primary

Noxious-evoked brain activity

The projected weight of the template will be compared between the two groups using linear regression, dependent on the mean and variance of the data. If appropriate, we will present the mean and standard deviation for each group and estimate the adjusted mean difference with a 95% confidence interval. If the outcome data are skewed, we will present the median and interquartile range (or entire range, whichever is appropriate) for each group, and estimate the median difference between groups with a corresponding 95% confidence interval.

Secondary

PIPP-R score

PIPP-R scores (during the 30-second period after heel lance) in the two groups will be compared using linear regression to estimate the adjusted mean difference. If the PIPP-R scores are skewed, we will present the median and interquartile range (or entire range, whichever is appropriate) for each group, and estimate the median difference between groups.

Clinical stability (tachycardia)

The percentage of infants experiencing tachycardia will be compared between the two groups. Depending on the distribution of these counts, either Poisson or linear regression will be used to calculate the adjusted effect estimates. If appropriate, adjustments for the 30-minute baseline period will also be made. Alternatively, we will present the medians and interquartile ranges (or entire range, whichever is appropriate) for each group and estimate the median differences between groups. In the event of occurrences being very infrequent, logistic regression may be used as an alternative.

Parental anxiety

STAI scores in the two groups will be compared to estimate the adjusted mean difference in pre-procedural and post-procedural STAI-S scores. If the scores are skewed, we will present the median and interquartile range (or entire range, whichever is appropriate) for each group, and estimate the median difference between groups.

Exploratory

Exploratory analyses will be conducted to investigate i) the effects of parental touch on background brain activity, ii) whether pre-procedural parental touch reduces the duration of time for heart rate to return to baseline, iii) the effect of pre-procedural parental touch on respiratory rate variability, respiratory dynamics and the incidence of apnoea and iv) the parental experience of the procedure and involvement in research.

For peer review only - http://bmjopen.bmj.com/site/about/guidelines.xhtml

Sample Size Determination

Power Calculation

The assumptions for these calculations are based on data from mechanistic studies investigating the effect of (experimenter-led) soft brushing of the skin at CT-optimal rate on the response to an experimental noxious stimulus or clinical heel lance in term neonates.[31,32] The mean (SD) brain activity evoked by heel lancing in the control group is estimated to be 1.07 (0.66). A 40% reduction in the intervention group is considered to be clinically significant and realistic from other studies.[31,54] With 90% power and a two-sided 5% significance level, to observe a 40% reduction in brain activity, a sample size of 102 would be required. Allowing for 10% loss, due to technical difficulties or other clinical issues, this increases to 112.

Missing Data

Missing data may occur in our trial due to equipment failure, EEG artefacts, or clinical issues resulting in withdrawal post-randomisation. If missing data exists, we expect it will occur at random, and collected data will be representative of the population. To account for potential missing data, we have inflated our sample size by 10%. The analysis will be conducted using the available data.

Ethics and dissemination

The trial has been approved by the London South East Research Ethics Committee (ref:21/LO/0523) and will be conducted in accordance with Good Clinical Practice and the Declaration of Helsinki. EEG is a safe tool used routinely in clinical practice and research to measure brain activity. Surface electrodes are used and temporarily fixed without glue. All heel lances performed during the trial will have been requested by the clinical team responsible for the infant's medical care. No extra blood tests or noxious procedures will be performed for the purpose of the study. Every effort will be made to minimise inconvenience and prevent disruption of clinical care. There are no expected serious adverse events (SAE) for this trial. Any SAEs identified will be reported to the CI within 24 hours and they will report any unexpected SAEs deemed related to the trial to the REC and Sponsor in accordance with REC/HRA guidance.

Parent(s) may withdraw their neonate from the trial at any time and they are not obliged to give a reason. If parents choose to withdraw their child after the study has begun, they will be asked whether data already collected may be retained and used for the purposes of the trial. Parents will be made aware that this decision has no impact on any aspects of their infant's continuing care. The attending clinician may also withdraw the neonate from the trial if they consider this to be in their best interest. If any of the exclusion criteria manifest prior to data collection, the participant will be withdrawn.

The results of the study will be disseminated to the scientific and wider community through peer-reviewed publications and national and international meetings and conferences, via the charities SSNAP and Bliss, and through the NHS clinical guideline adoption process.

Patient and Public Involvement (PPI)

A PPI representative will be included in the extended PMG group and invited to join specific PMG meetings to discuss trial progress and developments. Bliss: for babies born premature or sick is a national UK neonatal charity, which is partly funding the trial. They will receive regular trial progress reports and promote the trial across their various channels, and disseminate the results. The research team will also work closely with the on-site local Oxford charity Supporting the Sick Newborn And their Parents (SSNAP) during the design, conduct and dissemination of the trial. SSNAP have reviewed all parent-facing materials, will review manuscripts reporting results, and will be involved in disseminating results to the public.

For peer review only - http://bmjopen.bmj.com/site/about/guidelines.xhtml

DISCUSSION

All newborn infants are exposed to clinically-necessary painful procedures. Even healthy neonates on postnatal wards can require repeated painful procedures beyond routine Newborn Screening, such as blood tests for glucose monitoring or jaundice, which can be distressing for both neonates and their parents. In the UK, more than 100,000 newborn infants receive neonatal care every year as a result of prematurity or illness,[71] which, for some, can entail weeks to months of hospitalisation and procedures. As such, improving the management of pain is recognised as a top neonatal UK research priority[72] and a major concern amongst parents and neonatal nurses.[73]

Poor management of neonatal pain can have a significant negative impact on parents. Mothers of hospitalised infants report feeling emotionally and psychologically traumatised due to having to allow their infants to undergo clinically-necessary painful procedures, and due to feelings of helplessness from being unable to protect or comfort their child.[3–5] Actively involving parents in care relieves parental distress[74] and increases the likelihood that infants receive treatment for pain.[1,5,75] Infant massage, a tactile comfort measure which involves patterns of stroking, has been shown to improve mother-infant bonding and improve postnatal depression,[76] a condition afflicting at least one in ten UK mothers in the first-year post-partum[77]. Furthermore, maternal stroking of infants in general has been shown to moderate the behavioural and physiological effects of maternal depression on infants.[78] Promoting the natural tactile behaviour of stroking to provide evidence-based pain-relief would therefore be beneficial to both mothers and infants.

Anxiety about pain is increasingly recognised as a key factor in parental refusal for procedures such as vitamin-K intramuscular injections at birth[79] and immunisations.[80–82] Avoidance of key interventions in early life could have drastic consequences for child health and this issue must be addressed. Indeed, parental anxiety and attitudes during painful procedures can also impact neonatal distress and subsequent pain experience during clinical procedures in later life.[83] Parental anxiety regarding pain could be alleviated by empowering parents to provide safe and effective pain relief for their child. Unlike other non-pharmacological interventions, this strategy could be broadly implemented regardless of feeding status of the infant or availability of a product like sucrose, in hospital as well as the community, and across high and low resource clinical settings.

CT-fibres likely provide the neurobiological mechanism underlying the benefits of tactile stimulation in early life. Studies have revealed that mothers instinctively stroke their infants at a CT-optimal rate[55,56] and that this tactile stimulation is beneficial. CT-optimal touch significantly decreases resting heart rates in infants aged 1-4 months[59] and 9-months,[84] as well as in premature infants (28–36 weeks' gestation).[85] Recent studies have also investigated the neurological correlates of CT-optimal touch in early life. In 2-month-old infants, CT-optimal touch produces greater activation of the insular cortex compared to CT non-optimal touch.[85] Similarly, in term infants CT-optimal stroking with a soft brush produces activation of the primary somatosensory and posterior insular cortices,[86] suggesting that the neonatal brain is sensitive to the somatosensory and socio-affective effects of CT-optimal stroking.

The Petal trial is based upon clear mechanistic evidence from preliminary cohort studies and is, as such, adequately powered to address the clinical question. It employs a range of multimodal outcomes, including electrophysiological, behavioural, and cardiorespiratory measures, to cover the many aspects of pain experience, and seeks to investigate the benefits of the intervention to both neonates and their parents. Blinding of outcome assessment is being performed to ensure the integrity of the trial as it is not possible to blind the researchers at the time of study due to the nature of the intervention. Although parents instinctively stroke at the optimal velocity to stimulate CT-fibres,[55,56] consistency of the intervention is standardised across the trial by providing an animated visual aid for parents to follow. In the event of a positive trial outcome, the intervention could next be translated to more premature infants and other minor painful skin-breaking procedures performed frequently in infants such as immunisation and cannulation and could be performed by parents or healthcare workers in the absence of parents. The Petal trial investigates a simple, free, low-risk, non-pharmacological pain-relieving intervention, which could be rapidly incorporated into routine clinical practice, benefiting infants, their parents, and the wider community.

For peer review only - http://bmjopen.bmj.com/site/about/guidelines.xhtml

Trial status

Participant recruitment is currently ongoing. Protocol version no. 3.0 (date of submission: 3 February 2022).

Acknowledgements

The Authors thank the Wellcome trust and Bliss Charity for helping to fund the study, as well as the neonatal team, parents and babies who will help throughout the study.

FOOTNOTES

Author contributions

MC: Conceptualization, Methodology, Software, Investigation, Data Curation, Writing - Original Draft, Writing - Review & Editing, Visualization, Project Administration. **FM:** Conceptualization, Methodology, Investigation, Writing - Original Draft, Writing - Review & Editing, Funding Acquisition. **AH:** Methodology, Investigation, Data Curation, Writing - Review & Editing, Project Administration. **DC:** Methodology, Software, Investigation, Data Curation, Writing - Review & Editing. **VM:** Conceptualization, Methodology, Writing - Original Draft, Project Administration, Funding Acquisition. **CH:** Software, Methodology, Data Curation, Funding Acquisition. **REF:** Conceptualization, Methodology, Investigation, Writing - Original Draft. **SR:** Investigation. **MvdV:** Software, Validation, Data Curation, Writing - Review & Editing. **LB:** Methodology, Data Curation, Writing - Review & Editing. **EA:** Conceptualization, Methodology, Writing - Review & Editing, Supervision. **RP:** Conceptualization, Methodology, Investigation, Data Curation, Supervision, Project Administration. **AB:** Conceptualization, Methodology, Supervision, Writing - Original Draft, Project administration, Funding Acquisition. **RS:** Conceptualization, Methodology, Resources, Writing - Original Draft, Writing - Review & Editing, Supervision, Project Administration, Funding Acquisition.

Funding

This work is supported by The Wellcome Trust via a senior fellowship awarded to Rebeccah Slater (grant number 207457/Z/17/Z) and by Bliss (a UK charity) via a research grant (grant number N/A).

Competing interests

None declared.

Ethics approval

London South East Research Ethics Committee (ref:21/LO/0523).

Figure legend

Figure 1. Trial flowchart. IVH: Intraventricular haemorrhage.

Figure 2. Trial procedures

REFERENCES

1 Carbajal R, Rousset A, Danan C, *et al.* Epidemiology and treatment of painful procedures in neonates in intensive care units. *JAMA* 2008;300:60–70.

2 Walker SM. Long-term effects of neonatal pain. *Semin Fetal Neonatal Med* 2019;24:101005.

3 Franck LS, Allen A, Cox S, *et al.* Parents' views about infant pain in neonatal intensive care. *Clin J Pain* 2005;21:133–9.

4 Gale G, Franck LS, Kools S, *et al.* Parents' perceptions of their infant's pain experience in the NICU. *Int J Nurs Stud* 2004;41:51–8.

5 Kyololo OM, Stevens BJ, Songok J. Mothers' Perceptions about Pain in Hospitalized Newborn Infants in

For peer review only - http://bmjopen.bmj.com/site/about/guidelines.xhtml

Kenya. *J Pediatr Nurs* 2019;47:51–7.

6 Stevens B, Yamada J, Lee GY, *et al.* Sucrose for analgesia in newborn infants undergoing painful procedures. *Cochrane database Syst Rev* 2013;:CD001069.

7 Slater R, Cornelissen L, Fabrizi L, *et al.* Oral sucrose as an analgesic drug for procedural pain in newborn infants: a randomised controlled trial. *Lancet* 2010;376:1225–32.

8 Tremblay S, Ranger M, Chau CMY, *et al.* Repeated exposure to sucrose for procedural pain in mouse pups leads to long-term widespread brain alterations. *Pain* 2017;158:1586–98.

9 Schneider J, Duerden EG, Guo T, *et al.* Procedural pain and oral glucose in preterm neonates: brain development and sex-specific effects. *Pain* 2018;159:515–25.

10 Ranger M, Tremblay S, Chau CMY, *et al.* Adverse behavioral changes in adult mice following neonatal repeated exposure to pain and sucrose. *Front Psychol* 2019;9:2394.

11 Benoit B, Martin-Misener R, Latimer M, *et al.* Breast-Feeding Analgesia in Infants: An Update on the Current State of Evidence. *J Perinat Neonatal Nurs* 2017;31:145–59.

12 Tansky C, Lindberg CE. Breastfeeding as a Pain Intervention When Immunizing Infants. *J Nurse Pract* 2010;6:287–95.

13 Meek J, Huertas A. Cochrane review: Non-nutritive sucking, kangaroo care and swaddling/facilitated tucking are observed to reduce procedural pain in infants and young children. *Evid Based Nurs* 2012;15:84–5.

14 Johnston C, Campbell-Yeo M, Disher T, *et al.* Skin-to-skin care for procedural pain in neonates. *Cochrane database Syst Rev* 2017;2:CD008435.

15 Jain S, Kumar P, McMillan DD. Prior leg massage decreases pain responses to heel stick in preterm babies. *J Paediatr Child Health* 2006;42:505–8.

16 Bellieni C V, Cordelli DM, Marchi S, *et al.* Sensorial saturation for neonatal analgesia. *Clin J Pain* 2007;23:219–21.

17 Diego MA, Field T, Hernandez-Reif M. Procedural pain heart rate responses in massaged preterm infants. *Infant Behav Dev* 2009;32:226–9.

18 Abdallah B, Badr LK, Hawwari M. The efficacy of massage on short and long term outcomes in preterm infants. *Infant Behav Dev* 2013;36:662–9.

19 Hathaway EE, Luberto CM, Bogenschutz LH, *et al.* Integrative Care Therapies and Physiological and Pain-related Outcomes in Hospitalized Infants. *Glob Adv Heal Med* 2015;4:32–7.

20 Chik Y-M, Ip W-Y, Choi K-C. The Effect of Upper Limb Massage on Infants' Venipuncture Pain. *Pain Manag Nurs Off J Am Soc Pain Manag Nurses* 2017;18:50–7.

21 Zargham-Boroujeni A, Elsagh A, Mohammadizadeh M. The Effects of Massage and Breastfeeding on Response to Venipuncture Pain among Hospitalized Neonates. *Iran J Nurs Midwifery Res* 2017;22:308–12.

22 Field T, Hernandez-Reif M, Feijo L, *et al.* Prenatal, perinatal and neonatal stimulation: a survey of neonatal nurseries. *Infant Behav Dev* 2006;29:24–31.

23 De Clifford-Faugère G, Aita M, Le May S. Nurses' practices regarding procedural pain management of preterm infants. *Appl Nurs Res* 2019;45:52–4.

24 Losacco V, Cuttini M, Greisen G, *et al.* Heel blood sampling in European neonatal intensive care units: compliance with pain management guidelines. *Arch Dis Child Fetal Neonatal Ed* 2011;96:F65-8.

25 Courtois E, Droutman S, Magny J-F, *et al.* Epidemiology and neonatal pain management of heelsticks in intensive care units: EPIPPAIN 2, a prospective observational study. *Int J Nurs Stud* 2016;59:79–88.

26 Stevens B, Johnston C, Petryshen P, *et al.* Premature Infant Pain Profile: development and initial validation. *Clin J Pain* 1996;12:13–22.

27 Stevens BJ, Gibbins S, Yamada J, *et al.* The premature infant pain profile-revised (PIPP-R): initial validation and feasibility. *Clin J Pain* 2014;30:238–43.

28 Slater R, Worley A, Fabrizi L, *et al.* Evoked potentials generated by noxious stimulation in the human infant brain. *Eur J Pain* 2010;14:321–6.

29 Worley A, Fabrizi L, Boyd S, *et al.* Multi-modal pain measurements in infants. *J Neurosci Methods* 2012;205:252–7.

30 Hartley C, Duff EP, Green G, *et al.* Nociceptive brain activity as a measure of analgesic efficacy in infants. *Sci Transl Med* 2017;9.

31 Gursul D, Goksan S, Hartley C, *et al.* Stroking modulates noxious-evoked brain activity in human infants.

For peer review only

For peer review only - http://bmjopen.bmj.com/site/about/guidelines.xhtml

Curr. Biol. 2018;28:R1380–1.

32 Cobo MM, Hartley C, Gursul D, *et al.* Quantifying individual noxious-evoked baseline sensitivity to optimise analgesic trials in neonates. *Elife* 2021;10.

33 Hartley C, Moultrie F, Hoskin A, *et al.* Analgesic efficacy and safety of morphine in the Procedural Pain in Premature Infants (Poppi) study: randomised placebo-controlled trial. *Lancet* 2018;392:2595–605.

34 Slater R, Fabrizi L, Worley A, *et al.* Premature infants display increased noxious-evoked neuronal activity in the brain compared to healthy age-matched term-born infants. *Neuroimage* 2010;52:583–9.

35 Hertenstein MJ, Verkamp JM, Kerestes AM, *et al.* The communicative functions of touch in humans, nonhuman primates, and rats: a review and synthesis of the empirical research. *Genet Soc Gen Psychol Monogr* 2006;132:5–94.

36 Fitri SYR, Nasution SK, Nurhidayah I, *et al.* Massage therapy as a non-pharmacological analgesia for procedural pain in neonates: A scoping review. *Complement Ther Med* 2021;59:102735.

37 Mrljak R, Arnsteg Danielsson A, Hedov G, *et al.* Effects of Infant Massage: A Systematic Review. Int. J. Environ. Res. Public Heal. . 2022;19.

38 Vallbo A, Olausson H, Wessberg J, *et al.* A system of unmyelinated afferents for innocuous mechanoreception in the human skin. *Brain Res* 1993;628:301–4.

39 Vallbo AB, Olausson H, Wessberg J, *et al.* Receptive field characteristics of tactile units with myelinated afferents in hairy skin of human subjects. *J Physiol* 1995;483 (Pt 3:783–95.

40 Liu Q, Vrontou S, Rice FL, *et al.* Molecular genetic visualization of a rare subset of unmyelinated sensory neurons that may detect gentle touch. *Nat Neurosci* 2007;10:946–8.

41 Olausson H, Lamarre Y, Backlund H, *et al.* Unmyelinated tactile afferents signal touch and project to insular cortex. *Nat Neurosci* 2002;5:900–4.

42 Morrison I, Björnsdotter M, Olausson H. Vicarious responses to social touch in posterior insular cortex are tuned to pleasant caressing speeds. *J Neurosci* 2011;31:9554–62.

43 McGlone F, Olausson H, Boyle JA, *et al.* Touching and feeling: differences in pleasant touch processing between glabrous and hairy skin in humans. *Eur J Neurosci* 2012;35:1782–8.

44 Bennett RH, Bolling DZ, Anderson LC, *et al.* fNIRS detects temporal lobe response to affective touch. *Soc Cogn Affect Neurosci* 2014;9:470–6.

45 Gordon I, Voos AC, Bennett RH, *et al.* Brain mechanisms for processing affective touch. *Hum Brain Mapp* 2013;34:914–22.

46 McGlone F, Wessberg J, Olausson H. Discriminative and affective touch: sensing and feeling. *Neuron* 2014;82:737–55.

47 von Mohr M, Kirsch LP, Fotopoulou A. The soothing function of touch: affective touch reduces feelings of social exclusion. *Sci Rep* 2017;7:13516.

48 Olausson H, Wessberg J, Morrison I, *et al.* The neurophysiology of unmyelinated tactile afferents. *Neurosci Biobehav Rev* 2010;34:185–91.

49 Morrison I, Löken LS, Minde J, *et al.* Reduced C-afferent fibre density affects perceived pleasantness and empathy for touch. *Brain* 2011;134:1116–26.

50 Löken LS, Wessberg J, Morrison I, *et al.* Coding of pleasant touch by unmyelinated afferents in humans. *Nat Neurosci* 2009;12:547–8.

51 Essick GK, McGlone F, Dancer C, *et al.* Quantitative assessment of pleasant touch. *Neurosci Biobehav Rev* 2010;34:192–203.

52 Triscoli C, Ackerley R, Sailer U. Touch satiety: differential effects of stroking velocity on liking and wanting touch over repetitions. *PLoS One* 2014;9:e113425.

53 Liljencrantz J, Strigo I, Ellingsen DM, *et al.* Slow brushing reduces heat pain in humans. *Eur J Pain* 2017;21:1173–85.

54 von Mohr M, Krahé C, Beck B, *et al.* The social buffering of pain by affective touch: a laser-evoked potential study in romantic couples. *Soc Cogn Affect Neurosci* 2018;13:1121–30.

55 Croy I, Luong A, Triscoli C, *et al.* Interpersonal stroking touch is targeted to C tactile afferent activation. *Behav Brain Res* 2016;297:37–40.

56 Van Puyvelde M, Gorissen A-S, Pattyn N, *et al.* Does touch matter? The impact of stroking versus non-stroking maternal touch on cardio-respiratory processes in mothers and infants. *Physiol Behav* 2019;207:55–63.

For peer review only

For peer review only - http://bmjopen.bmj.com/site/about/guidelines.xhtml

57 Ackerley R, Backlund Wasling H, Liljencrantz J, *et al.* Human C-tactile afferents are tuned to the temperature of a skin-stroking caress. *J Neurosci* 2014;34:2879–83.

58 Chan A-W, Tetzlaff JM, Altman DG, *et al.* SPIRIT 2013 Statement: Defining Standard Protocol Items for Clinical Trials. *Ann Intern Med* 2013;158:200–7.

59 Fairhurst MT, Löken L, Grossmann T. Physiological and behavioral responses reveal 9-month-old infants' sensitivity to pleasant touch. *Psychol Sci* 2014;25:1124–31.

60 Peirce J, Gray JR, Simpson S, *et al.* PsychoPy2: Experiments in behavior made easy. *Behav Res Methods* 2019;51:195–203.

61 Spiegelberger CD, Gorsuch RL, Lushene R, *et al. Manual for the State-Trait Anxiety Inventory*. Palo Alto, CA: : Consulting Psychologists Press 1983.

62 Caes L, Vervoort T, Eccleston C, *et al.* Parental catastrophizing about child's pain and its relationship with activity restriction: the mediating role of parental distress. *Pain* 2011;152:212–22.

63 Vervoort T, Karos K, Johnson D, *et al.* Parental emotion and pain control behaviour when faced with child's pain: the emotion regulatory role of parental pain-related attention-set shifting and heart rate variability. *Pain* 2019;160:322–33.

64 Vervoort T, Trost Z, Sütterlin S, *et al.* Emotion regulatory function of parent attention to child pain and associated implications for parental pain control behaviour. *Pain* 2014;155.

65 Simonse E, Mulder PGH, van Beek RHT. Analgesic effect of breast milk versus sucrose for analgesia during heel lance in late preterm infants. *Pediatrics* 2012;129:657–63.

66 Campbell-Yeo ML, Johnston CC, Joseph KS, *et al.* Cobedding and recovery time after heel lance in preterm twins: results of a randomized trial. *Pediatrics* 2012;130:500–6.

67 Taddio A, Shah V, Hancock R, *et al.* Effectiveness of sucrose analgesia in newborns undergoing painful medical procedures. *C Can Med Assoc J = J l'Association medicale Can* 2008;179:37–43.

68 Schwartz PJ, Garson AJ, Paul T, *et al.* Guidelines for the interpretation of the neonatal electrocardiogram. A task force of the European Society of Cardiology. *Eur Heart J* 2002;23:1329–44.

69 Fleming S, Thompson M, Stevens R, *et al.* Normal ranges of heart rate and respiratory rate in children from birth to 18 years of age: a systematic review of observational studies. *Lancet (London, England)* 2011;377:1011–8.

70 Adjei T, Purdy R, Jorge J, *et al.* A new method to measure inter-breath intervals in infants for the assessment of apnoea and respiratory dynamics. *medRxiv* 2021;:2021.05.29.21258043.

71 NICE. NICEimpact maternity and neonatal care. 2019.

72 Duley L, Uhm S, Oliver S. Top 15 UK research priorities for preterm birth. Lancet (London, England). 2014;383:2041–2.

73 Wielenga JM, Tume LN, Latour JM, *et al.* European neonatal intensive care nursing research priorities: an e-Delphi study. *Arch Dis Child Fetal Neonatal Ed* 2015;100:F66-71.

74 Franck LS, Oulton K, Bruce E. Parental involvement in neonatal pain management: an empirical and conceptual update. *J Nurs Scholarsh an Off Publ Sigma Theta Tau Int Honor Soc Nurs* 2012;44:45–54.

75 Johnston C, Barrington KJ, Taddio A, *et al.* Pain in Canadian NICUs: have we improved over the past 12 years? *Clin J Pain* 2011;27:225–32.

76 Onozawa K, Glover V, Adams D, *et al.* Infant massage improves mother-infant interaction for mothers with postnatal depression. *J Affect Disord* 2001;63:201–7.

77 Petersen I, Peltola T, Kaski S, *et al.* Depression, depressive symptoms and treatments in women who have recently given birth: UK cohort study. *BMJ Open* 2018;8:e022152.

78 Sharp H, Pickles A, Meaney M, *et al.* Frequency of infant stroking reported by mothers moderates the effect of prenatal depression on infant behavioural and physiological outcomes. *PLoS One* 2012;7:e45446.

79 Nicholls SG, Southern KW. Parental decision-making and acceptance of newborn bloodspot screening: an exploratory study. *PLoS One* 2013;8:e79441.

80 Harrington PM, Woodman C, Shannon WF. Low immunisation uptake: is the process the problem? *J Epidemiol Community Health* 2000;54:394–400.

81 Mills E, Jadad AR, Ross C, *et al.* Systematic review of qualitative studies exploring parental beliefs and attitudes toward childhood vaccination identifies common barriers to vaccination. *J Clin Epidemiol* 2005;58:1081–8.

82 Tickner S, Leman PJ, Woodcock A. Parents' views about pre-school immunization: an interview study in

For peer review only

For peer review only - http://bmjopen.bmj.com/site/about/guidelines.xhtml

southern England. *Child Care Health Dev* 2010;36:190–7.
83 Racine NM, Pillai Riddell RR, Flora DB, *et al.* Predicting preschool pain-related anticipatory distress: the relative contribution of longitudinal and concurrent factors. *Pain* 2016;157:1918–32.
84 Manzotti A, Cerritelli F, Esteves JE, *et al.* Dynamic touch reduces physiological arousal in preterm infants: A role for c-tactile afferents? *Dev Cogn Neurosci* 2019;39:100703.
85 Jönsson EH, Kotilahti K, Heiskala J, *et al.* Affective and non-affective touch evoke differential brain responses in 2-month-old infants. *Neuroimage* 2018;169:162–71.
86 Tuulari JJ, Scheinin NM, Lehtola S, *et al.* Neural correlates of gentle skin stroking in early infancy. *Dev Cogn Neurosci* 2019;35:36–41.

For peer review only

For peer review only - http://bmjopen.bmj.com/site/about/guidelines.xhtml

Inclusion Criteria

- Born at the John Radcliffe Hospital, Oxford or the Royal Devon and Exeter Hospital, Devon
- Born at or after 35+0 weeks' gestation with a postnatal age ≤7 days
- Require a clinical heel lance as part of clinical care
- Parents/guardians have given written informed consent for inclusion in the trial.

Exclusion Criteria

- Hypoxic Ischaemic Encephalopathy
- IVH > grade II
- Received any analgesics/sedatives in the last 24 hours
- Born with a congenital malformation or genetic condition known to affect neurological development
- Born to a mother with a history of substance abuse.

Randomisation (1:1 allocation ratio)
Web-based randomisation

30 min baseline data collection
(EEG, heart rate, oxygen saturation)

Group A (n=56)
Parental touch pre-heel lance

Group B (n=56)
Parental touch post-heel lance

Primary Outcome measures

(i) Magnitude of noxious-evoked brain activity evoked by heel lance.

Secondary Outcome measures

(i) Premature Infant Pain Profile – Revised (PIPP-R) score during the 30-second period after the heel lance.
(ii) Percentage of neonates who develop tachycardia in the 30 seconds post-heel lance.
(iii) Parent questionnaire assessing anxiety.

Exploratory Outcome measures

(i) Changes in background brain activity during touch intervention.
(ii) Time taken for heart rate to return to baseline post-heel lance.
(iii) Post procedural respiratory dynamics and incidence of apnoea.
(iv) Parental questionnaire assessing parental anxiety, distress, and experience of research.

For peer review only - http://bmjopen.bmj.com/site/about/guidelines.xhtml

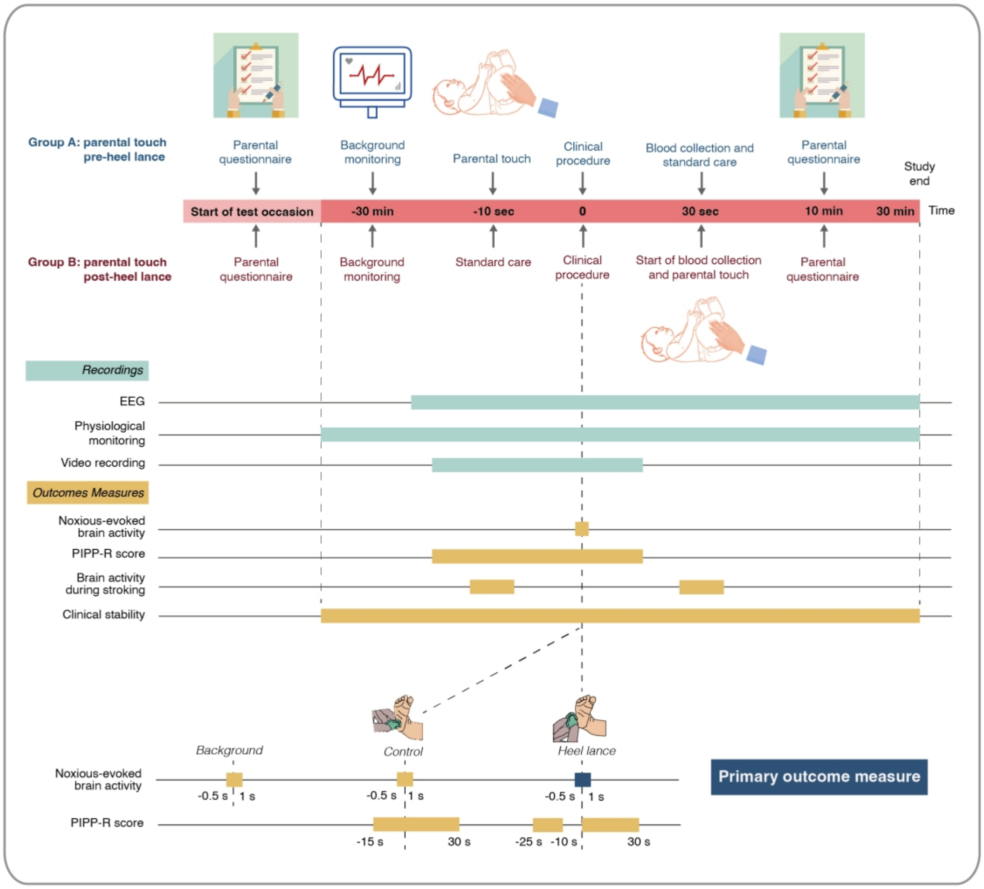

Trial procedures.

445x406mm (118 x 118 DPI)

Study protocol: a multicentre, randomised controlled trial to investigate the effects of parental touch on relieving acute procedural pain in neonates (Petal)

STANDARD PROTOCOL ITEMS: RECOMMENDATIONS FOR INTERVENTIONAL TRIALS

SPIRIT 2013 Checklist: Recommended items to address in a clinical trial protocol and related documents*

Included in manuscri	Section/item	ItemNo	Description	Comment
	**Administrative information**			
X	Title	1	Descriptive title identifying the study design, population, interventions, and, if applicable, trial acronym	
X	Trial registration	2a	Trial identifier and registry name. If not yet registered, name of intended registry	
		2b	All items from the World Health Organization Trial Registration Data Set	
X	Protocol version	3	Date and version identifier	
X	Funding	4	Sources and types of financial, material, and other support	
X	Roles and responsibilities	5a	Names, affiliations, and roles of protocol contributors	
		5b	Name and contact information for the trial sponsor	
		5c	Role of study sponsor and funders, if any, in study design; collection, management, analysis, and interpretation of data; writing of the report; and the decision to submit the report for publication, including whether they will have ultimate authority over any of these activities	

For peer review only

Study protocol: a multicentre, randomised controlled trial to investigate the effects of parental touch on relieving acute procedural pain in neonates (Petal)

		5d	Composition, roles, and responsibilities of the coordinating centre, steering committee, endpoint adjudication committee, data management team, and other individuals or groups overseeing the trial, if applicable (see Item 21a for data monitoring committee)
	**Introduction**		
X	Background and rationale	6a	Description of research question and justification for undertaking the trial, including summary of relevant studies (published and unpublished) examining benefits and harms for each intervention
		6b	Explanation for choice of comparators
X	Objectives	7	Specific objectives or hypotheses
X	Trial design	8	Description of trial design including type of trial (eg, parallel group, crossover, factorial, single group), allocation ratio, and framework (eg, superiority, equivalence, noninferiority, exploratory)
	**Methods: Participants, interventions, and outcomes**		
X	Study setting	9	Description of study settings (eg, community clinic, academic hospital) and list of countries where data will be collected. Reference to where list of study sites can be obtained
X	Eligibility criteria	10	Inclusion and exclusion criteria for participants. If applicable, eligibility criteria for study centres and individuals who will perform the interventions (eg, surgeons, psychotherapists)
X	Interventions	11a	Interventions for each group with sufficient detail to allow replication, including how and when they will be administered

For peer review only - http://bmjopen.bmj.com/site/about/guidelines.xhtml

Study protocol: a multicentre, randomised controlled trial to investigate the effects of parental touch on relieving acute procedural pain in neonates (Petal)

		11b	Criteria for discontinuing or modifying allocated interventions for a given trial participant (eg, drug dose change in response to harms, participant request, or improving/worsening disease)
		11c	Strategies to improve adherence to intervention protocols, and any procedures for monitoring adherence (eg, drug tablet return, laboratory tests)
		11d	Relevant concomitant care and interventions that are permitted or prohibited during the trial
X	Outcomes	12	Primary, secondary, and other outcomes, including the specific measurement variable (eg, systolic blood pressure), analysis metric (eg, change from baseline, final value, time to event), method of aggregation (eg, median, proportion), and time point for each outcome. Explanation of the clinical relevance of chosen efficacy and harm outcomes is strongly recommended
X	Participant timeline	13	Time schedule of enrolment, interventions (including any run-ins and washouts), assessments, and visits for participants. A schematic diagram is highly recommended (see Figure)
X	Sample size	14	Estimated number of participants needed to achieve study objectives and how it was determined, including clinical and statistical assumptions supporting any sample size calculations
X	Recruitment	15	Strategies for achieving adequate participant enrolment to reach target sample size

Methods: Assignment of interventions (for controlled trials)

Allocation:

Study protocol: a multicentre, randomised controlled trial to investigate the effects of parental touch on relieving acute procedural pain in neonates (Petal)

X	Sequence generation	16a	Method of generating the allocation sequence (eg, computer-generated random numbers), and list of any factors for stratification. To reduce predictability of a random sequence, details of any planned restriction (eg, blocking) should be provided in a separate document that is unavailable to those who enrol participants or assign interventions
	Allocation concealment mechanism	16b	Mechanism of implementing the allocation sequence (eg, central telephone; sequentially numbered, opaque, sealed envelopes), describing any steps to conceal the sequence until interventions are assigned
	Implementation	16c	Who will generate the allocation sequence, who will enrol participants, and who will assign participants to interventions
X	Blinding (masking)	17a	Who will be blinded after assignment to interventions (eg, trial participants, care providers, outcome assessors, data analysts), and how
		17b	If blinded, circumstances under which unblinding is permissible, and procedure for revealing a participant's allocated intervention during the trial
	**Methods: Data collection, management, and analysis**		
X	Data collection methods	18a	Plans for assessment and collection of outcome, baseline, and other trial data, including any related processes to promote data quality (eg, duplicate measurements, training of assessors) and a description of study instruments (eg, questionnaires, laboratory tests) along with their reliability and validity, if known. Reference to where data collection forms can be found, if not in the protocol

Study protocol: a multicentre, randomised controlled trial to investigate the effects of parental touch on relieving acute procedural pain in neonates (Petal)

		18b	Plans to promote participant retention and complete follow-up, including list of any outcome data to be collected for participants who discontinue or deviate from intervention protocols
X	Data management	19	Plans for data entry, coding, security, and storage, including any related processes to promote data quality (eg, double data entry; range checks for data values). Reference to where details of data management procedures can be found, if not in the protocol
X	Statistical methods	20a	Statistical methods for analysing primary and secondary outcomes. Reference to where other details of the statistical analysis plan can be found, if not in the protocol
		20b	Methods for any additional analyses (eg, subgroup and adjusted analyses)
		20c	Definition of analysis population relating to protocol non-adherence (eg, as randomised analysis), and any statistical methods to handle missing data (eg, multiple imputation)
	**Methods: Monitoring**		
X	Data monitoring	21a	Composition of data monitoring committee (DMC); summary of its role and reporting structure; statement of whether it is independent from the sponsor and competing interests; and reference to where further details about its charter can be found, if not in the protocol. Alternatively, an explanation of why a DMC is not needed
		21b	Description of any interim analyses and stopping guidelines, including who will have access to these interim results and make the final decision to terminate the trial

Study protocol: a multicentre, randomised controlled trial to investigate the effects of parental touch on relieving acute procedural pain in neonates (Petal)

X	Harms	22	Plans for collecting, assessing, reporting, and managing solicited and spontaneously reported adverse events and other unintended effects of trial interventions or trial conduct	
	Auditing	23	Frequency and procedures for auditing trial conduct, if any, and whether the process will be independent from investigators and the sponsor	The project management group (RS, RP, AB, AH, DC, MC, FM) meets monthly throughout the trial to oversee trial conduct and recruitment.
	**Ethics and dissemination**			
X	Research ethics approval	24	Plans for seeking research ethics committee/institutional review board (REC/IRB) approval	
	Protocol amendments	25	Plans for communicating important protocol modifications (eg, changes to eligibility criteria, outcomes, analyses) to relevant parties (eg, investigators, REC/IRBs, trial participants, trial registries, journals, regulators)	The Investigator will submit and, where necessary, obtain approval from the relevant parties for all substantial amendments to the original approved documents.
X	Consent or assent	26a	Who will obtain informed consent or assent from potential trial participants or authorised surrogates, and how (see Item 32)	
		26b	Additional consent provisions for collection and use of participant data and biological specimens in ancillary studies, if applicable	
X	Confidentiality	27	How personal information about potential and enrolled participants will be collected, shared, and maintained in order to protect confidentiality before, during, and after the trial	

For peer review only - http://bmjopen.bmj.com/site/about/guidelines.xhtml

Study protocol: a multicentre, randomised controlled trial to investigate the effects of parental touch on relieving acute procedural pain in neonates (Petal)

X	Declaration of interests	28	Financial and other competing interests for principal investigators for the overall trial and each study site	
	Access to data	29	Statement of who will have access to the final trial dataset, and disclosure of contractual agreements that limit such access for investigators	Only the project team will have access to the final trial dataset. After the publication of results, anonymised data can be made available to other researchers upon reasonable request.
	Ancillary and post-trial care	30	Provisions, if any, for ancillary and post-trial care, and for compensation to those who suffer harm from trial participation	The University of Oxford has a specialist insurance policy in place which would operate in the event of any participant suffering harm as a result of their involvement in the research (Newline Underwriting Management Ltd, at Lloyd's of London). NHS indemnity operates in respect of the clinical treatment which is provided.
X	Dissemination policy	31a	Plans for investigators and sponsor to communicate trial results to participants, healthcare professionals, the public, and other relevant groups (eg, via publication, reporting in results databases, or other data sharing arrangements), including any publication restrictions	

Study protocol: a multicentre, randomised controlled trial to investigate the effects of parental touch on relieving acute procedural pain in neonates (Petal)

	31b	Authorship eligibility guidelines and any intended use of professional writers	
	31c	Plans, if any, for granting public access to the full protocol, participant-level dataset, and statistical code	
**Appendices**			
Informed consent materials	32	Model consent form and other related documentation given to participants and authorised surrogates	
Biological specimens	33	Plans for collection, laboratory evaluation, and storage of biological specimens for genetic or molecular analysis in the current trial and for future use in ancillary studies, if applicable	No biological specimens are collected.

*It is strongly recommended that this checklist be read in conjunction with the SPIRIT 2013 Explanation & Elaboration for important clarification on the items. Amendments to the protocol should be tracked and dated. The SPIRIT checklist is copyrighted by the SPIRIT Group under the Creative Commons "Attribution-NonCommercial-NoDerivs 3.0 Unported" license.

For peer review only

Parental touch trial

Parent Information Leaflet

For peer review only

John Radcliffe Hospital

UNIVERSITY OF OXFORD

DEPARTMENT OF PAEDIATRICS
Medical Sciences Division

Oxford University Hospitals NHS
NHS Foundation Trust

For peer review only - http://bmjopen.bmj.com/site/about/guidelines.xhtml

You and your child are eligible to take part in a research study. Before you decide, it is important that you understand why the research is being done and what it involves. Please read the following information and ask us if anything is unclear or if you would like more information.

Study title: **Par**ent**al** touch trial (*Petal*)
A randomised controlled trial to investigate the effects of parental touch on relieving acute procedural pain in neonates

1. What is the purpose of the study?

Babies in hospital often require clinical procedures as part of their routine medical treatment. As babies cannot tell us how much these procedures hurt, it is difficult to make sure that they are receiving the right pain-relief treatments. We know babies can experience discomfort and pain, and we have developed a method to measure changes in brain activity that occur when a baby undergoes a clinical procedure. We also know that babies display specific facial expressions when they are in pain and that their heart rate and breathing rate can increase.

Touch is important for parent and child bonding, and research in adults has shown that stroking the skin at the right speed can reduce pain experienced during some procedures. Stroking and parental touch activates special fibres in the skin that we think can make procedures feel less painful. Some studies have shown that close skin-to-skin contact between babies and their parents can reduce pain during procedures (such as blood tests).

The aim of this research is to understand if parental touch can reduce how much pain their babies experience during a blood test. We also want to know how parents feel when they stroke their baby's leg during a blood test. We would like to see if there are any differences in how a baby responds if a parent strokes their child's leg before or after a blood test.

2. Why have I been invited?

You and your child have been invited to take part in this study because your child requires a blood test for clinical purposes. We are recruiting parents and their children who were born at least 35 weeks gestation, who need to have a clinical blood test. We hope to recruit in total 112 babies.

3. Do we have to take part?

No, it is your decision whether or not you and your child take part. If you decide to take part, you will be asked to sign a consent form. If you decide you do not want to take part, this will not affect your child's care.

If you decide you would like to take part, you can change your mind at any time and withdraw you and your child from the study by telling the research team. You do not have to give a reason. You will be asked if we can use the data/images that have already been collected for analysis and if we can publish the anonymised results.

4. What is involved in the study?

We would like to understand how parental touch (in the form of stroking) may affect how babies respond to a clinically-required blood test. **No blood tests will be carried out solely for research purposes.** We will only study your child during a blood test that is needed for clinical purposes. Blood tests will be scheduled according to clinical need and thus you may have less than 24 hours to consider your participation in this trial. Blood tests will be completed in the routine way. On some occasions more than one heel lance is required to collect a sufficient blood sample. If this is the case, the research monitoring equipment will not be removed from your baby between heel lances in order to ensure that we do not interfere with the clinical procedures. As part of the study, we will also administer a 'sham' heel lance: this is not a real blood test and will not pierce your baby's skin or cause any pain.

For peer review only - http://bmjopen.bmj.com/site/about/guidelines.xhtml

This is a control stimulus to simulate the blood test without the 'painful' part. The heel lance is placed against your baby's foot but angled away so that the sharp fires into the air rather than the foot. The study will not interfere with your child's clinical care, nor will there be any delay if an emergency procedure is required.

As part of the study, we will ask you to stroke your child's leg for 10 seconds before, or 10 seconds after, their blood test. Half of the babies in the study will be stroked by a parent before the blood test, and half will be stroked afterwards. Before the study, your baby's details will be entered into a computer programme that will randomly select whether you should stroke your baby before or after the blood test. Where required, we will demonstrate on a doll the speed, location and duration of the stroking. Before we start the study we will make sure you know how to complete the stroking in the correct way. As part of the study, we will also ask you to complete a short questionnaire; the first part will be before the study, the second part at the end. The questionnaire overall should take approximately 15 minutes.

We will assess your child's responses to the blood test by measuring their brain activity. We will also video your child's face, and measure other responses such heart rate, breathing rate and oxygen saturations. We will monitor your child before, during and after the blood test for approximately one hour.

We will use the following recording measures for your child:

Measuring brain activity
Electroencephalography (EEG)**:** EEG is a portable imaging system to measure brain activity. It involves gently placing electrodes (small discs) on the head using a paste that can be washed off with soap and water. EEG is routinely used on the neonatal unit, children's wards and clinics.

Measuring other responses
Vital sign monitoring: Small adhesive discs will be placed on your child's chest to measure changes in breathing rate and heart rate (this is called an ECG).

Videoing your child: We will also video your child during the study. This is so that we can assess changes in facial expression and body movements, and record the exact timing of the blood test.

We may ask if you are happy for us to use these images for teaching, publicity and/or in scientific journals. If you agree, we will take separate consent for this as your child's face would be present in the video footage. This is an optional part of the study and is not essential. If we do not use the images, this will not affect your child's care or stop your child participating in this research.

5. Are there any additional risks or benefits for my child?
Recording a video of your child is non-invasive and does not present any risk. EEG and ECG have been used clinically for over 20 years without any adverse effects. All studies have a dedicated team of healthcare professionals and researchers that will ensure the safety of your child at all times. We are not aware of any risks for your child taking part in this study.

The research data collected will not be routinely reviewed by a doctor. If any clinically significant findings are identified at the time of the study then the research team will report these to the clinical care team for further review.

There are no direct benefits of taking part in the study. This study is designed to gather information, to help improve the care we provide for babies in the future. If your child becomes distressed, the research study will be paused or stopped. Any clinically required procedures will still go ahead if the clinician looking after your child feels that this is appropriate.

For peer review only - http://bmjopen.bmj.com/site/about/guidelines.xhtml

6. What information will be collected about me and my child?

We will collect relevant medical (e.g. age), environmental (e.g. medical ward where study was conducted), demographic (e.g. post code) and social (e.g. ethnicity) information about your child from their medical notes. This information helps us to determine which factors may influence the way a baby copes with pain. We will also collect vital sign data (such as heart rate and breathing rate), recordings of their facial expressions and body movements and changes in brain activity caused by the blood test. We will ask you to complete a questionnaire.

All information and videos that are collected during this research study will be stored confidentially. Each baby will be allocated a study number which will be used to label the data. This study forms part of an educational programme.

7. What will happen to the results?

Results will be analysed and published in a journal. All publications will be made available on our website **https://neuroimaging.paediatrics.ox.ac.uk**. The findings may also be used for teaching or academic research presentations. No identifying information will be presented about you or your child, unless you have provided specific consent for us to use videos or images of your child in this way.

8. What will happen to my data and my child's data?

We will use the information about you and your child in order to conduct this study. Research is a task that we perform in the public interest. The University of Oxford, as Sponsor, is the data controller. This means that we, as University of Oxford researchers and collaborators, are responsible for looking after the information collected and using it properly. We will use the minimum personally-identifiable information possible. We will keep identifiable information about you and your child for up to 12 months after the study has finished. This excludes any research documents with personal information, such as consent forms and facial expression recordings, which will be held securely at the University of Oxford for 21 years after the end of the study.

Data protection regulation provides you with control over your personal data and how it is used. When you agree to your information being used in research, however, some of those rights may be limited in order for the research to be reliable and accurate. Further information about your rights with respect to your personal data is available at http://www.admin.ox.ac.uk/councilsec/compliance/gdpr/individualrights/. You can find out more about how we use your information from the contacts in section 12.

Research data may be shared with other researchers, both here and abroad. Responsible members of the University of Oxford and Oxford University Hospitals NHS Trust may be given access to data for monitoring and/or audit of the study to ensure we are complying with regulations.

9. Who is organising and funding this research?

This study is sponsored by the University of Oxford and has been funded by the Wellcome Trust and the charity BLISS. Your doctor will not be paid for including you in this study.

10. Who has reviewed the study?

All research in the NHS is looked at by an independent group of people, called a Research Ethics Committee, to protect participants' interests. This study has been reviewed and given favourable opinion by the London - South East Research Ethics Committee.

11. Comments or concerns during the study

The University has arrangements in place to provide for harm arising from participation in the study for which the University is the Research Sponsor. NHS indemnity operates in respect of the clinical treatment with which your child is provided. If you wish to complain about any aspect of the way in

For peer review only - http://bmjopen.bmj.com/site/about/guidelines.xhtml

which you have been approached or treated during the course of this study, you should contact Prof Rebeccah Slater (details below) or the University of Oxford Clinical Trials and Research Governance (CTRG) office (tel: 01865 616480, email: ctrg@admin.ox.ac.uk).

The Patient Advisory Liaison Service (PALS) is a confidential NHS service that can provide you with support for any complaints or queries you may have regarding the care you receive as an NHS patient. PALS is unable to provide information about this research study. The John Radcliffe Hospital PALS team can be contacted on: Tel: 01865 221473, Email: PALS@ouh.nhs.uk, http://www.ouh.nhs.uk/patient-guide/pals.aspx.

12. Participation in future research

As we are interested in how your child's response to pain changes as they grow, we may ask if we can contact you in the future, to ask if you would be happy for your child to take part in other similar research studies run by our research team. If you agree that we can contact you about other research studies we will ask you to complete an optional additional consent item on the Consent Form used when you agree for your child to participate in the study. We will record your contact details, and these will be kept in a separate electronic database from the rest of the research data. This database is password-protected and can only be accessed by members of the research team.

Your contact details will not be passed onto anyone outside of the research team. All contact will come from the research team in the first instance. You can opt-out of this at any point by contacting Prof Rebeccah Slater (details below). Your agreement for us to contact you does not form any obligation to participate in future research.

What will happen to my data?

If you have provided optional additional consent to be contacted about future studies, we will store your contact details indefinitely unless you choose to opt-out at any point.

13. Contact for further information

Chief Investigator: Dr Eleri Adams eleri.adams@ouh.nhs.uk 01865 221356
Principal Investigator: Prof Rebeccah Slater rebeccah.slater@paediatrics.ox.ac.uk 01865 234229

You can also access further information about research or parent support from the following groups:

- BLISS: UK based charity https://www.bliss.org.uk/
- SSNAP (Support for the Sick Newborn And their Parents): Oxford based charity https://www.ssnap.org.uk

Representative image removed for publication

Picture shows example of an EEG study.

Thank you for reading this leaflet.

For peer review only

For peer review only - http://bmjopen.bmj.com/site/about/guidelines.xhtml

Parental touch trial

Parent Information Leaflet

Royal Devon and Exeter Hospital

UNIVERSITY OF OXFORD
DEPARTMENT OF PAEDIATRICS
Medical Sciences Division

NHS
Royal Devon
University Healthcare
NHS Foundation Trust

For peer review only - http://bmjopen.bmj.com/site/about/guidelines.xhtml

You and your child are eligible to take part in a research study. Before you decide, it is important that you understand why the research is being done and what it involves. Please read the following information and ask us if anything is unclear or if you would like more information.

Study title: **Par**ent**al** touch trial (*Petal*)
A randomised controlled trial to investigate the effects of parental touch on relieving acute procedural pain in neonates

1. What is the purpose of the study?

Babies in hospital often require clinical procedures as part of their routine medical treatment. As babies cannot tell us how much these procedures hurt, it is difficult to make sure that they are receiving the right pain-relief treatments. We know babies can experience discomfort and pain, and we have developed a method to measure changes in brain activity that occur when a baby undergoes a clinical procedure. We also know that babies display specific facial expressions when they are in pain and that their heart rate and breathing rate can increase.

Touch is important for parent and child bonding, and research in adults has shown that stroking the skin at the right speed can reduce pain experienced during some procedures. Stroking and parental touch activates special fibres in the skin that we think can make procedures feel less painful. Some studies have shown that close skin-to-skin contact between babies and their parents can reduce pain during procedures (such as blood tests).

The aim of this research is to understand if parental touch can reduce how much pain their babies experience during a blood test. We also want to know how parents feel when they stroke their baby's leg during a blood test. We would like to see if there are any differences in how a baby responds if a parent strokes their child's leg before or after a blood test.

2. Why have I been invited?

You and your child have been invited to take part in this study because your child requires a blood test for clinical purposes. We are recruiting parents and their children who were born at least 35 weeks gestation, who need to have a clinical blood test. We hope to recruit in total 112 babies.

3. Do we have to take part?

No, it is your decision whether or not you and your child take part. If you decide to take part, you will be asked to sign a consent form. If you decide you do not want to take part, this will not affect your child's care.

If you decide you would like to take part, you can change your mind at any time and withdraw you and your child from the study by telling the research team. You do not have to give a reason. You will be asked if we can use the data/images that have already been collected for analysis and if we can publish the anonymised results.

4. What is involved in the study?

We would like to understand how parental touch (in the form of stroking) may affect how babies respond to a clinically-required blood test. **No blood tests will be carried out solely for research purposes.** We will only study your child during a blood test that is needed for clinical purposes. Blood tests will be scheduled according to clinical need and thus you may have less than 24 hours to consider your participation in this trial. Blood tests will be completed in the routine way. On some occasions more than one heel lance is required to collect a sufficient blood sample. If this is the case, the research monitoring equipment will not be removed from your baby between heel lances in order to ensure that we do not interfere with the clinical procedures. As part of the study, we will also administer a 'sham' heel lance: this is not a real blood test and will not pierce your baby's skin

For peer review only - http://bmjopen.bmj.com/site/about/guidelines.xhtml

or cause any pain. This is a control stimulus to simulate the blood test without the 'painful' part. The heel lance is placed against your baby's foot but angled away so that the sharp fires into the air rather than the foot. The study will not interfere with your child's clinical care, nor will there be any delay if an emergency procedure is required.

As part of the study, we will ask you to stroke your child's leg for 10 seconds before, or 10 seconds after, their blood test. Half of the babies in the study will be stroked by a parent before the blood test, and half will be stroked afterwards. Before the study, your baby's details will be entered into a computer programme that will randomly select whether you should stroke your baby before or after the blood test. Where required we will demonstrate on a doll the speed, location and duration of the stroking. Before we start the study we will make sure you know how to complete the stroking in the correct way. As part of the study, we will also ask you to complete a short questionnaire; the first part will be before the study, the second part at the end. The questionnaire overall should take approximately 15 minutes.

We will assess your child's responses to the blood test by measuring their brain activity. We will also video your child's face, and measure other responses such heart rate, breathing rate and oxygen saturations. We will monitor your child before, during and after the blood test for approximately one hour.

We will use the following recording measures for your child:

Measuring brain activity
Electroencephalography (EEG)**:** EEG is a portable imaging system to measure brain activity. It involves gently placing electrodes (small discs) on the head using a paste that can be washed off with soap and water. EEG is routinely used on the neonatal unit, children's wards and clinics.

Measuring other responses
Vital sign monitoring: Small adhesive discs will be placed on your child's chest to measure changes in breathing rate and heart rate (this is called an ECG).

Videoing your child: We will also video your child during the study. This is so that we can assess changes in facial expression and body movements, and record the exact timing of the blood test.

We may ask if you are happy for us to use these images for teaching, publicity and/or in scientific journals. If you agree, we will take separate consent for this as your child's face would be present in the video footage. This is an optional part of the study and is not essential. If we do not use the images, this will not affect your child's care or stop your child participating in this research.

5. Are there any additional risks or benefits for my child?

Recording a video of your child is non-invasive and does not present any risk. EEG and ECG have been used clinically for over 20 years without any adverse effects. All studies have a dedicated team of healthcare professionals and researchers that will ensure the safety of your child at all times. We are not aware of any risks for your child taking part in this study.

The research data collected will not be routinely reviewed by a doctor. If any clinically significant findings are identified at the time of the study then the research team will report these to the clinical care team for further review.

There are no direct benefits of taking part in the study. This study is designed to gather information, to help improve the care we provide for babies in the future. If your child becomes distressed, the research study will be paused or stopped. Any clinically required procedures will still go ahead if the clinician looking after your child feels that this is appropriate.

For peer review only - http://bmjopen.bmj.com/site/about/guidelines.xhtml

6. What information will be collected about me and my child?

We will collect relevant medical (e.g. age), environmental (e.g. medical ward where study was conducted), demographic (e.g. post code) and social (e.g. ethnicity) information about your child from their medical notes. This information helps us to determine which factors may influence the way a baby copes with pain. We will also collect vital sign data (such as heart rate and breathing rate), recordings of their facial expressions and body movements and changes in brain activity caused by the blood test. We will ask you to complete a questionnaire.

All information and videos that are collected during this research study will be stored confidentially. Each baby will be allocated a study number which will be used to label the data. This study forms part of an educational programme.

7. What will happen to the results?

Results will be analysed and published in a journal. All publications will be made available on our website **https://neuroimaging.paediatrics.ox.ac.uk**. The findings may also be used for teaching or academic research presentations. No identifying information will be presented about you or your child, unless you have provided specific consent for us to use videos or images of your child in this way.

8. What will happen to my data and my child's data?

We will use the information about you and your child in order to conduct this study. Research is a task that we perform in the public interest. The University of Oxford, as Sponsor, is the data controller. This means that we, as University of Oxford researchers and collaborators, are responsible for looking after the information collected and using it properly. We will use the minimum personally-identifiable information possible. We will keep identifiable information about you and your child for up to 12 months after the study has finished. This excludes any research documents with personal information, such as consent forms and facial expression recordings, which will be held securely at the University of Oxford for 21 years after the end of the study.

Data protection regulation provides you with control over your personal data and how it is used. When you agree to your information being used in research, however, some of those rights may be limited in order for the research to be reliable and accurate. Further information about your rights with respect to your personal data is available at http://www.admin.ox.ac.uk/councilsec/compliance/gdpr/individualrights/. You can find out more about how we use your information from the contacts in section 12.

Research data may be shared with other researchers, both here and abroad. Responsible members of the University of Oxford and Oxford University Hospitals NHS Trust may be given access to data for monitoring and/or audit of the study to ensure we are complying with regulations.

9. Who is organising and funding this research?

This study is sponsored by the University of Oxford and has been funded by the Wellcome Trust and the charity BLISS. Your doctor will not be paid for including you in this study.

10. Who has reviewed the study?

All research in the NHS is looked at by an independent group of people, called a Research Ethics Committee, to protect participants' interests. This study has been reviewed and given favourable opinion by the London - South East Research Ethics Committee.

11. Comments or concerns during the study

The University has arrangements in place to provide for harm arising from participation in the study for which the University is the Research Sponsor. NHS indemnity operates in respect of the clinical

For peer review only - http://bmjopen.bmj.com/site/about/guidelines.xhtml

treatment with which your child is provided. If you wish to complain about any aspect of the way in which you have been approached or treated during the course of this study, you should contact Dr Ravi Poorun (details below) or the University of Oxford Clinical Trials and Research Governance (CTRG) office (tel: 01865 616480, email: ctrg@admin.ox.ac.uk).

The Patient Advisory Liaison Service (PALS) is a confidential NHS service that can provide you with support for any complaints or queries you may have regarding the care you receive as an NHS patient. PALS is unable to provide information about this research study. The Royal Devon and Exeter Hospital PALS team can be contacted on: Tel: 01392 402093, Email: rde-tr.PALS@nhs.net, https://www.rdehospital.nhs.uk/patients-visitors/patient-advice-liaison-service-pals/#

12. Participation in future research

As we are interested in how your child's response to pain changes as they grow, we may ask if we can contact you in the future, to ask if you would be happy for your child to take part in other similar research studies run by our research team. If you agree that we can contact you about other research studies we will ask you to complete an optional additional consent item on the Consent Form used when you agree for your child to participate in the study. We will record your contact details, and these will be kept in a separate electronic database from the rest of the research data. This database is password-protected and can only be accessed by members of the research team.

Your contact details will not be passed onto anyone outside of the research team. All contact will come from the research team in the first instance. You can opt-out of this at any point by contacting Prof Rebeccah Slater (details below). Your agreement for us to contact you does not form any obligation to participate in future research.

What will happen to my data?

If you have provided optional additional consent to be contacted about future studies, we will store your contact indefinitely unless you choose to opt-out at any point.

13. Contact for further information

Chief Investigator: Dr Eleri Adams eleri.adams@ouh.nhs.uk 01865 221356
Principal Investigator: Dr Ravi Poorun r.poorun@exeter.ac.uk 01392 406980

You can also access further information about research or parent support from the following groups:

- BLISS: UK based charity https://www.bliss.org.uk/
- SSNAP (Support for the Sick Newborn And their Parents): Oxford based charity https://www.ssnap.org.uk

Representative image removed for publication

Picture shows example of an EEG study.

Thank you for reading this leaflet.

For peer review only - http://bmjopen.bmj.com/site/about/guidelines.xhtml

Consent Form (Oxford)

Petal Parental touch trial

Study ID: | | | | | | | | | |

Infant's name:

Study Title: Parental touch trial (Petal)

Chief Investigator: Dr Eleri Adams **Principal Investigator:** Prof Rebeccah Slater

Please initial each box

Please complete in black ballpoint pen.

1	I confirm that I have read and understood the information sheet (**v. , dated / /)**, for the above study. I have had the opportunity to ask questions and have had these answered satisfactorily.	☐
2	I understand that my participation and my child's participation is voluntary and that me and my child are free to withdraw at any time, without giving any reason, without our medical care or legal rights being affected.	☐
3	I understand that relevant sections of my child's medical notes and data collected during the study may be looked at by individuals from the University of Oxford or Oxford University Hospitals NHS Trust, where it is relevant to my child's taking part in this research. I give permission for these individuals to access to my child's records.	☐
4	I agree to my child being videoed during the study. I understand that recorded images will not be used for public use, only analysis. No identifiable information, including video recordings or imaging, will be used in any publications/presentations. Only anonymised data will be published or presented at meetings.	☐
5	I agree for the collected data to be used for teaching or academic research presentations.	☐
6	I agree to me and my child taking part in the above study.	☐
7	I agree to complete a parental questionnaire related to my child's study.	☐

OPTIONAL

8	I consent to being approached in the future about other similar research studies, by the research team, that my child may be eligible for. I understand that agreeing to be contacted does not oblige me or my child to participate in any further studies.	☐
9	I agree to the images/videos of my child recorded during this study being used for publications and presentations.	☐

Name of parent: ______________________

Relationship to baby: ______________________

Signature: ______________________

Date: ______________________

Name of investigator taking consent: ______________________

Signature: ______________________

Date: ______________________

1 to be kept as part of the study documentation (original)
1 copy for parent
1 copy with hospital notes

For peer review only - http://bmjopen.bmj.com/site/about/guidelines.xhtml

Consent Form (Exeter)

Study ID: | | | | | | | | |

Infant's name:

Study Title: Parental touch trial (Petal)

Chief Investigator: Dr Eleri Adams **Principal Investigator:** Dr Ravi Poorun

Please initial each box

Please complete in black ballpoint pen.

1	I confirm that I have read and understood the information sheet (**v. , dated / /)**, for the above study. I have had the opportunity to ask questions and have had these answered satisfactorily.	☐
2	I understand that my participation and my child's participation is voluntary and that me and my child are free to withdraw at any time, without giving any reason, without our medical care or legal rights being affected.	☐
3	I understand that relevant sections of my child's medical notes and data collected during the study may be looked at by individuals from the University of Oxford, Oxford University Hospitals NHS Trust or Royal Devon University Healthcare NHS Foundation Trust, where it is relevant to my child's taking part in this research. I give permission for these individuals to access to my child's records.	☐
4	I agree to my child being videoed during the study. I understand that recorded images will not be used for public use, only analysis. No identifiable information, including video recordings or imaging, will be used in any publications/presentations. Only anonymised data will be published or presented at meetings.	☐
5	I agree for the collected data to be used for teaching or academic research presentations.	☐
6	I agree to me and my child taking part in the above study.	☐
7	I agree to complete a parental questionnaire related to my child's study.	☐

OPTIONAL

8	I consent to being approached in the future about other similar research studies, by the research team, that my child may be eligible for. I understand that agreeing to be contacted does not oblige me or my child to participate in any further studies.	☐
9	I agree to the images/videos of my child recorded during this study being used for publications and presentations.	☐

Name of parent: ______________________

Relationship to baby: ______________________

Signature: ______________________

Date: ______________________

Name of investigator taking consent: ______________________

Signature: ______________________

Date: ______________________

1 to be kept as part of the study documentation (original)
1 copy for parent
1 copy with hospital notes
1 copy with PI

For peer review only - http://bmjopen.bmj.com/site/about/guidelines.xhtml
